# Shared representations in brains and models reveal a two-route cortical organization during scene perception

**DOI:** 10.1038/s42003-026-10169-0

**Published:** 2026-05-07

**Authors:** Pablo Marcos-Manchón, Lluís Fuentemilla

**Affiliations:** 1 Department of Cognition, Development and Education Psychology, Faculty of Psychology, University of Barcelona, Barcelona, Spain; 2 Institute of Neurosciences, University of Barcelona, Barcelona, Spain; 3 Bellvitge Institute for Biomedical Research, Barcelona, Spain

**Keywords:** Neural encoding, Network models, Perception

## Abstract

The brain transforms visual inputs into cortical representations that support diverse cognitive and behavioral goals. Characterizing how this information is organized and routed across the human brain is essential for understanding how we process complex visual scenes. Here, we applied representational similarity analysis to 7T fMRI data collected during natural scene viewing. We quantified representational geometry shared across individuals and compared it to hierarchical features from vision and language neural networks across model layers. By integrating these comparisons with representational connectivity between cortical regions, we identified two distinct processing routes: a ventromedial pathway specialized for scene layout and environmental context, and a lateral occipitotemporal pathway selective for animate content. Vision models aligned with shared structure in both routes, whereas language models corresponded primarily with the lateral pathway and showed negative alignment in early visual and ventral cortex. These findings refine classical visual-stream models by revealing a distributed cortical network with separable representational routes for context and animate content during scene perception.

## Abstract (web summary)

Representational similarity analysis of human brain fMRI during natural scene viewing reveals two cortical routes: a ventromedial pathway for scene context and layout, and a lateral occipitotemporal pathway tuned to animate content.

## Introduction

Sensory inputs arrive at the brain as physical signals, but perception depends on how those signals are converted into structured patterns of cortical activity^1,2^. In vision, this conversion spans multiple stages, from early visual cortex to downstream regions whose activity patterns encode objects, scenes, and higher-order interactions^2–5^. Characterizing how these representations are transformed across the cortex is central to understanding how the brain performs this computation: identifying which regions encode specific aspects of the sensory input and mapping the networks that route this information between them.

Naturalistic neuroimaging provides a way to approach this problem. When different individuals view the same stimuli, activity patterns show reliable synchrony across widespread regions^6–8^. This inter-subject synchrony is interpreted as evidence that, despite individual variability, a substantial stimulus-locked component is encoded similarly across brains. This shared component can be used to localize regions that reliably encode stimulus-driven information across individuals^9,10^. Representational geometry formalizes this idea by describing each region in terms of pairwise similarities among stimulus-evoked responses, enabling comparisons across individuals and regions, and relating representational variance to stimulus properties that organize it^11–13^.

Representational geometry also enables direct comparisons between brains and deep neural networks (DNNs). Across vision and language models, internal activations can resemble cortical representations when both systems process the same stimuli^14–16^, reflecting shared organizing dimensions that group stimuli according to similar principles^17^. In the visual domain, vision models show graded correspondence with cortical organization, with deeper layers capturing increasingly complex shape- and object-based features^18–21^. Complementarily, language models capture semantic regularities that abstract away from visual details, with recent work identifying correspondence in higher-level ventral and lateral regions where perceptual information interfaces with conceptual knowledge^22,23^. Comparing model-derived representations to brain responses therefore provides a compact way to situate regions along an ordered computational hierarchy and to distinguish visually grounded features from more abstract semantic representations^24,25^.

Here, we investigated how information about visual scenes is encoded and routed across cortex by analyzing representational geometry shared among individuals and DNNs. We unified four complementary levels of analysis: shared representational structure across individuals, its layer-wise correspondence to vision and language models, the network topology implied by representational connectivity, and dimensionality decomposition relating these patterns to stimulus properties. These integrated analyses allowed us to move beyond prior studies focused primarily on local alignment or encoding performance, and instead identify a distributed organization, directly contrast vision and pure language model hierarchies across cortex, and interpret a representational-derived cortical connectivity graph using model-derived hierarchical ordering.

We applied this framework to the Natural Scenes Dataset (NSD)^26^, in which participants performed a memory recognition task while viewing thousands of natural scenes spanning diverse environments, objects, and social interactions. We recovered a cortex-wide organization with two dissociable processing routes, each specialized to represent distinct types of stimulus information. The medial-ventral stream was specialized for scene layout and environmental context, while the lateral stream was selectively tuned to animate and social content. DNNs captured the shared geometry of both routes, though with clear modality-specific differences: vision models aligned with both pathways, whereas language models corresponded primarily with the lateral stream and showed negative alignment in early visual and ventral cortex. These findings support refinements of classical visual-system accounts^3,27^ that include a ventromedial route for scene and contextual processing and a lateral route for social and biological information^28,29^. We then tested the generality of these findings in two independent datasets (BOLD5000^30^ and THINGS-fMRI^31^) and found that, although the same cortical areas consistently emerged in the alignment analysis, replicating the full two-route organization required rich scene and social content.

## Results

### Inter-subject shared geometry during scene viewing

**Fig. 1:**
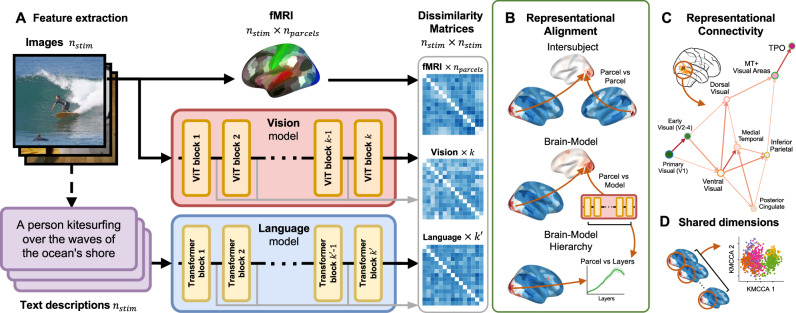
Overview of the analysis pipeline. **A** Feature extraction. For each image stimulus, we extracted corresponding representations from brain activity and deep neural networks. Single-trial fMRI responses were aggregated within cortical parcels to create vector representations of the brain’s response. Concurrently, layer-wise activations were extracted from pre-trained vision and language models to obtain representations across the full model hierarchies. Both brain and model vectors were used to compute representational dissimilarity matrices (RDMs). **B** Representational alignment. Representational Similarity Analysis (RSA) was used in two ways: (i) inter-subject RSA (IS-RSA), correlating parcel-wise RDMs across participants to estimate shared representational geometry; and (ii) brain-model RSA, correlating parcel RDMs with model-layer RDMs to quantify brain-model and layer-wise alignment profiles. **C** Representational connectivity. IS-RSA between pairs of parcels was used to construct a cortical network based on how similarly regions encode the stimulus set. The directionality of information flow was inferred from the peak model-layer alignment established in (**B**). **D** Shared dimensions. Within the main hubs identified in (**C**), Kernel Multi-view Canonical Correlation Analysis (KMCCA) was used to decompose the shared geometry into latent dimensions common across participants and to relate these dimensions to scene features that drive alignment in different parts of the network. Example image adapted from Wikimedia (Bengt Nyman, CC BY 2.0).

To localize cortical regions whose response patterns reflect reliable stimulus information in a shared format across people, we used inter-subject representational similarity analysis (IS-RSA)^10,11^. We applied this approach to the Natural Scenes Dataset (NSD)^26^, which provides high-resolution 7T fMRI responses from eight participants performing an inter-session long-term recognition memory task in which they detected repeated images while viewing thousands of natural scenes. Our goal was to measure the stimulus-driven representational geometry related to visual content (Fig. 1).

To isolate this geometry, we parcellated the cortical surface into 180 regions per hemisphere using the Human Connectome Project multimodal parcellation (HCP-MMP) atlas^32^ and extracted the provided single-trial responses for each image. For each subject and parcel, we constructed representational dissimilarity matrices (RDMs) from pairwise distances between multivoxel patterns. We then quantified inter-subject alignment by correlating parcel-wise RDMs across participants, using a shifted-repetition scheme to reduce mnemonic and session-related confounds (Supplementary Note 1). This yielded a parcel-wise map of shared representational geometry.

**Fig. 2:**
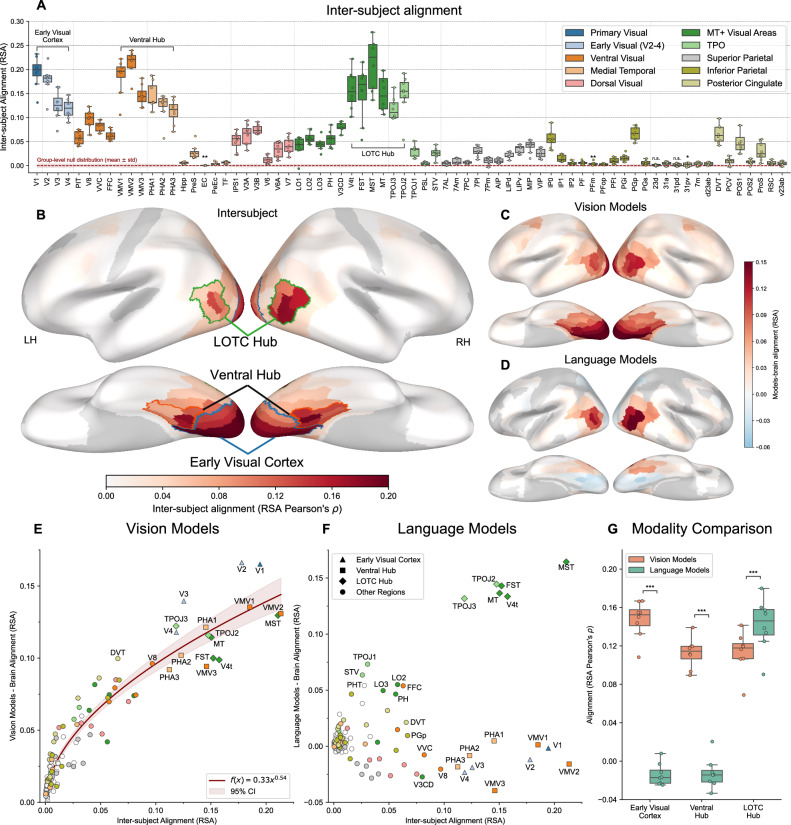
Inter-subject and model-brain alignment across cortex. **A** Inter-subject representational alignment (IS-RSA; Pearson’s *r* between parcel-wise RDMs across participants) for parcels drawn from the macro-anatomical clusters with the highest mean IS-RSA (color legend). Boxplots show the distribution across subjects in the NSD sample (*N* = 8, symmetric HCP-MMP atlas). Red lines indicate the parcel-wise null distribution (mean ± s.d.; 10,000 permutations). Unless otherwise annotated in the plot, parcels are significant at *** (*p* < 0.001; two-tailed, FDR-corrected); additional annotations indicate ** (*p* < 0.01), * (*p* < 0.05), or n.s. (*p *≥ 0.05). Peak alignment occurs in early visual cortex (V1-V4), a ventral hub (VMV1-3, PHA1-3), and a lateral occipitotemporal (LOTC) hub (V4t, MT, MST, FST, TPOJ2-3). Full-atlas results are shown in Supplementary Fig. S1. Cortical surface maps of alignment, averaged across subjects (and across models within each modality): (**B**) inter-subject IS-RSA; (**C**) vision-model RSA (maximum across layers, averaged across vision models); (**D**) language-model RSA (maximum across layers, averaged across language models). Parcel-wise relationship between IS-RSA and model-brain alignment for (**E**) vision models and (**F**) language models. Points are colored by macro-anatomical group (as in panel A); other parcels are shown in white. Vision parcels follow an approximate power-law fit (*R*^2^ = 0.94, shaded band: 95% bootstrap CI), whereas language-model alignment clusters near zero or negative except for parcels in and around the LOTC hub. **G** Modality comparison within the three hubs. Boxplots show hub alignment for vision and language models in early visual cortex, the ventral hub, and the LOTC hub (averaged across models). Paired two-tailed *t*-tests (*t*(7), FDR-corrected) indicate stronger vision-model alignment in early visual and ventral hubs, and stronger language-model alignment in the LOTC hub.

IS-RSA captured significant alignment across occipital cortex and extended into higher-order association cortex (Fig. 2a and Supplementary Figs. S1–S2). Alignment was strongest in early visual cortex (V1-V4) and extended into ventral occipitotemporal and dorsal occipitoparietal regions, consistent with hierarchical accounts of visual organization^2,27,33^. We also observed significant, albeit weaker, alignment in prefrontal regions, which may reflect shared contributions of visual attention and task-dependent semantic processing^34–36^. Within this map, three anatomically clustered sets of parcels showed especially strong and consistent alignment across participants (Fig. 2b).

The first cluster corresponds to the early visual cortex (V1-V4), consistent with its role in encoding low-level visual features^37^. The second comprised a ventral hub in ventromedial temporal cortex (VMV1-3, PHA1-3), overlapping scene-selective regions such as the parahippocampal place area (PPA) and adjacent cortex implicated in encoding scene layout and place memory^4,5,38^. The third comprised a lateral occipitotemporal cortex (LOTC) hub including the motion-selective MT+ complex and posterior temporoparietal junction (MT, MST, FST, V4t, TPOJ2-3), near regions responsive to bodies and biological motion that have been implicated in social perception^29,35,39^. In the following sections, we use these three hubs (early visual, ventral, and LOTC) as reference points for subsequent analyses.

This spatial distribution is consistent with prior NSD work reporting variability in signal reliability and decodability across cortex^26,40–42^. Across parcels, higher IS-RSA values were associated with greater signal stability under resampling (Supplementary Note 2) and were observed in regions with reliable stimulus-locked responses, indicating that response reliability is a prerequisite for detecting shared representational geometry, but not sufficient to explain the magnitude or spatial distribution of IS-RSA across cortex (Supplementary Fig. S3). Within-subject RSA and related variants produced a similar cortical pattern (Supplementary Fig. S4). Although alignment strength showed some hemispheric asymmetries (Fig. 2b), representational profiles of left-right homologous parcels were highly correlated, consistent with a largely bilateral organization (Supplementary Note 3). Together, these results indicate that IS-RSA isolates a distributed set of regions whose scene-evoked representational geometry is shared across individuals, providing a benchmark for testing how vision and language models differentially capture this shared geometry in the next sections.

### Model-brain alignment reveals modality-specific shared geometry

Although IS-RSA localized parcels whose representational geometry was shared across observers, it did not by itself specify which stimulus information gave rise to that shared structure. To distinguish visually grounded from more abstract features, we compared cortical RDMs with RDMs from pretrained vision and language models. Vision models provide hierarchies learned directly from pixels^43–45^, whereas large language models (LLMs) provide a reference for text-derived structure. By contrasting brain alignment with these two model classes, we characterized whether a parcel’s shared geometry was better captured by visually grounded features or by higher-level language representations.

We analyzed 31 pretrained models based on Vision Transformer (ViT)^46^ and Transformer^47^ architectures, spanning supervised, self-supervised, and contrastive objectives (Table 1). For each model, we extracted layer-wise activations for each NSD image (vision models) or its caption (language models), constructed layer-specific RDMs, and quantified model-brain alignment by correlating each layer RDM with each parcel RDM. For each model, parcel-wise alignment was summarized as the maximum RSA across layers. Fig. 2c, d show per-parcel maps averaged across subjects and, within each modality, across models (significance was assessed with a permutation-based null distribution).

**Table 1:** Vision and language models used in the analyses

Modality	Family	Training Regime	Model	# Params	# Blocks	Embedding size
Vision	ViT (AugReg)^80^	Supervised (21 K classes)	ViT (AugReg) Tiny	10M	12	192
			ViT (AugReg) Small	30M	12	384
			ViT (AugReg) Base	103M	12	768
			ViT (AugReg) Large	326M	24	1024
	CLIP (Vision)^81,82^	Contrastive Image-Text	CLIP (ViT) Base	86M	12	768
			CLIP (ViT) Large	304M	24	1024
			CLIP (ViT) Huge	632M	32	1280
		Contrastive I-T + fine-tuning (12 K)	CLIP (ViT) Base ft-12k	95M	12	768
			CLIP (ViT) Large ft-12k	315M	24	1024
			CLIP (ViT) Huge ft-12k	646 M	32	1280
	DINOv2^83^	Self-Supervised	DinoV2 (ViT) Small	22M	12	384
			DinoV2 (ViT) Base	87M	12	768
			DinoV2 (ViT) Large	304M	24	1024
			DinoV2 (ViT) Giant	1.1B	40	1536
	MAE^84^	Self-Supervised (Masked AE)	MAE (ViT) Base	86M	12	768
			MAE (ViT) Large	303M	24	1024
			MAE (ViT) Huge	631M	32	1280
Language	BLOOMZ^85^	Causal LM + Instruction FT	BloomZ (560M)	559M	25	1024
			BloomZ (1b1)	1.1B	25	1536
			BloomZ (1b7)	1.7B	25	2048
			BloomZ (3B)	3B	31	2560
			BloomZ (7b1)	7.1B	31	4096
	Gemma 2^86^	Causal LM	Gemma 2 (2B)	2.6B	27	2304
			Gemma 2 (9B)	9.2B	43	3584
	LLaMA^87,88^	Causal LM	OpenLlama (3B)	3.4B	27	3200
			OpenLlama (7B)	6.7B	33	4096
			OpenLlama (13B)	13B	41	5120
			HuggyLLaMa (7B)	6.7B	33	4096
			HuggyLLaMa (13B)	13B	41	5120
	LLaMA 3^89^	Causal LM	Llama 3 (8B)	8B	33	4096
			Llama 3.1 (8B)	8B	33	4096

Vision models produced an alignment map that closely matched the IS-RSA spatial pattern (Figs. 2c vs. 2b). Parcels with high IS-RSA also showed strong vision-model alignment, with peaks in the Early Visual, Ventral, and LOTC hubs and weaker but significant effects across additional visual and prefrontal parcels. This overlap indicates that image-trained feature spaces capture a substantial component of the stimulus-structured geometry that is shared across individuals during scene viewing, consistent with prior NSD results and ventral-stream model-brain alignment work^18–20,23,26,40^. Across parcels, IS-RSA and vision-model alignment were strongly related by approximate power-law scaling (*R*^2^ = 0.94; Fig. 2e), consistent with both measures reflecting a common parcel-wise organization expressed with different attenuation profiles and scales (see Supplementary Note 4 for analysis of scaling and reliability).

In contrast to the broad cortical correspondence observed for vision models, autoregressive LLMs trained on next-token prediction showed alignment concentrated in LOTC, with weaker effects extending into adjacent posterior temporal regions around the superior temporal sulcus and temporo-parieto-occipital junction, as well as ventral occipitotemporal cortex (Fig. 2d, f), regions implicated in biological motion, social perception, and multimodal processing^22,29,35,39^. Alignment in these areas is consistent with prior reports of language-model correspondence in lateral/anterior temporal cortex and medial frontal regions in naturalistic paradigms associated with semantic processing^22,23,42^. Within the LOTC hub, language-model alignment exceeded vision-model alignment (Fig. 2g), indicating that the shared geometry expressed in this hub is better captured by caption-derived structure than by purely visual model features.

Conversely, early and ventral visual regions showed near-zero or negative RSA with these LLM embeddings (Fig. 2d and Supplementary Figs. S1–S2). Negative RSA indicates a systematic mismatch in geometry: stimulus features that separate responses in these parcels, containing visual characteristics, are not emphasized by the pure language-model representations. This negative alignment contrasts with studies reporting positive ventral correspondence for the output layers of language models optimized for sentence similarity or language–vision contrastive objectives^23,42^. We propose that this difference may arise from the training objective: unlike the next-token prediction models used here, similarity-based models are optimized to group semantically similar scene descriptions in their output space, thereby producing geometries that overlap more strongly with high-level ventral visual representations. By contrast, the autoregressive models studied here appear to emphasize other dominant semantic dimensions, yielding alignment concentrated in LOTC rather than along the ventral visual pathway.

### Hierarchical correspondence between models and cortex

**Fig. 3:**
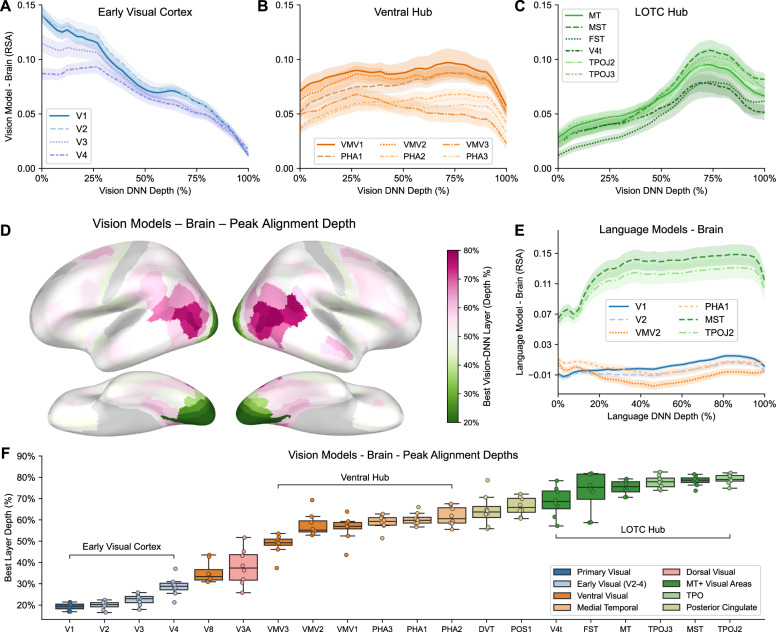
Hierarchical correspondence between model layers and cortical regions. **A**–**C** Layer-wise alignment (RSA) between vision models and representative parcels in the three hubs, averaged across all vision models. Panels show early visual cortex (**A**), the Ventral hub (**B**), and the LOTC hub (**C**). The *x*-axis expresses depth as a percentage of the total number of layers in each vision model. Curves show the mean alignment across participants (*N* = 8); shaded bands indicate the standard error of the mean (SEM). Early visual parcels peak in shallow layers, Ventral parcels show distributed alignment across the hierarchy, and LOTC parcels peak in the deepest layers. **D** Cortical surface map of the vision-model layer that yields the highest alignment for each parcel (averaged across vision models). Colors encode normalized peak-layer depth (0% = shallowest layer, 100% = deepest layer), revealing a posterior-anterior gradient from shallow (green) to deep (purple) alignment. **E** Layer-wise alignment between language models and representative parcels from the three hubs (mean across language models). Alignment values are negligible in early visual and Ventral parcels and are restricted to LOTC, where curves rise quickly and then form a plateau rather than a smooth hierarchical progression. **F** Distribution across participants (*N* = 8) of peak alignment depths for the 20 parcels with the highest overall vision-model alignment. For each subject and parcel, peak depth is defined as the mean depth (across vision models) of the layer at which RSA is maximal. Boxplots are ordered by median peak depth and colored by macro-anatomical cluster.

To relate cortical parcels to the ordered stages of representation learned by DNNs, we examined how model-brain alignment varied across model depth. Prior work has shown that, for vision models, shallow layers tend to align with early visual cortex and deeper layers tend to align with higher-level ventral and downstream regions^14,18–20,48^. Here, we assessed this depth dependence during natural scene viewing in NSD by analyzing layer-wise RSA curves, focusing on the three hubs where inter-subject shared geometry was strongest (Fig. 3; full parcel-wise curves in Supplementary Fig. S5).

The three hubs showed distinct depth profiles for vision models (Fig. 3a-c). Early visual parcels peaked in the shallowest layers and then decreased (Fig. 3a), consistent with correspondence to lower-level visual features. Ventral hub parcels showed broad alignment spanning earlier and later layers (Fig. 3b), indicating that their shared geometry relates to features distributed across multiple stages of the vision-model hierarchy rather than a single best-matching depth. LOTC parcels increased more steadily with depth and reached their maxima in deeper layers (Fig. 3c), indicating that the stimulus organization expressed in LOTC is best matched by later-stage vision-model features.

Across vision-model families (Table 1), these depth-dependent trends were broadly similar (Supplementary Fig. S6). Early visual alignment generally decreased with depth, LOTC alignment increased toward later layers, and ventral alignment remained more stable or showed a broader peak across intermediate-to-late layers. The clearest family-level exception was the Masked Autoencoder (MAE) family, which retained relatively strong alignment with early visual cortex even in deeper layers, consistent with its image-reconstruction objective preserving low-level visual structure more strongly than the other model families. By contrast, CLIP-family models did not show a qualitatively distinct overall RSA profile from the other vision-model families, although their later layers also aligned strongly with LOTC.

To summarize this organization across cortex, we identified for each parcel the vision-model layer with maximal alignment and projected this peak depth onto the cortical surface. The resulting map formed a posterior-to-anterior gradient in which early visual cortex was best explained by shallower layers, while more anterior occipitotemporal, STS-adjacent, and prefrontal regions were best explained by deeper layers (Fig. 3d). This gradient is consistent with hierarchical accounts of visual processing^2,5,27,33^ and with prior work showing that model depth tracks representational complexity across the human ventral pathway^14,18–20,45,48^. We use peak depth as a descriptive index that orders parcels along the model hierarchy (Fig. 3f).

Language models showed a different pattern from vision-model alignment (Fig. 3e), revealing a narrower and qualitatively distinct profile across cortex. Alignment was concentrated in the LOTC hub, and layer-wise profiles did not show the early-to-late progression observed for vision models. Instead, alignment was present at the first layer, increased, and then approached a plateau. Different language-model families likewise showed qualitatively similar alignment profiles (Supplementary Fig. S6). Across the language models we evaluated, first-layer alignment strongly predicted the plateau magnitude (Pearson’s *r* = 0.97) and was related to the tokenizer representation (Pearson’s *r* = 0.78; Supplementary Note 5). Together, these results indicate that much of the observed brain–language alignment in the present visual-caption setting is predicted by dominant shallow lexical-semantic content, closer to a bag-of-concepts organization than to progressively composed syntax, consistent with prior work showing that relatively simple semantic similarity spaces can capture structure in high-level visual cortex^49,50^. We propose that the difference in layer-wise alignment profiles between vision and language models reflects, a mismatch in the form of their inputs: vision models and cortex are compared while transforming pixel-level images, whereas language models operate on tokenized captions that already provide an abstracted description. Under this interpretation, language-model alignment emerge in regions that express higher-level scene organization, such as LOTC, without producing a depth-dependent gradient across the visual hierarchy.

### A representational network linking ventromedial and lateral hubs

**Fig. 4:**
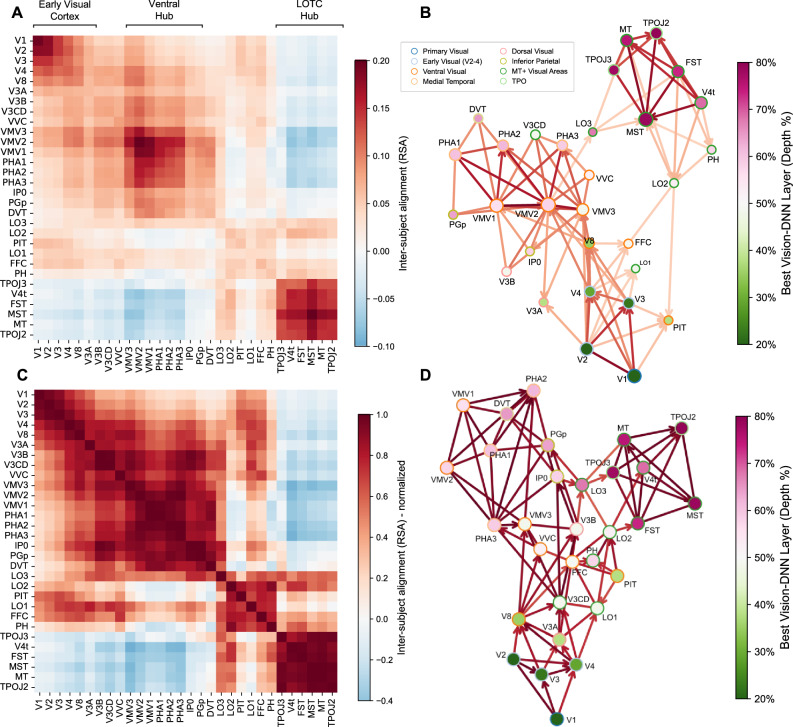
Inter-subject representational connectivity network. **A** *Inter-subject connectivity matrix*. Parcel-wise representational connectivity (RSA; Pearson’s *r*) for the 30 parcels with the highest mean IS-RSA (*N* = 8). Each cell shows the mean RSA between the RDMs of two parcels, computed across all cross-subject pairs. The matrix shows three clusters corresponding to early visual cortex (V1–V4), the Ventral hub (VMV1-3, PHA1-3), and the LOTC hub (V4t, MT, MST, FST, TPOJ2-3). Warm colors indicate higher representational similarity; cool colors indicate dissimilarity. **B** *Directed connectivity graph*. The same parcels are shown as nodes. Node color encodes each parcel’s peak alignment depth with vision models (heuristic proxy for hierarchical position), node size indicates inter-subject alignment strength, and node border color indicates macro-anatomical group membership. Edges correspond to the union of three minimum spanning trees; edge color reflects the RSA value in (**A**), and edge width indicates the spanning-tree iteration (first to third). Arrow direction is assigned from shallower to deeper peak-depth parcels when the depth difference is significant across subjects (paired *t*-test, *t*(7), FDR-corrected *p* < 0.05). The graph highlights two dominant routes from early visual cortex: a medial-ventral route toward the Ventral hub and a lateral route toward the LOTC hub. **C** *Within-ROI-normalized inter-subject connectivity matrix*. Pairwise inter-parcel connectivity values were normalized by the corresponding within-ROI alignment terms to control for differences in overall alignment magnitude across parcels. The normalized matrix preserves the same broad three-cluster organization observed in the unnormalized analysis. **D** *Directed connectivity graph derived from the normalized inter-subject connectivity matrix*. Using the same graph-construction procedure, the normalized analysis recovers a dual-stream topology, with only minor differences in specific ROI-to-ROI connections.

To characterize how regions with shared scene-evoked information relate to each other, we employed IS-RSA as a measure of representational connectivity. This defines a task- and stimulus-dependent network based on similarity in how stimuli are organized across regions, rather than on anatomical proximity or raw BOLD covariance. For each pair of parcels, we correlated their RDMs across different individuals, yielding a parcel-by-parcel connectivity matrix that quantifies how similarly distinct regions organize the stimulus set across observers (Fig. 4a). This approach emphasizes the informational content of the connectivity by grouping regions that share a common representational format, complementing univariate and conventional functional-connectivity analyses^51,52^.

The connectivity matrix showed three clusters aligned with the Early Visual, Ventral, and LOTC hubs (Fig. 4a). Early visual and ventral parcels formed a broad posterior cluster that also included occipital and parietal cortex, indicating similar stimulus organization across these regions. LOTC parcels formed a second cluster with low or negative similarity to the posterior cluster. The strongest links between these systems passed through intermediate parcels in lateral occipital and ventral temporal cortex (LO1–3, FFC, PH). Because IS-RSA reflects both shared structure and overall alignment magnitude within each parcel, differences in within-parcel alignment can influence edge strength. To control for this, we also computed a normalized version of the connectivity matrix in which each pairwise parcel-to-parcel value was scaled by the corresponding within-parcel alignment terms, using the within-ROI IS-RSA value as a normalization factor (Fig. 4c). This normalization preserved the same broad three-cluster organization and recovered a dual-stream network topology (Fig. 4d), with minor differences in specific ROI-to-ROI connections. A qualitatively similar two-stream organization was also reproduced in a within-subject analysis (Supplementary Fig. S4), in which representations from different repetitions of the same images were compared within each participant.

To visualize the strongest routes, we extracted a sparse backbone graph by iteratively applying a minimum spanning tree algorithm to the parcel-wise connectivity matrix. We then oriented edges using each parcel’s peak alignment depth from the vision-model analysis (Fig. 3d, f) as a descriptive proxy for representational level, from shallower-layer peaks toward deeper-layer peaks. This ordering is not anatomical and does not imply causal direction. By combining an IS-RSA-derived cortical graph with model-derived hierarchical ordering, this analysis links representational connectivity to model hierarchy within a single framework, allowing shared stimulus geometry to be interpreted as an ordered cortical network. In the resulting graph, early visual cortex bifurcated into two routes (Fig. 4b, d): a medial-ventral path progressing through ventral occipitotemporal cortex into the Ventral hub, and a lateral path progressing through lateral occipital cortex into the LOTC hub. Extending the analysis to the whole brain (Supplementary Fig. S7) showed both routes continuing into association cortex, consistent with these representational formats interfacing with downstream systems implicated in memory, action, and control^53,54^.

This two-route organization does not map cleanly onto the classical “ventral what/dorsal where” framing of visual processing^3,27,33,55^. The medial-ventral branch targeted parahippocampal and ventromedial visual parcels (including VMV and parahippocampal scene regions) that are consistently linked to scene layout and environmental context^4,56,57^, and it matches proposals of a ventromedial, scene-based pathway that provides scene input to hippocampal memory systems^5,28^. The lateral route, by contrast, encompassed MT+ and LOTC and continued toward posterior STS and temporo-parietal cortex in the whole-brain graph, regions implicated in dynamic cues, biological motion, body perception, and social processing^28,29,39,58,59^. In this representational network, regions typically associated with the classical dorsal stream in intraparietal and superior parietal cortex were less prominent in shared geometry, but they remained part of the broader posterior cluster. This pattern suggests that the LOTC-centered trajectory reflects a distinct lateral pathway whose shared stimulus organization is driven most strongly by biological and socially relevant content, rather than the spatial computations emphasized in traditional “where/how” accounts^29^.

### Representational dimensions driving shared geometry

The two-route organization of the scene network raises a concrete question: which stimulus information most strongly organizes the shared representational geometry within each hub and along each route. To address this directly from the data, under the present task context, we decomposed shared across-subject structure in the Early Visual, Ventral, and LOTC hubs using Kernel Multi-view Canonical Correlation Analysis (KMCCA)^60^. Unlike RSA, which summarizes similarity between two RDMs with a single correlation, KMCCA treats each participant’s hub RDM as a separate view of the same stimulus set and learns components that maximize correlation across views. We applied multi-view CCA ideas^61^ to RDM-derived kernels, yielding a low-dimensional set of components that captures the geometry shared across participants within each hub.

**Fig. 5:**
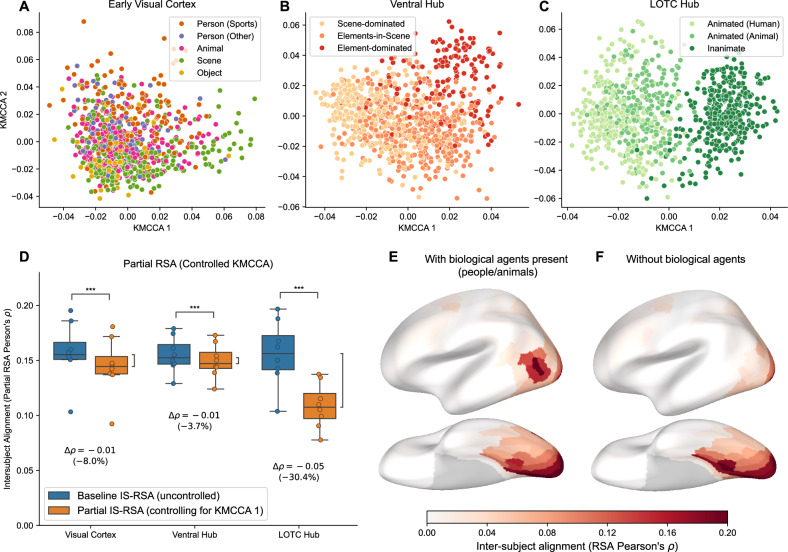
KMCCA components and biological-content dependence of LOTC shared geometry. **A**–**C** Stimulus projections onto the first two KMCCA components for each hub, estimated from all eight NSD participants using the shared image set. Early Visual Cortex (**A**) shows no clear clustering by scene category. The Ventral hub (**B**) varies along a scene-object continuum. The LOTC hub (**C**) shows a categorical separation along an animacy-related axis (animate agents vs. non-animate scenes/objects). **D** Effect of removing the first KMCCA component (KMCCA1) using partial RSA. Orange boxplots show partial IS-RSA after regressing out KMCCA1 from the hub RDMs; blue shows the original IS-RSA. Removing this single dimension reduces inter-subject alignment in all hubs, with the largest reduction in LOTC. IS-RSA was computed per parcel and subject, averaged within hub, and then averaged across subjects; paired *t*-tests were Bonferroni-corrected (*t*(7), *p* < 0.001). Annotated *Δρ* reports the mean absolute change, and percentages denote the relative change normalized by baseline. Inter-subject RSA maps recomputed after splitting the stimulus set into scenes *with* biological agents (**E**) versus *without* biological agents (**F**). LOTC alignment is strongly reduced when biological content is absent.

To visualize this shared geometry, we projected stimuli onto the first two KMCCA components for each hub (Fig. 5a–c). In early visual cortex, the projection showed a diffuse distribution with no clear clustering by scene category, consistent with representations dominated by low-level image structure under this stimulus set and task^42^ (Fig. 5a). In the Ventral hub, the leading axis formed a continuous gradient from panoramic scenes to more object-like images, consistent with ventromedial scene-object organization and scene layout/context representations^4,5,28,56,57^ (Fig. 5b). In the LOTC hub, the first component separated images containing biological agents (people/animals) from non-biological images, consistent with LOTC sensitivity to biological and social content^29,35,39^ (Fig. 5c).

We then quantified how much this leading axis contributed to each hub’s shared geometry. We regressed out the first KMCCA component and recomputed inter-subject alignment using partial RSA (Fig. 5d). Removing this single axis reduced IS-RSA in all three hubs (all *p* < 0.001, Bonferroni-corrected), but the reduction was substantially larger in LOTC (−30.4%) than in Early Visual Cortex (−8.0%; *t*(7) = −10.78, *p* < 0.001) or the Ventral hub (−3.7%; *t*(7) = −12.21, *p* < 0.001). This pattern indicates that an animacy-related dimension accounts for a large fraction of LOTC’s shared geometry, whereas Early Visual and Ventral hubs distribute shared variance across higher-dimensional structure (Supplementary Note 7).

Given the dominant agent-related axis in LOTC, we tested whether lateral-route alignment depended on biological content by splitting the stimulus set into scenes with biological agents (people or animals; 65.3% of images) versus scenes without them, and recomputing IS-RSA within each subset. Alignment in LOTC was strongly reduced for non-biological scenes but remained robust when biological agents were present (Fig. 5e-f). The same split also weakened the lateral branch of the representational network linking early visual cortex to LOTC, reducing the detectability of the two-route backbone when biological content was absent (Supplementary Fig. S8). This pattern indicates that biological-agent content is a major contributor to LOTC shared geometry in this stimulus set and task regime.

### Comparison across tasks and stimulus sets

**Fig. 6:**
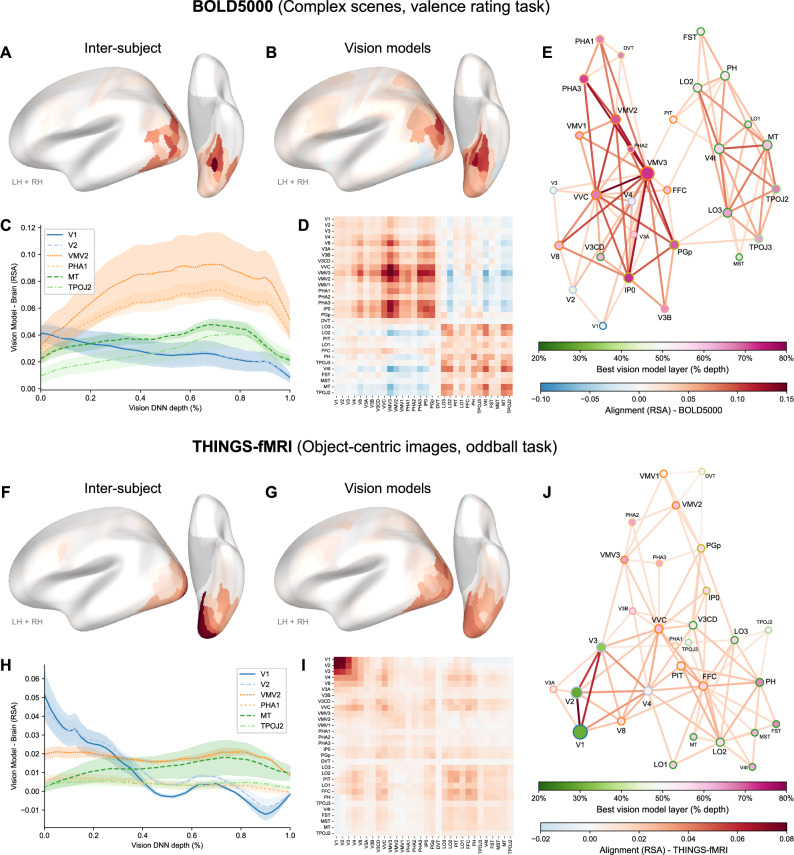
Comparison across independent datasets. Analyses were performed on the BOLD5000 and THINGS-fMRI datasets using the same pipeline as NSD. **A**–**E** *BOLD5000 (complex scenes, valence task*; *N *= 4). Despite lower signal reliability and differences in task, the core network topology persists in this dataset, which shares similar scene statistics with NSD. Inter-subject alignment (**A**) and vision-model alignment (**B**) recover the characteristic occipitotemporal footprint, including Ventral and LOTC hubs, though with reduced magnitude and spatial continuity compared to NSD. **C** Layer-wise alignment profiles (mean  ± SEM across subjects) preserve the hierarchical ordering (Early Visual  < Ventral  < LOTC) but exhibit flatter curves. Representational connectivity matrix (**D**) and backbone graph (**E**) successfully recover the dual-stream topology (based on a *k* = 3 minimum spanning tree), segregating the medial--ventral route from the lateral route. In the graph (**E**), node fill indicates peak model depth and edge color indicates representational connectivity strength. **F**–**J** *THINGS-fMRI (object-centric images, oddball task*; *N* = 3). With minimal scene layout and social content, the network contracts. Inter-subject (**F**) and vision-model (**G**) alignment are restricted primarily to early and ventral visual cortex, with no prominent LOTC peak. **H** Layer-wise profiles (mean  ± SEM) are reduced in magnitude and show flatter profiles, especially in ventral and LOTC hubs. The lateral stream does not emerge as a distinct subsystem in the connectivity matrix (**I**), and the backbone graph (**J**) lacks the clear two-route segregation evident in NSD and BOLD5000, consistent with the content-dependence of the lateral pathway.

Our analyses in NSD identified a distributed scene network by quantifying representational geometry shared across individuals and relating it to model feature spaces. Because this geometry is stimulus- and task-dependent, its spatial expression can vary with the regularities present in the stimulus set^62,63^, task demands that reweight the stimulus features that are emphasized^36,64^, and measurement reliability, which bounds how well shared structure can be estimated from trial-level data^65–67^. To assess how the inferred organization generalizes across experimental regimes, we applied the same pipeline to two independent datasets: BOLD5000^30^ and THINGS-fMRI^31^ (Fig. 6).

BOLD5000^30^ uses natural images broadly similar to NSD but differs in task (valence judgment vs. memory), stimulus duration (1 s vs. 3 s), and trial-level reliability under comparable estimation procedures^26^. In this regime, inter-subject and vision-model alignment remained concentrated in occipital and occipitotemporal cortex, with clear involvement of the ventral and LOTC hubs identified in NSD (Fig. 6a, b), consistent with both datasets sampling rich scene layout, context, and social content. However, the overall spatial profile differed from NSD in two main respects. Early visual cortex was not detected as a dominant peak, and the LOTC pattern was less spatially contiguous. These differences are likely attributable to reduced signal reliability resulting from the lower field strength (3T) and shorter presentation times. Despite these differences, vision-model alignment preserved a monotonic parcel-wise relationship with IS-RSA, as in NSD (power-law scaling *R*^2^ = 0.71). Layer-wise profiles preserved the hub-specific ordering but were flatter than in NSD (Fig. 6c).

At the network level, representational connectivity in BOLD5000 still separated a broad posterior cluster spanning early visual, ventral, and adjacent cortex from a lateral occipitotemporal cluster that included LOTC together with nearby lateral occipital and ventral temporal parcels (Fig. 6d). LOTC was less segregated from neighboring lateral parcels than in NSD, but the backbone visualization still recovered a two-route organization from early visual cortex toward ventral versus lateral targets (Fig. 6e), consistent with a ventromedial route associated with scene context and a lateral route associated with biological and social features.

THINGS-fMRI differs from NSD in both stimulus content and task. It uses briefly presented (500 ms), object-centric images with minimal scene layout and reduced social content, and participants performed an orthogonal oddball task^31^. In this regime, inter-subject alignment and vision-model alignment again remained tightly coupled (power-law scaling *R*^2^ = 0.80; Fig. 6f, g), but the spatial pattern was concentrated in occipital and ventral visual cortex. The NSD ventromedial/parahippocampal and LOTC hubs were not detected as prominent hubs, and LOTC resembled the NSD pattern obtained when restricting analyses to images without biological agents (Fig. 5f). Layer-wise profiles retained the same ordering but were reduced in magnitude and less distinctive for the ventral and LOTC hubs (Fig. 6h). Consistent with this, representational connectivity did not separate an LOTC-centered subsystem (Fig. 6i), and the backbone graph did not recover the two-route topology observed for NSD and BOLD5000 (Fig. 6j).

Across datasets, vision-model alignment continued to track inter-subject alignment, indicating that the same stimulus-locked geometry that is detectable across observers is also present in image-trained feature spaces. However, which hubs and routes emerged as prominent depended on the structure available in the stimulus set and on task/reliability constraints: scene-rich datasets expressed ventromedial/parahippocampal and LOTC involvement more clearly, whereas the object-centric THINGS regime emphasized early and ventral visual cortex and did not express a separable LOTC-centered route.

## Discussion

In this study, we aimed to characterize how the human cortex encodes natural scenes by quantifying representational structure shared across individuals and comparing it to hierarchical features in DNNs. We identified a distributed network of cortical regions engaged by scene perception and characterized its major hubs and the stimulus features that organize the processing within it.

### Two representational routes during scene viewing

We identified this distributed network engaged during scene perception using inter-subject representational connectivity, which captures similarity in how different regions organize the stimulus set. The resulting topology separated three prominent hubs connected by two dominant routes: an early visual hub, a ventral hub spanning ventromedial and parahippocampal scene regions, and an MT+/LOTC hub. The early visual hub bifurcated into branches toward the ventral and lateral hubs, and the ventral and lateral systems were additionally coupled via intermediate lateral occipital and ventral temporal parcels (Fig. 4). This pattern therefore does not conform to the classical ventral “what” versus dorsal “where/how” partition^3,27^, as it did not recover the canonical occipito-parietal dorsal route as a dominant branch of the representational network. Instead, the dominant topology was organized around ventromedial and lateral occipitotemporal pathways. These identified dominant routes are compatible with a ventromedial pathway related to scene and contextual structure, and a lateral pathway centered on MT+ and STS-adjacent regions associated with social and biological information^28,29,68^. However, inter-subject synchrony studies using naturalistic movie paradigms have reported strong cross-subject alignment in dorsal occipito-parietal regions^6^. The weaker alignment of the dorsal stream in the present analyses may reflect both the use of static scene images and the fact that representational connectivity, based on single-trial beta patterns, emphasizes shared stimulus geometry and image content rather than temporal synchrony, action-related processing, or broader task-dependent aspects of neural activity.

The medial-ventral route aligns with accounts of a ventromedial “where/scene” pathway that supports environmental layout and spatial context, providing inputs to hippocampal circuitry^5,28,69^. In our data, parcels in this ventral hub showed broad correspondence to vision models across multiple depths (Fig. 3b), indicating that their shared geometry relates to features distributed across multiple stages of the vision-model hierarchy. This pattern held for scenes with and without biological agents (Fig. 5e, f). It was reduced in the object-centric THINGS dataset, which contains limited scene layout and context (Fig. 6e, f), confirming that this route is most detectable when the stimulus set contains rich environmental information. Language-model alignment in these regions showed no correspondence (Fig. 2d), diverging from findings using similarity-based models^23,42^, indicating that caption-derived similarity structure did not capture the visually grounded scene-layout geometry expressed along the medial-ventral route.

The lateral route aligns with accounts of an MT+/STS-centered pathway specialized for socially relevant information, including “third visual pathway” and dorsolateral subdivision proposals^28,29,68^. Here, shared geometry was dominated by biological-agent content. The leading LOTC dimension separated scenes with animate agents from those without, and restricting analyses to images without biological agents reduced LOTC inter-subject alignment and weakened the lateral branch of the network (Fig. 5e, f and Supplementary Fig. S8). LOTC parcels aligned most strongly with later stages of vision models (Fig. 3c) and also aligned with language-model representations (Fig. 2d, f, g), consistent with this route emphasizing socially structured distinctions that are expressed in deep visual features and are also present in text-derived semantic structure.

### Model-brain alignment across the visual hierarchy

Model-brain RSA related the representational geometry measured in cortex to the ordered progression of features across model depth. For vision models, model-brain correspondence tracked the inter-subject shared geometry across parcels and scaled with IS-RSA with an approximate power-law relationship (Fig. 2e). This suggests that the geometry shared across observers during scene viewing is also present in image-trained feature spaces, consistent with the idea that brain-model alignment may be driven in part by a set of shared organizing dimensions in visual tasks^17^.

Layer-wise profiles revealed a parallel with the posterior-to-anterior ordering expected from the visual hierarchy (Fig. 3)^2,27,33^, but the mapping was not one-to-one. For example, while the ventral hub showed correspondence across multiple depths, alignment in the MT+/LOTC hub appeared to be disproportionately driven by a subset of stimuli containing animate content (Fig. 5). This is consistent with cortical representations drawing on features distributed across multiple stages and being modulated by stimulus content, rather than following a strictly feedforward depth-to-stage mapping of models.

Language models exhibited a distinct profile, reflecting the nature of their input. Because these models processed tokenized captions–abstracted from pixel data–they did not follow the cortical depth gradient observed in vision models. Instead, alignment was concentrated in LOTC and adjacent temporal regions, driven primarily by biological-agent content, while remaining weak or negative in early visual and ventral areas. Layer-wise alignment rose early and plateaued (Fig. 3e), scaling with tokenizer alignment (Supplementary Note 5). We interpret this pattern as indicating that the correspondence between language models and LOTC is driven largely by shallow lexical-semantic content–closer to a bag-of-concepts organization than to progressively composed syntax–consistent with earlier observations that relatively simple semantic similarity spaces can capture structure in high-level visual cortex^49,50^. In this respect, the contrast between autoregressive language models and vision models indicates that the language-model correspondence observed here is narrower in anatomical extent and qualitatively different in structure from the broader hierarchical alignment expressed by vision models.

More generally, these findings align with observations that modern models tend to converge on similar latent representations across objectives and architectures as they scale^17,70,71^. These findings reveal common representational axes across otherwise very different systems, consistent with the possibility that shared stimulus statistics constrain their emergence, although the mechanisms behind this convergence remain unresolved.

### Reliability and interpreting RSA magnitudes

Across analyses, inter-subject, vision-model, and alternative RSA alignment variants were related by approximate power-law scaling (Fig. 2e and Supplementary Note 4). These nonlinear relations indicate that the different RSA measures are not indexing unrelated properties, but rather a common parcel-wise organization expressed through comparisons with different noise levels, attenuation profiles, and degrees of shared representational variance captured by each comparison. In this sense, the empirical relations between measures suggest that RSA magnitudes depend not only on the amount of shared structure present but also on how strongly each comparison attenuates parcels at different alignment levels.

This has an important consequence for interpreting model–brain correspondence. Because IS-RSA and vision-model RSA are related nonlinearly, brain–brain alignment is not empirically expressed as a direct numeric ceiling for model alignment. In particular, vision-model RSA can show weaker nonlinear attenuation and even exceed IS-RSA in low-alignment parcels, while remaining lower in overall magnitude. This pattern suggests that the different RSA measures provide complementary views of a common underlying organization, rather than simple upper and lower bounds of the same quantity, and motivates a more specific analysis of which dimensions of visual representation are captured by each metric^65–67^.

More generally, the reliability of stimulus-locked signal constrains whether shared geometry can be detected, but does not by itself explain the cortical distribution of the observed alignment (Supplementary Fig. S3). In our control analyses, temporal signal-to-noise ratio (tSNR) was not significantly associated with IS-RSA, whereas noise-ceiling SNR (NCSNR)^26^ showed a broader positive association across cortex. However, among the highest-alignment parcels, reliability and IS-RSA were only weakly correlated. Together with the recovery of dissociable ventral and lateral routes, each associated with different stimulus dimensions, this indicates that the observed topology cannot be reduced to regional variation in signal quality alone. Reliability sets the conditions under which shared representational structure can be observed, but the topology that emerges reflects the stimulus information represented in each region rather than signal quality alone.

### Conclusions and scope

In this work, we characterized a cortical network engaged during scene viewing at the level of representational geometry. By integrating complementary analyses of alignment, representational connectivity, and dimensionality decomposition, we linked three questions often treated separately: where shared stimulus information is expressed, how that information is routed across regions, and which stimulus properties organize representations within different network hubs. Together, these analyses provide a comprehensive perspective on how scene information is structured across the human cortex.

This representational approach offers a general and adaptable methodology for identifying task-engaged networks and comparing information transformation across diverse systems. However, this flexibility entails a tradeoff: inferences concern representational organization rather than the precise mechanisms that implement it^72,73^. Because these comparisons rely on global similarity structure, computational dimensions that are subtle but behaviorally relevant may contribute weakly to the measured geometry, or be missed entirely if not captured by the chosen metric or feature sampling^74^. Consequently, these results should be interpreted as context-dependent–varying with stimulus sampling, task demands, and measurement reliability^75^–motivating future work to causally isolate the specific stimulus factors driving these alignments.

Despite these limitations, this framework provides a practical route for leveraging large-scale data to move beyond simply asking whether two systems encode information similarly. By decomposing shared geometry to isolate driving features, the approach helps identify candidate factors and interactions that explain *what* information is shared across cortex and *how* it is distributed. As dataset scale and reliability increase, these tools can bridge the gap between network-level descriptions and experiments probing how distributed cortical systems support perception and flexible behavior in dynamic environments.

## Methods

### Functional-MRI data

For the main analyses, we used the Natural Scenes Dataset (NSD)^26^, a high-resolution 7T fMRI dataset with 8 participants performing an image recognition-memory task. On each trial, participants viewed a natural scene for 3s and reported whether it was old or new. Each participant completed 32–40 scanning sessions (750 trials per session), yielding up to ~30,000 trials per participant with repeated presentations of images across sessions. Full acquisition and experimental details are reported in Allen et al.^26^.

Analyses were based on the 1.0-mm volumetric preprocessing of the NSD dataset and the corresponding single-trial *β*-estimates (version 3)^26^. We used GLM-denoised single-trial *β*-estimates^76^, as their image-related information content has been validated in multiple decoding and image reconstruction studies^40,77,78^. Voxels were grouped into cortical parcels using the HCP-MMP1.0 atlas^32^, which defines 180 cortical regions per hemisphere. We employed the pre-aligned parcel masks provided by the NSD authors to extract voxel activity within each cortical region^26^. Analyses were performed using both the symmetric version of the atlas (combining hemispheres) and the asymmetric version (preserving hemisphere separation); a supplementary analysis confirms that symmetric parcels display highly correlated representational profiles (Supplementary Note 3). In all figures, parcels are colored by macro-anatomical groups as defined in Huang et al. (2022)^79^, to facilitate anatomical interpretation.

For each participant $p\,\in \,{{\mathcal{P}}}$, parcel $r\,\in \,{{\mathcal{R}}}$, and session $s\,\in \,{{\mathcal{T}}_p}$, we define a response matrix ${X}_{p,r,s}\,\in \,{{\mathbb{R}}}^{n\times {v}_{p,r}}$ containing the voxel responses to each stimulus in that session. Here, *n* denotes the number of trials (750 per session), and *v*_*p*,*r*_ the number of voxels in parcel *r* for participant *p*.

Comparative analyses were performed using fMRI data from the BOLD5000^30^, and THINGS^31^ datasets. We selected these datasets because IS-RSA and KMCCA require a large number of matched stimuli across participants, which is often limited in larger-cohort naturalistic datasets. Preprocessing and analysis followed the same procedure described above, with minor adaptations to accommodate differences in stimulus sampling and repetition structure. Details are provided in Supplementary Note 8.

All data analysed are fully de-identified and publicly available. The original NSD collection was approved by the University of Minnesota Institutional Review Board (Allen et al.^26^). The THINGS dataset was collected under NIH Institutional Review Board approval (Hebart et al.^31^), and BOLD5000 under the Institutional Review Board of Carnegie Mellon University (Chang et al.^30^). No new human-subjects data were acquired for this work.

### Model features extraction

To compare how deep learning models encode visual and linguistic stimuli relative to fMRI responses, we selected vision and language models previously used by Huh et al. (2024) in studies of representational convergence across modalities^70^. Model selection was conducted prior to analysis, balancing computational constraints and the goal of covering diverse training objectives and model scales. A full list of models is provided in Table 1.

We selected 17 vision models spanning families with distinct training regimes, including ViT AugReg^80^, CLIP^81,82^, DINOv2^83^, and MAE^84^. All architectures were based on sequential Vision Transformer (ViT) blocks^46^. Each NSD image was processed through the backbone of each model, and activations were extracted from the output of each ViT block (see Fig. 1A).

For language models, we selected 14 transformer-based encoders from the BLOOMZ^85^, Gemma 2^86^, LLaMA^87,88^, and LLaMA 3^89^ families^47^. For each NSD image, we used text captions annotated by trained raters and originally sourced from the MS-COCO dataset^90^. Captions were processed through each model’s encoder, and activations were extracted at the output of each transformer block.

Feature extraction was performed using the Transformers^91^ and TIMM^92^ libraries, adapting procedures from Huh et al.^70^. All models were implemented in PyTorch^93^ and retrieved from the HuggingFace Model Hub.

For each model $m\in {{\mathcal{M}}}$, layer $l\in {{{\mathcal{L}}}}_{m}$, and stimulus set $s\in {{\mathcal{T}}}$ (images or their corresponding captions), we constructed a matrix of layer activations ${Z}_{m,l,s}\in {{\mathbb{R}}}^{{n}_{s}\times {v}_{m}}$, where *n*_*s*_ is the number of stimuli in set *s* and *v*_*m*_ is the model’s embedding size, which is constant across all layers in the selected models. For each model and stimulus set, the activations across layers define a sequence of latent representations, reflecting the hierarchical transformation of stimulus content within each model.

### Alignment measure

We used Representational Similarity Analysis (RSA)^11^ to quantify the correspondence between brain parcels and model layers' geometries. For each system, the representational dissimilarity matrix (RDM) *D*(*X*) was defined as *D*(*X*)_*ij*_ = 1 − *ρ*(*x*_*i*_, *x*_*j*_), where *x*_*i*_ and *x*_*j*_ are rows of *X* and *ρ* is the Pearson correlation. The RSA score between two systems *X* and *Y* was the correlation between the upper triangles of their RDMs:

1 $${{\rm{RSA}}}(X,Y)=\rho \left({{{\rm{vec}}}}_{u}D(X),\,{{{\rm{vec}}}}_{u}D(Y)\right).$$

Main analyses were replicated with Spearman correlation and Centered Kernel Alignment (CKA)^94^, yielding consistent results (see Supplementary Note 6). Details of the matrix reformulation that enabled efficient, large-scale RSA computations are provided in Supplementary Note 9.

### Inter-subject alignment

Inter-subject RSA was computed using the subset of 1000 images viewed by all NSD participants (the *shared1000* set)^26^. Each image in this subset was presented up to three times per participant, with corresponding trial indices across sessions. Trials were uniquely identified by participant, image ID, and repetition index *k* ∈ 1, 2, 3. For each pair of participants $p,q\in {{\mathcal{P}}}$ and cortical parcels $r,r^{\prime} \in {{\mathcal{R}}}$, we extracted the corresponding voxel responses *X*_*p*,*r*_ and ${X}_{q,r^{\prime} }$ and computed RSA between matched trials.

Images shared across participants were always presented at the same trial positions for all participants, interleaved with unique images specific to each individual. To attenuate alignment driven by task or session structure rather than by stimulus processing, we applied a cyclic repetition shift when matching trials across participants. Specifically, for each image *i* and repetition index *k*, the trial (*i*, *k*) in participant *p* was matched with the trial $(i,(k\,{{\rm{mod}}}\,\,3)+1)$ in participant *q*, thereby preserving stimulus identity while disrupting repetition-locked confounds. This matching strategy and its impact are illustrated and compared in Supplementary Note 1. Let ***i***_*pq*_ and ***j***_*pq*_ denote the matched stimulus indices for participants *p* and *q*, respectively. The inter-subject alignment between parcels *r* and $r^{\prime}$ was computed as:

2 $${{{\mathcal{A}}}}_{p,q,r,r^{\prime} }^{{{\rm{IS}}}}={{\rm{RSA}}}\left({X}_{p,r}[{{{\boldsymbol{i}}}}_{pq},:],\,{X}_{q,r^{\prime} }[{{{\boldsymbol{j}}}}_{pq},:]\right).$$

To compute a subject-level alignment measure for participant *p*, we aggregated alignment values with respect to all other participants:

3 $${{{\mathcal{A}}}}_{p,r,r^{\prime} }^{{{\rm{IS}}}}=\frac{1}{| {{\mathcal{P}}}| -1}\mathop{\sum }\limits_{q\in {{\mathcal{P}}}\backslash \{p\}}{{{\mathcal{A}}}}_{p,q,r,r^{\prime} }^{{{\rm{IS}}}}.$$

Group-level alignment was then obtained by averaging subject-level measures across participants. Cortical surface maps (e.g., Fig. 2b) display group-level alignment, specifically comparing the same parcels across participant pairs ($r=r^{\prime}$). To benchmark the degree of shared geometry relative to individual consistency, we also compared inter-subject and within-subject alignment (see Supplementary Fig. S4), which yielded consistent results.

A parcel-by-parcel matrix of group-level inter-subject alignment values (*n*_rois_ × *n*_rois_) defined the whole-cortex connectivity network. We visualized the main network backbone and multiple major representation routes using an iterative minimum spanning tree (k-MST) approach on the group-averaged connectivity matrix. At each iteration, a standard MST was computed (with edge weights defined as 1 − similarity), removing previously selected edges to ensure the inclusion of new pathways. The final backbone comprised the union of edges from all *k* MSTs (see Supplementary Fig. S7), preserving multiple principal paths, avoiding arbitrary edge thresholding, and ensuring that all parcels remained connected while allowing for richer topology. MST computations were performed using the NetworkX and SciPy libraries^95,96^.

Statistical significance was evaluated by comparing the observed alignment values to a null distribution generated by randomly shuffling stimulus labels (10,000 permutations, using a shared permutation scheme across all comparisons) with a two-tailed test. All *p*-values were FDR-corrected^97^.

### Brain-model alignment

Brain-model representational alignment was computed using all available trials from NSD participants (i.e., not restricted to the set of shared images), with analyses performed separately by session to control for session-specific variance. For each participant $p\in {{\mathcal{P}}}$, model $m\in {{\mathcal{M}}}$, and session $s\in {{{\mathcal{T}}}}_{p}$, voxel responses from each cortical parcel $r\in {{\mathcal{R}}}$ were compared to model activations at each layer $l\in {{{\mathcal{L}}}}_{m}$ using RSA:

4 $${{{\mathcal{C}}}}_{p,r,s,m}(l)={{\rm{RSA}}}\left({X}_{p,r,s},\,{Z}_{m,l,s}\right).$$

To compute a subject-level alignment measure for each model, we identified the layer with the largest absolute alignment for each session, preserving the sign of the value (denoted as ${{\rm{sign}}}\,\max$ in Eq. (5)). These peak values were then averaged across sessions:

5 $${{{\mathcal{A}}}}_{p,r,m}^{{{\rm{BM}}}}=\frac{1}{| {{{\mathcal{T}}}}_{p}| }\mathop{\sum }\limits_{s\in {{{\mathcal{T}}}}_{p}}{{{\rm{sign}}}\max }_{l\in {{{\mathcal{L}}}}_{m}}\,{{{\mathcal{C}}}}_{p,r,s,m}(l).$$

This procedure, standard in recent literature^14,70^, ensures that alignment is captured independently of model depth. In cases such as for language models, where correlations may be systematically negative, this approach preserves the interpretability of dissimilar representational geometries: negative alignment values indicate that stimuli which are close in one space (e.g., visual cortex) are systematically distant in another (e.g., language model embedding)^98^.

To identify where alignment peaked within the model hierarchy (e.g., Fig. 3b, c), we computed the normalized depth of the maximally aligned layer for each session and averaged across sessions:

6 $${d}_{p,r,m}^{* }=\frac{1}{| {{{\mathcal{T}}}}_{p}| }\mathop{\sum }\limits_{s\in {{{\mathcal{T}}}}_{p}}\frac{1}{| {{{\mathcal{L}}}}_{m}| -1}\arg \mathop{\max }\limits_{l\in {{{\mathcal{L}}}}_{m}}{{{\mathcal{C}}}}_{p,r,s,m}(l),$$

where layers are indexed from 0 (first layer) to $| {{{\mathcal{L}}}}_{m}| -1$ (last layer), so ${d}_{p,r,m}^{* }\in [0,1]$. This procedure is standard for mapping the correspondence between brain regions and network hierarchy^14,15^. To obtain a subject-level measure for a set of models (e.g., all vision or language models), both the peak alignment and normalized depth measures were averaged across models. Group-level measures were then obtained by averaging subject-level values across participants.

To study how alignment changes across the model hierarchy, we constructed continuous alignment curves for each subject parcel and model by averaging layer-wise RSA across sessions. To compare models with different numbers of layers, linear interpolation was applied, normalizing layer indices to a depth variable *d* ∈ [0, 1] (*d* = 0 for the first layer, *d* = 1 for the last layer):

7 $${\widetilde{{{\mathcal{C}}}}}_{p,r,m}(d)={{{\rm{interp}}}}_{l\to d}\left(\frac{1}{| {{{\mathcal{T}}}}_{p}| }\mathop{\sum }\limits_{s\in {{{\mathcal{T}}}}_{p}}{{{\mathcal{C}}}}_{p,r,s,m}(l)\right),\,d\in [0,1].$$

Alignment curves for each subject’s parcel across a set of models ${{\mathcal{M}}}$ (e.g., all vision models or all language models) were then obtained by averaging interpolated curves across models:

8 $${\widetilde{{{\mathcal{C}}}}}_{p,r,{{\mathcal{M}}}}(d)=\frac{1}{| {{\mathcal{M}}}| }\mathop{\sum }\limits_{m\in {{\mathcal{M}}}}{\widetilde{{{\mathcal{C}}}}}_{p,r,m}(d).$$

Statistical significance was assessed using a permutation test with 10,000 stimulus-label shuffles. The null distribution was generated by applying the same set of permutations across all model layers and parcels, allowing direct comparison and group averaging. Two-tailed *p*-values were calculated by comparing the observed group-mean alignment to this null, and corrected for multiple comparisons using the Benjamini-Hochberg FDR procedure.

### Shared component extraction

To identify the dominant dimensions underlying shared representational geometry across participants, we applied kernel multi-view canonical correlation analysis (KMCCA)^60^. KMCCA was performed on the set of image trials shared by all participants in NSD, extracting a low-dimensional subspace that maximized correlation across participants’ RDMs for each hub.

We used the KMCCA implementation from *mvlearn*^99^. For each hub, we extracted the first two components for exploratory purposes, and assessed the influence of the first (dominant) component in control analyses. While our focus was on the dominant axis, further work is needed to interpret all statistically significant components. Image-level semantic annotations and category labels were derived from MS-COCO^90^. For automated labeling of the scene-to-object gradient, we used Pixtral-12B^100^ (see Fig. 5a).

Further details of the KMCCA procedure, semantic projections, and partial RSA controls are provided in Supplementary Note 7.

### Statistics and Reproducibility

Statistical testing and analysis were carried out in Python 3.10 using standard libraries (SciPy, Statsmodels). No statistical methods were used to pre-determine sample size; sample sizes were determined by availability in the public datasets: Natural Scenes Dataset (*N* = 8), BOLD5000 (*N* = 4), and THINGS-fMRI (*N* = 3). These sizes are standard for deep-sampling fMRI studies where individual-subject reliability is high. All subjects and sessions from these datasets were included in the analysis. To assess the statistical significance of RSA maps (e.g., Fig. 2a-c), a two-tailed permutation test (*n* = 10, 000) was used to determine if RSA values significantly differed from a null distribution. For all other NSD group-level results (Figs. 1–5), significance was assessed using two-tailed paired t-tests (*N* = 8) unless otherwise specified. In all cases, *p*-values were corrected for multiple comparisons using the False Discovery Rate (FDR) or Bonferroni method, with significance defined as *p* < 0.05.

## Supplementary information

Transparent Peer Review file

Supplementary Material

## Data Availability

Functional-MRI data and image stimuli were obtained from the Natural Scenes Dataset (accessed via https://registry.opendata.aws/nsd), BOLD5000 (10.18112/openneuro.ds001499.v1.3.0 and 10.1184/R1/c.5325683), and THINGS-fMRI (10.25452/figshare.plus.c.6161151.v1 and 10.17605/osf.io/jum2f). Image metadata were extracted from MS-COCO (https://cocodataset.org). Model checkpoints analyzed in this work are available on Hugging Face (https://huggingface.co/collections/pablomm/convergent-transformations-6808f7de248fa9674acac588). Precomputed derivative data generated from these raw sources are available in Figshare 10.6084/m9.figshare.30753239. All resources were publicly accessible in April 2026.

## Code availability

Code to reproduce analyses is available at github.com/memory-formation/shared-representations and has been archived at Zenodo 10.5281/zenodo.19581037.
