# Supplementary material for: Shared representations in brains and models reveal a two-route cortical organization during scene perception
Source: Commun Biol. 2026 May 7;9:950. doi: 10.1038/s42003-026-10169-0 (PMC13365445; doi:10.1038/s42003-026-10169-0)
Supplement: Supplementary file 2 — Supplementary Material [file 42003_2026_10169_MOESM2_ESM.pdf]

Supplementary Material:

Shared representations in brains and models reveal a two-route cortical organization during scene perception

Pablo Marcos-Manchón and Lluís Fuentemilla

Extended figures

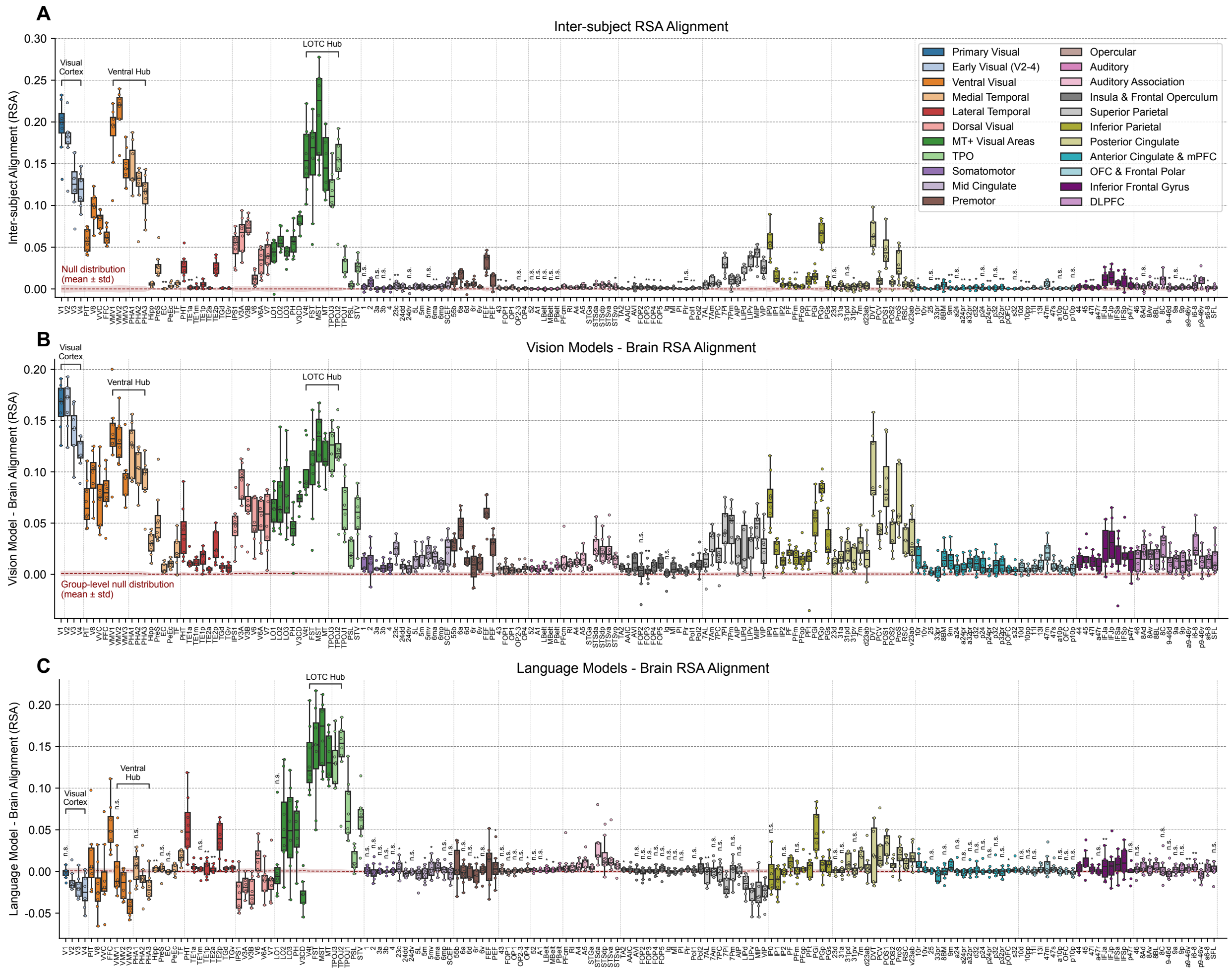

Supplementary Figure S1: **Detailed parcel-level alignment for all modalities.** Box plots show representational alignment scores computed for each of the 180 cortical parcels of the symmetric HCP atlas[1], organized by macro-anatomical groups[2]. Each box represents the distribution of alignment scores across the eight participants ($N = 8$). The red line and shaded area in each panel denote the mean and standard deviation of the null distribution, respectively, estimated via permutation testing. **(A)** Inter-subject alignment (IS-RSA). **(B)** Brain-to-vision-model alignment. **(C)** Brain-to-language-model alignment. This figure provides a detailed view of the results summarized in the main text, showing the parcel-by-parcel variability and confirming the concentration of high alignment within the Early Visual, Ventral, and LOTC hubs.

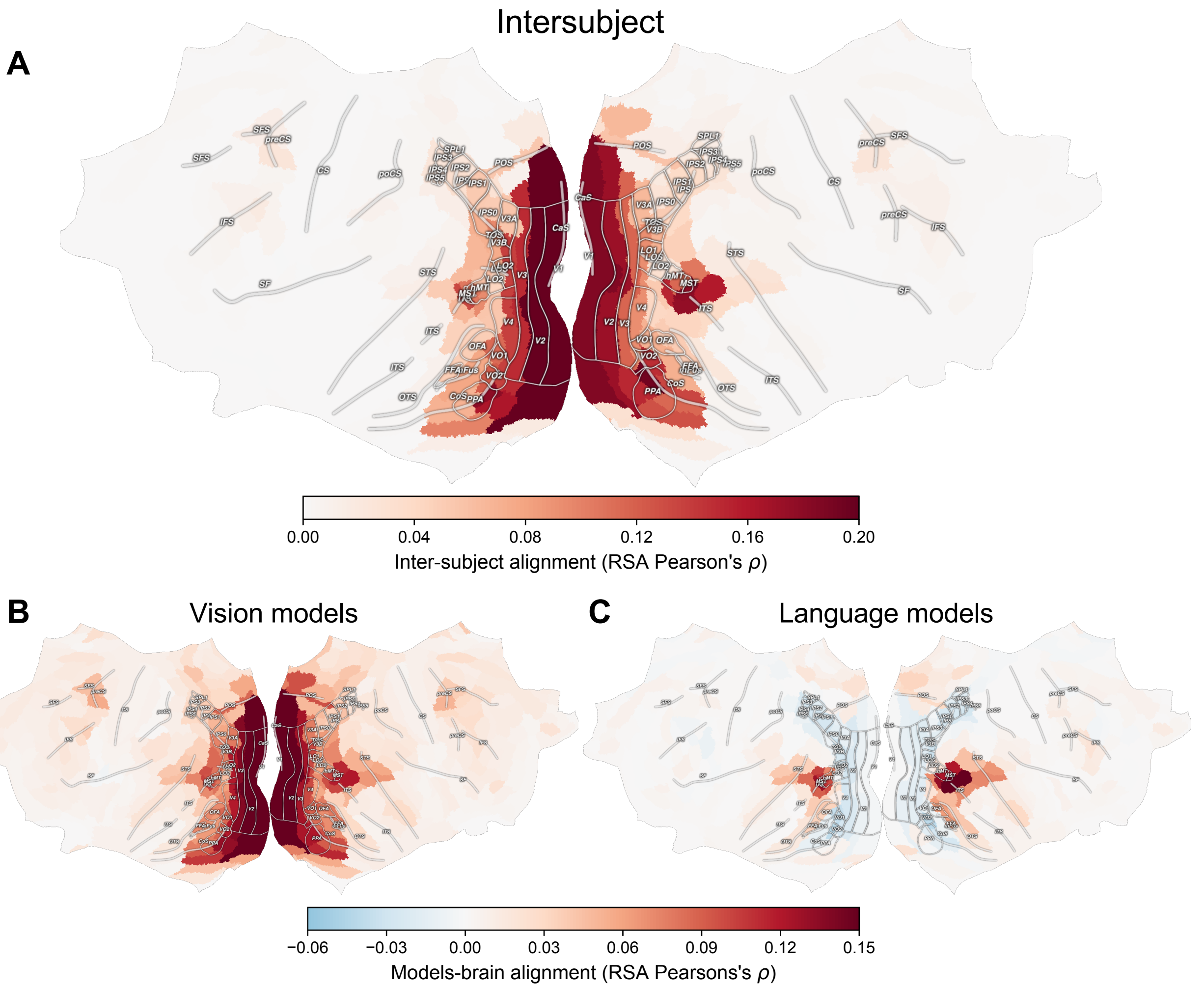

Supplementary Figure S2: **Cortical flat map projections of representational alignment.** To provide a comprehensive view of their spatial distribution, group-level alignment scores are projected onto a flattened cortical surface. The figure shows **(A)** IS-RSA alignment, **(B)** vision-model alignment, and **(C)** language-model alignment. Values represent group-averaged RSA scores (Pearson's ρ, $N = 8$ from the NSD dataset), and major sulci are labeled for anatomical orientation. This visualization makes the full extent of the alignment patterns clear, particularly highlighting the widespread negative alignment (cool colors) between language models and the ventral visual stream, in contrast to the positive alignment seen for inter-subject and vision-model comparisons.

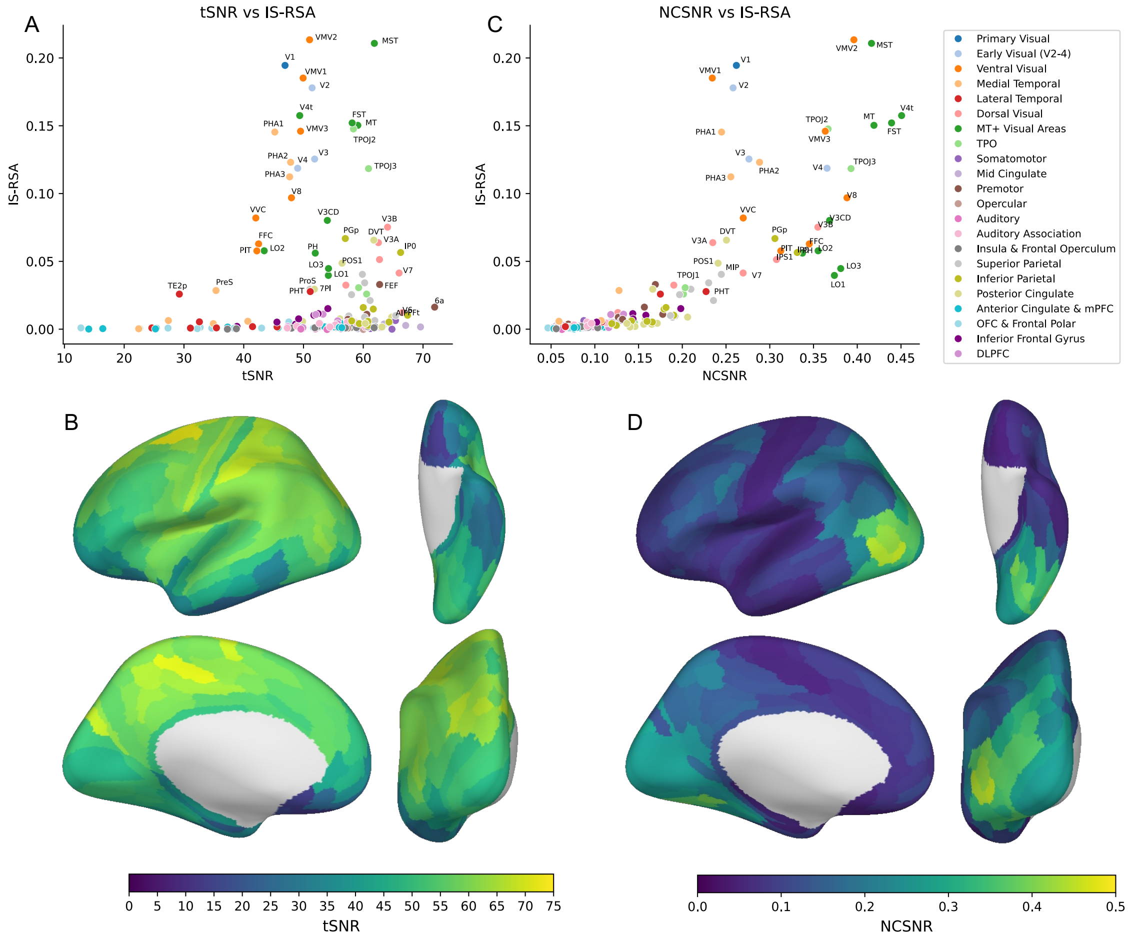

Supplementary Figure S3: **Relation between signal-to-noise metrics and inter-subject representational alignment.** **(A,C)** Scatter plots showing the relationship between parcel-wise signal-to-noise measures and inter-subject representational similarity (IS-RSA). **(A)** Mean temporal signal-to-noise ratio (tSNR) per ROI versus IS-RSA. tSNR was computed from the preprocessed BOLD time series of the first session as the mean signal divided by its standard deviation across time, estimated within each run and then averaged across runs, parcels, and subjects. **(C)** Mean noise-ceiling SNR (NCSNR) per ROI versus IS-RSA. NCSNR is the voxel-wise reliability metric provided in the NSD dataset[3], derived from trial-wise beta responses across repeated presentations of the same image; values were averaged within each ROI and across subjects. Each point corresponds to one symmetric ROI, obtained by averaging homologous parcels across hemispheres; colors indicate large-scale cortical systems. **(B,D)** Cortical surface maps showing the spatial distribution of the same parcel-wise measures. **(B)** Mean tSNR per ROI. **(D)** Mean NCSNR per ROI. Across parcels, tSNR showed no clear association with IS-RSA, whereas NCSNR was positively associated with IS-RSA, indicating that stimulus-locked response reliability constrains, but does not fully determine, the spatial distribution of shared representational geometry across cortex.

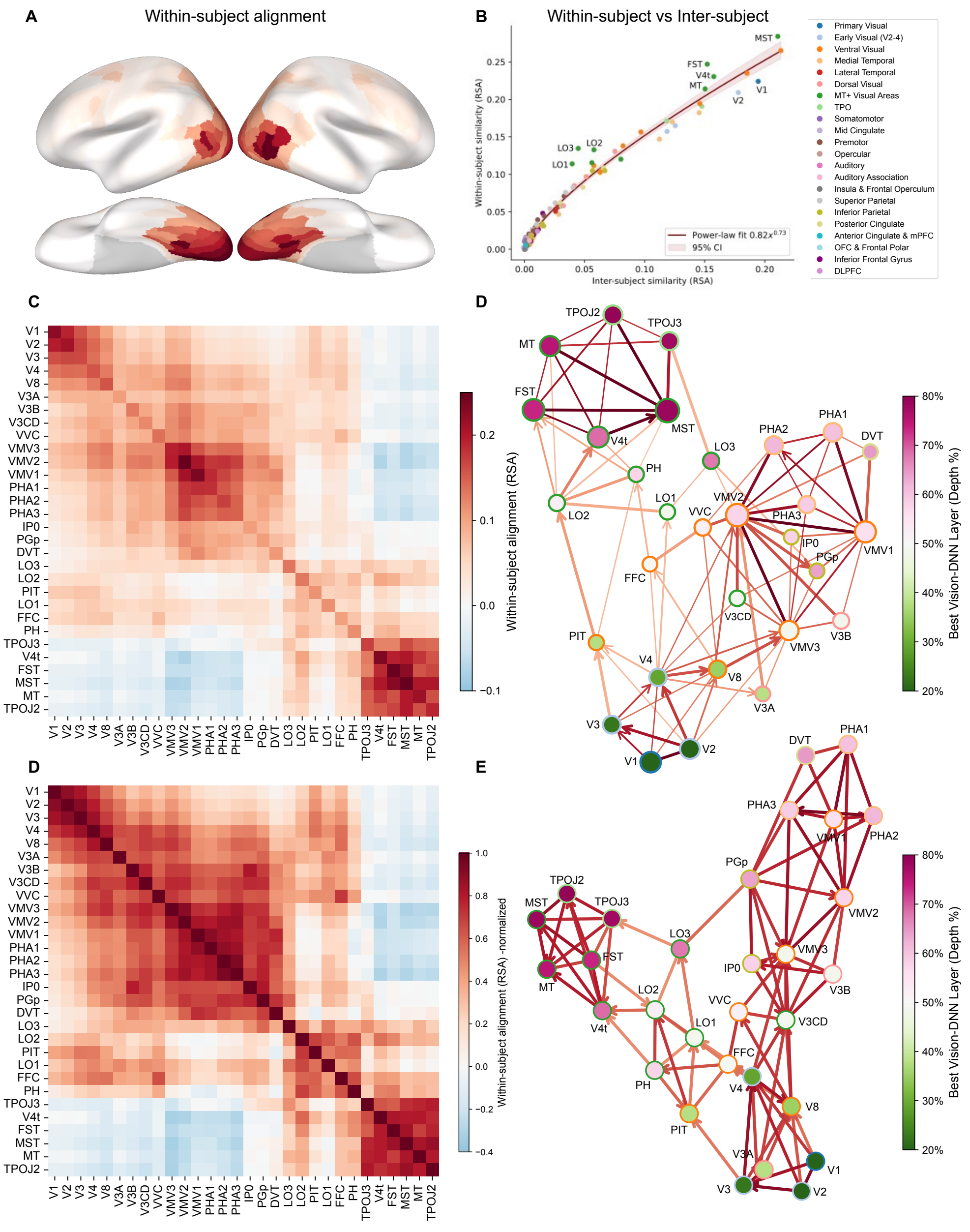

Supplementary Figure S4: **Comparison of within-subject and inter-subject representational analyses. (A) Cortical map of within-subject alignment** (RSA, Pearson's r). This was computed analogously to the inter-subject analysis by comparing RDMs from different trial repetitions within each participant, and then averaging across participants ($N = 8$). The map reveals a similar spatial distribution to the inter-subject version, with a posterior-to-anterior gradient and three distinct hubs. **(B) Parcel-wise comparison of within-subject and inter-subject alignment**. The two measures are tightly correlated across the cortex, following a power-law relationship (fit shown in red; $R^2 = 0.98$). This indicates that brain regions showing stronger within-subject consistency also tend to show stronger consistency across individuals. **(C) Within-subject representational connectivity matrix**. Computed by correlating RDMs between all pairs of parcels within each participant, this analysis reveals the same three-hub block structure discovered using the inter-subject measurements. **(D) Directed connectivity graph of the within-subject data**, pruned using a 3-minimum spanning tree to highlight the network backbone. Edge color and width encode the inter-region alignment (RSA value), while node color represents each parcel's peak alignment depth with vision models. Directionality is inferred from this depth measure based on a low-to-high sequential hierarchy, with arrows pointing from shallower- to deeper-aligning regions. The graph confirms the same information flow as the inter-subject analysis, with two streams emerging from early visual cortex: a medial-ventral stream to the Ventral hub and a lateral-dorsal stream to the LOTC hub. **(E) Within-subject representational connectivity matrix normalized by within-ROI alignment.** The normalized matrix preserves the same broad three-hub organization as the unnormalized analysis. **(F) Directed connectivity graph derived from the normalized within-subject connectivity matrix.** The normalized analysis recovers the same dual-stream topology, with only minor differences in specific ROI-to-ROI connections.

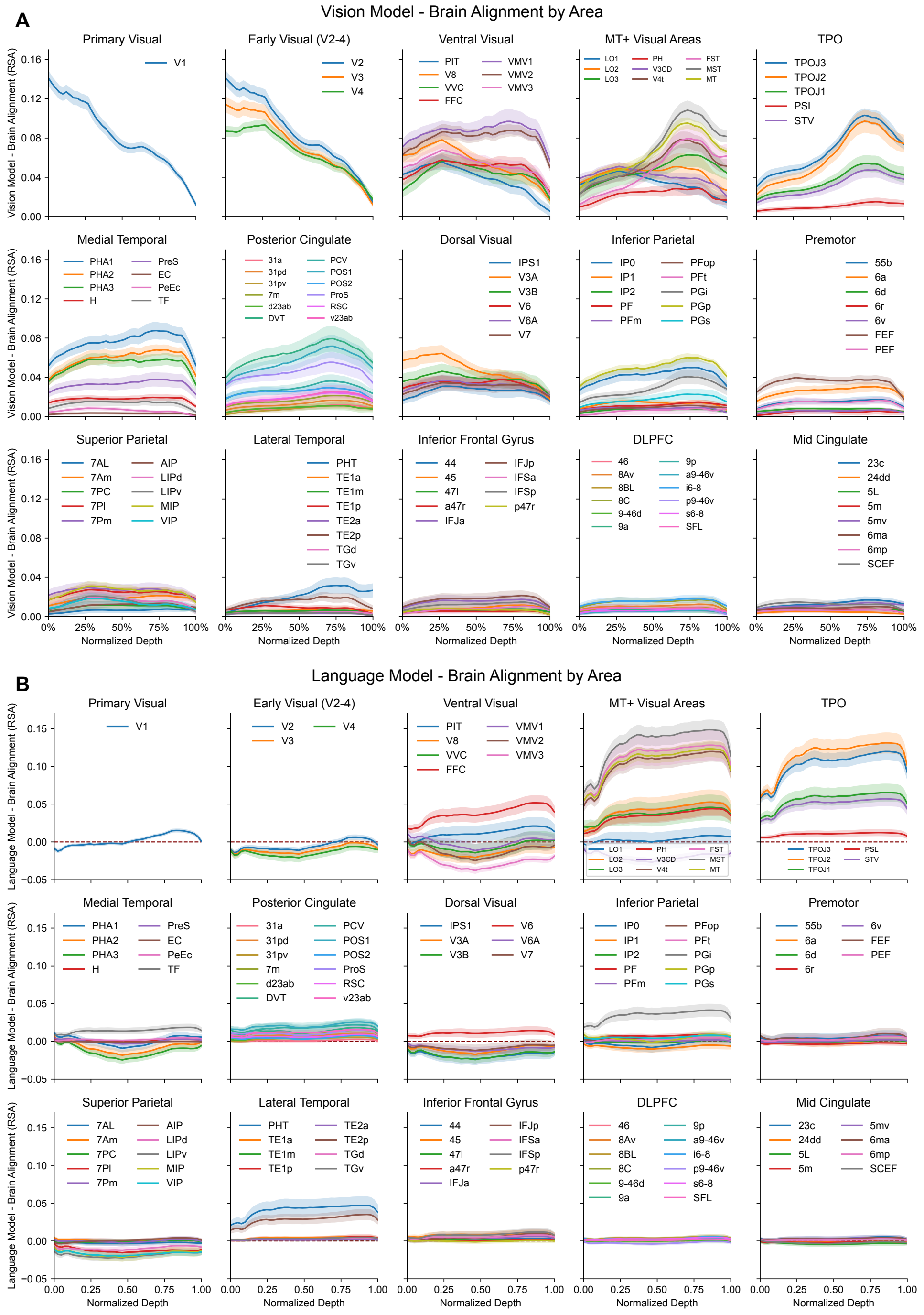

Supplementary Figure S5: **Detailed layer-wise alignment profiles across cortical parcels.** Detailed parcel-by-parcel view of the brain-model alignment curves summarized in the main text (Fig. 3), showing results for the 15 macro-anatomical groups with the highest inter-subject alignment. Only parcels that were themselves statistically significant in the inter-subject analysis are displayed. For each modality, the plotted alignment curves represent the RSA score averaged across all models of that type. Each individual line shows the mean alignment for a single parcel across participants ($N = 8$), with shaded areas indicating the SEM. **(A) Brain-to-vision-model alignment**. This detailed view confirms that the distinct alignment profiles–decreasing, distributed, and increasing–are characteristic of the main hubs and often extend to anatomically adjacent parcels (e.g., the increasing profile of the LOTC hub is also observed in nearby STS regions). In contrast, areas with lower overall correspondence, such as in the prefrontal cortex, tend to show weak alignment across all model layers without a clear hierarchical preference. **(B) Brain-to-language-model alignment**. This view confirms that positive alignment is restricted to the LOTC hub and a small number of anatomically proximal parcels, all of which consistently exhibit the same step-like alignment profile.

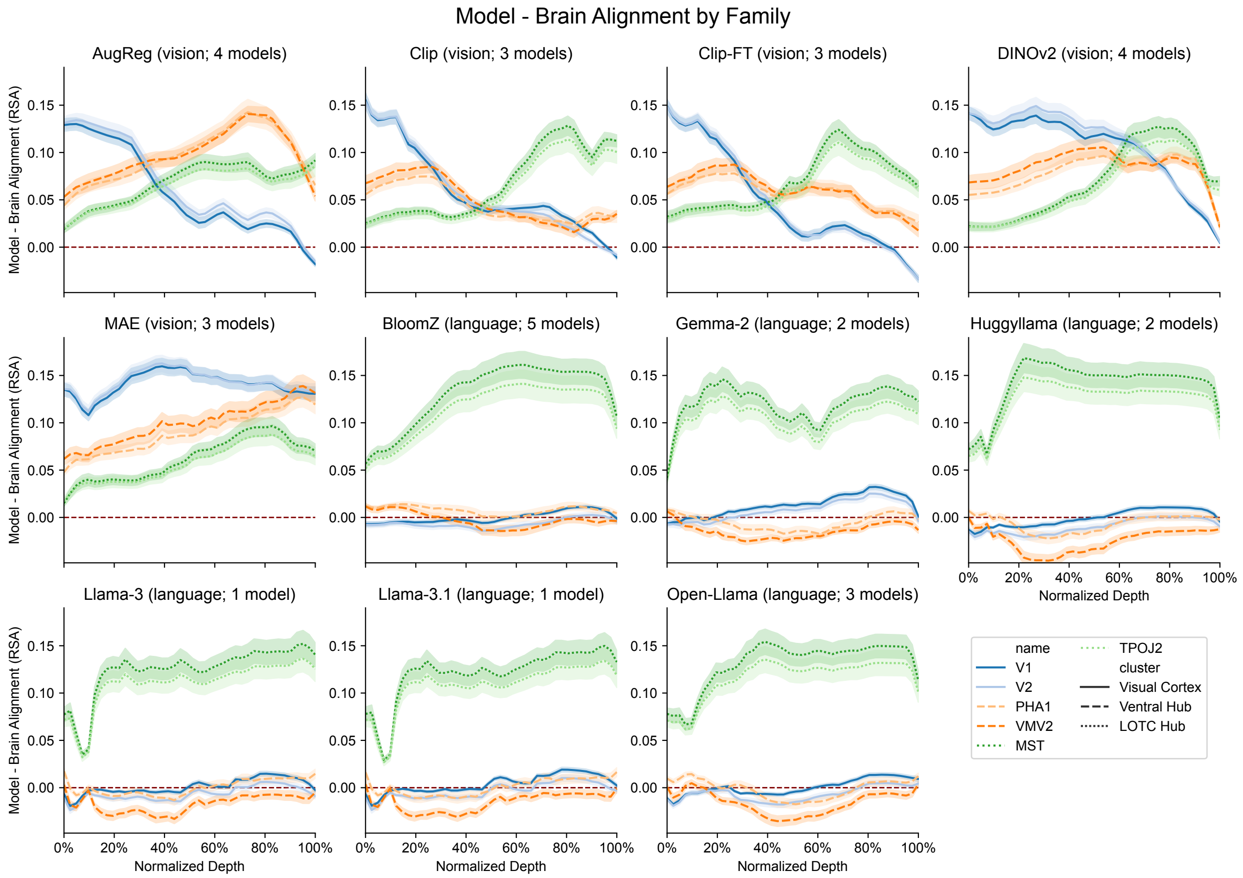

Supplementary Figure S6: **Brain-model alignment profiles for different model families.** Layer-wise alignment (RSA, Pearson's r) is shown for representative parcels from the three main hubs, computed separately for each family of models (see Table 1 for a full list). Each curve represents the mean alignment across participants ($N = 8$), with shaded areas indicating the SEM. **Vision Models:** Most vision model families (AugReg, CLIP, DINOv2) reproduce the characteristic hierarchical profiles: a decreasing alignment with Early Visual Cortex, a distributed alignment with the Ventral Hub, and an increasing alignment with the LOTC Hub. The Masked Autoencoder (MAE) models are a notable exception; consistent with their reconstructive training objective, they maintain a high alignment with Early Visual Cortex across all layers. **Language Models:** Despite differences in architecture, training data, and fine-tuning (e.g., instruction tuning in BLOOMZ), all language model families exhibit a similar pattern. Alignment is negligible or negative for Early Visual and Ventral hub parcels, while all families show the same characteristic step-function profile for parcels in the LOTC hub.

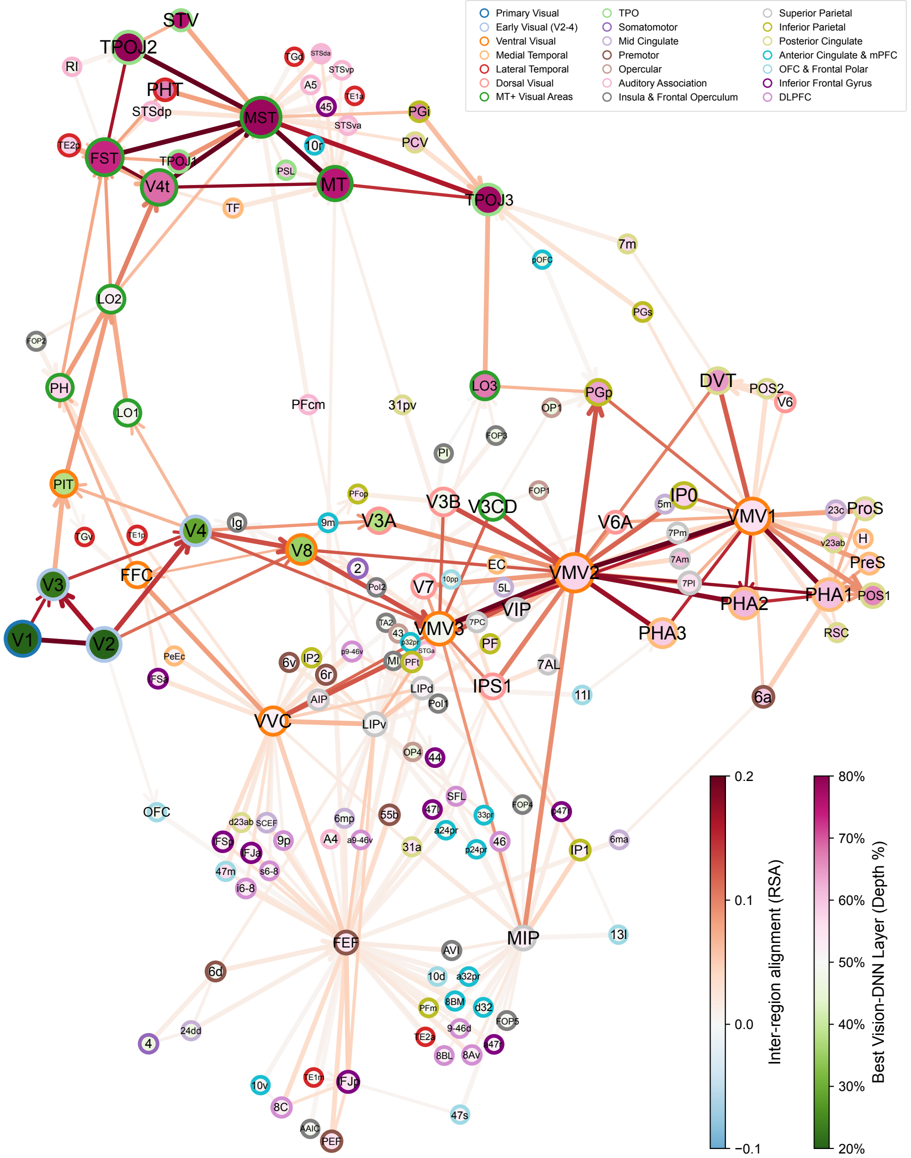

Supplementary Figure S7: **Whole-cortex representational connectivity network.** Representational connectivity network among the 157 cortical parcels that exhibited statistically significant inter-subject alignment. The network is pruned using a 2-minimum spanning tree to highlight the strongest connections. To minimize confounds from anatomical variability, connectivity was computed on a within-subject basis ($N = 8$); for each participant, we correlated the RDM of every parcel with every other parcel across different repetitions of the same stimuli, then averaged the resulting connectivity matrices. Edge color encodes the strength of this inter-region alignment (RSA), while edge width indicates the spanning tree order (1st or 2nd) to highlight the most central pathways. Node color represents each parcel's peak alignment depth with vision models, providing a proxy for its hierarchical position. Arrows are added to the pruned edges to denote a statistically significant difference in this depth between connected parcels (paired t-test, $t(7)$, FDR-corrected $p < 0.05$), providing a descriptive ordering of edges along the model-derived hierarchy. The graph recovers the two primary visual processing streams identified in the main text. A medial-ventral stream connects early visual areas to core ventral stream regions involved in scene and object processing. In parallel, a lateral-dorsal stream connects early visual areas to the LOTC hub, including motion-sensitive areas (MT+ complex) and higher-order regions in the superior temporal sulcus (STS) and temporoparietal junction (TPOJ). The analysis also reveals key bridges between these streams, with nodes such as LO3 and PGp linking the ventral and LOTC hubs. Furthermore, despite weaker overall alignment, frontal and parietal regions like the Frontal Eye Fields (FEF) and the Medial Intraparietal Area (MIP) emerge as distinct hubs, suggesting their integration into this large-scale, stimulus-driven network.

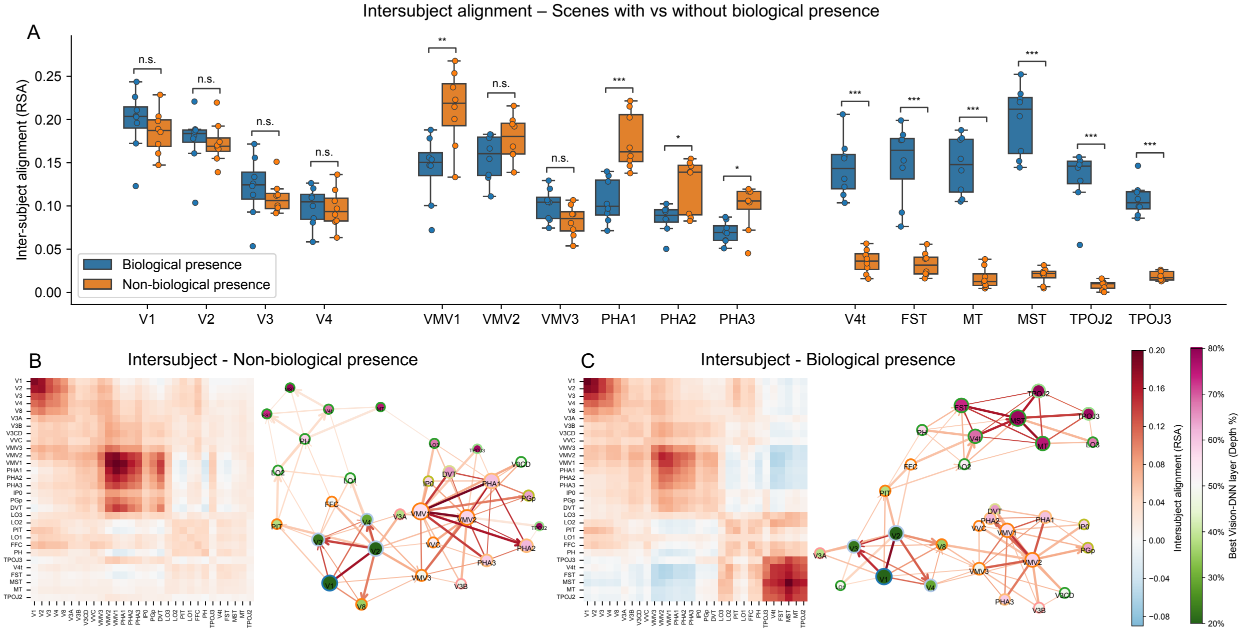

Supplementary Figure S8: **Biological content selectively drives LOTC alignment and lateral-stream connectivity.** Scenes were split into those that contained biological agents (people or animals; 65.3% of the shared image set) and those that did not, and all inter-subject analyses were recomputed on each subset. **(A)** Parcel-wise inter-subject RSA within the three hubs. Blue: scenes with biological agents; orange: scenes without them. Early Visual parcels (V1–V4) show no reliable difference, Ventral parcels (VMV1–PHA3) align more for non-biological scenes, whereas LOTC parcels (V4t–TPOJ3) align almost exclusively for biological scenes. Stars mark two-tailed paired t-tests across eight participants ($^{*}p < 0.05$, $^{**}p < 0.01$, $^{***}p < 0.001$, FDR-corrected). **(B,C)** Group-level representational-connectivity matrices (left) and backbone graphs (right) for scenes *without* (**B**) and *with* (**C**) biological agents. Backbones were extracted with a three-iteration minimum-spanning-tree procedure. Node colour encodes each parcel's peak vision-model layer (early → late: green → purple), node size scales with within-parcel IS-RSA, edge hue shows pairwise RSA, and edge width denotes the iteration (1–3) at which the edge entered the spanning tree. The lateral stream linking Early Visual Cortex to LOTC is largely absent for non-biological scenes (B) but re-emerges when biological agents are present (C), mirroring the parcel-level results in (A).

Supplementary Notes

Supplementary Note 1: Repetition-Matching Strategies for Inter-Subject RSA

The Natural Scenes Dataset (NSD) presents 10,000 unique images across up to 40 scanning sessions per participant (30,000 trials total). A subset of 1,000 images (3,000 trials) was shared by all eight participants and always appeared in the same trial positions–interleaved among participant-unique stimuli–so that each shared image was viewed under identical practice, fatigue, and session-context conditions. This locked structure raises the possibility that inter-subject correlations might reflect non-perceptual factors (e.g., trial timing or memory demands) rather than purely stimulus-driven geometry.

To isolate the stimulus-driven component, we adopted a "shifted-repetition" matching strategy when computing inter-subject representational similarity (IS-RSA). Rather than pairing each repetition index $k \leftrightarrow k$ across participants ("unshifted"), we cyclically permute so that repetition k in participant p matches repetition $(k \bmod 3) + 1$ in participant q (i.e. $1 \to 2$, $2 \to 3$, $3 \to 1$; see Supplementary Fig. S9A). Because all shared trials occupy the same session slots, this shift preserves image identity while breaking any exact repetition-locked confounds and also allows direct comparison

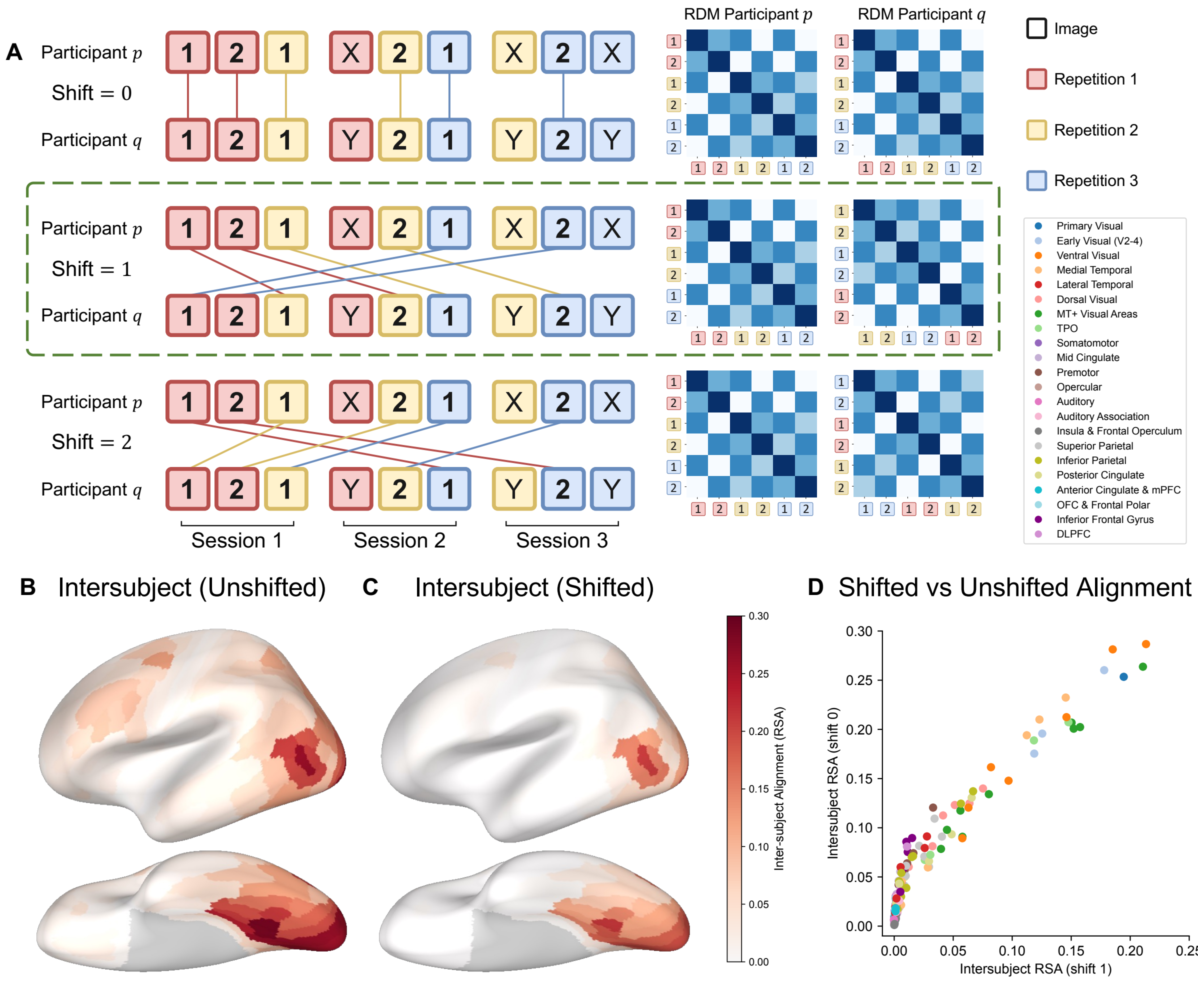

Supplementary Figure S9: **Repetition-matching strategies and their impact on inter-subject RSA.** To compute inter-subject RSA, we compared different strategies for matching the three trial repetitions for each shared image between any pair of participants. **(A)** Schematic of unshifted (repetition 1→1, 2→2, 3→3) versus shifted (cyclic 1→2, 2→3, 3→1) trial matching with example RDMs. **(B, C)**Group-average cortical RSA maps under unshifted **(B)** and shifted **(C)** matching; both reveal the early-visual, ventral, and LOTC hubs. **(D)** Parcel-wise comparison of shifted vs. unshifted RSA values shows a uniform attenuation under shifted matching, with two linear regimes reflecting stronger prefrontal attenuation.

with within-subject analyses (which must compare different repetitions; see Supplementary Fig. S4). Due to symmetry across participant-pair comparisons, shifts of 1 and 2 yield identical group-average maps.

Recomputing the group-average cortical RSA under both unshifted (Supplementary Fig. S9B) and shifted (Supplementary Fig. S9C) schemes recovers the same three hubs–early visual cortex, ventral, and LOTC. As expected, shifting is more conservative: alignment magnitudes are uniformly lower (Supplementary Fig. S9D), yet the relative rank-order of all parcels remains unchanged (Spearman's $\rho = 0.96$). Frontal parcels (e.g. FEF, inferior frontal gyrus) suffer proportionally larger attenuation under shifting than occipito-temporal hubs, suggesting that these frontal regions encode task- or repetition-related variance (e.g. decision or attentional control) that the shifted criterion minimizes. By contrast, the robust persistence of occipital and temporal alignments underscores their stable, stimulus-driven representational geometry.

To illustrate how these differences manifest in network structure, Supplementary Fig. S10 shows representational connectivity graphs for both matching strategies. We first identified parcels whose inter-subject RSA exceeded an absolute threshold of $r = 0.05$ in either the shifted (shift = 1 or 2) or unshifted maps. We then constructed a reduced connectivity matrix among this common set of nodes and extracted a two-iteration minimum-spanning-tree backbone (using edge weights defined as $1 - \mathrm{RSA}$; see main Methods). The core two-stream topology (Early Visual $\rightarrow$ Ventral hub and Early Visual $\rightarrow$ LOTC hub) is preserved under both schemes. However, only the unshifted graph highlights FEF as a high-centrality node, further suggesting that frontal regions carry shared task- or repetition-locked signals that are attenuated by the shifted criterion.

While these frontal effects are exploratory–given their lower RSA magnitudes and sensitivity to thresholding–they point to important future work on how task and attention signals coexist with stimulus-driven representations.

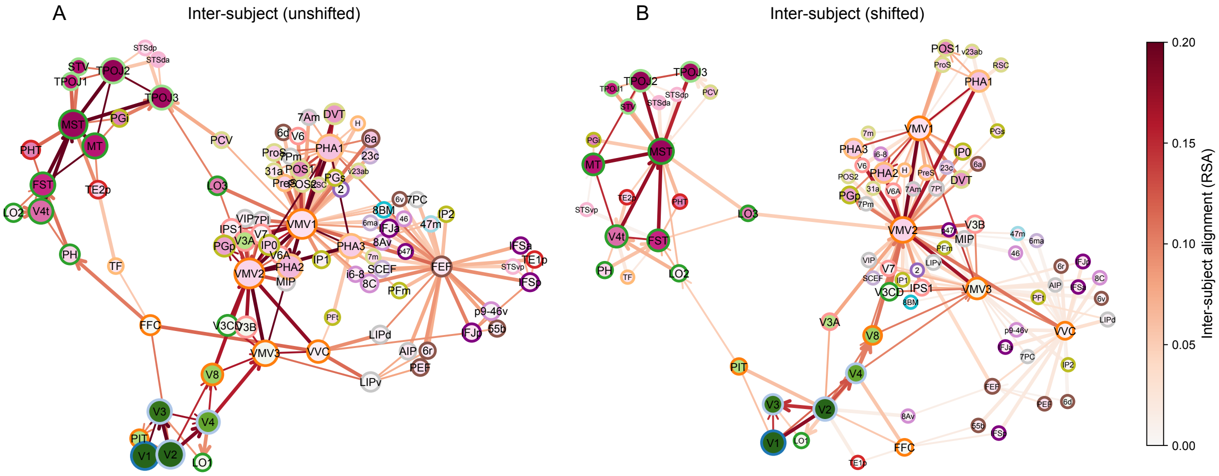

Supplementary Figure S10: **Representational connectivity backbones under unshifted vs. shifted matching.** Parcels with inter-subject RSA > 0.05 in either scheme are connected via a two-iteration minimum-spanning-tree (edge cost = 1–RSA). **A**: unshifted matching; **B**: shifted matching. Both backbones preserve the Early Visual→Ventral and Early Visual→LOTC streams.

Supplementary Note 2: Parcel size and the reliability of IS-RSA estimates

HCP–MMP parcels vary substantially in surface area and voxel count, raising the concern that IS-RSA estimates might be systematically less stable in smaller parcels due to reduced signal averaging. To evaluate this, we quantified the dispersion of IS-RSA estimates under two resampling schemes that isolate distinct sources of variability: changes in stimulus sampling and trial-level measurement noise.

To assess stability with respect to stimulus sampling, we recomputed IS-RSA across 1,000 random half-splits of the image set (500 unique images per split, including all repetitions; Supplementary Fig. S11a-d). For each parcel, we computed the coefficient of variation (CV; standard deviation of the distribution divided by the mean) as a scale-normalized index of estimation stability.

To isolate trial-level measurement variability while holding stimulus content fixed, we performed 1,000 iterations in which we randomly selected a single repetition per image (from the 2–3 available) and recomputed IS-RSA (Supplementary Fig. S11e-h). Dispersion was again summarized per parcel using the CV.

Across both analyses, parcels with similar mean IS-RSA exhibited comparable dispersion despite large differences in voxel count (Supplementary Fig.S11a-b, e-f). Critically, CV showed no systematic dependence on parcel size (Supplementary Fig.S11c,g). Instead, dispersion decreased monotonically with the magnitude of IS-RSA (Supplementary Fig. S11d,h), indicating that parcels with stronger shared geometry yield more stable IS-RSA estimates regardless of their size. These results suggest that, under the NSD preprocessing regime, the stability of IS-RSA estimates is primarily governed by the strength of the shared representational signal rather than by the number of voxels available for averaging.

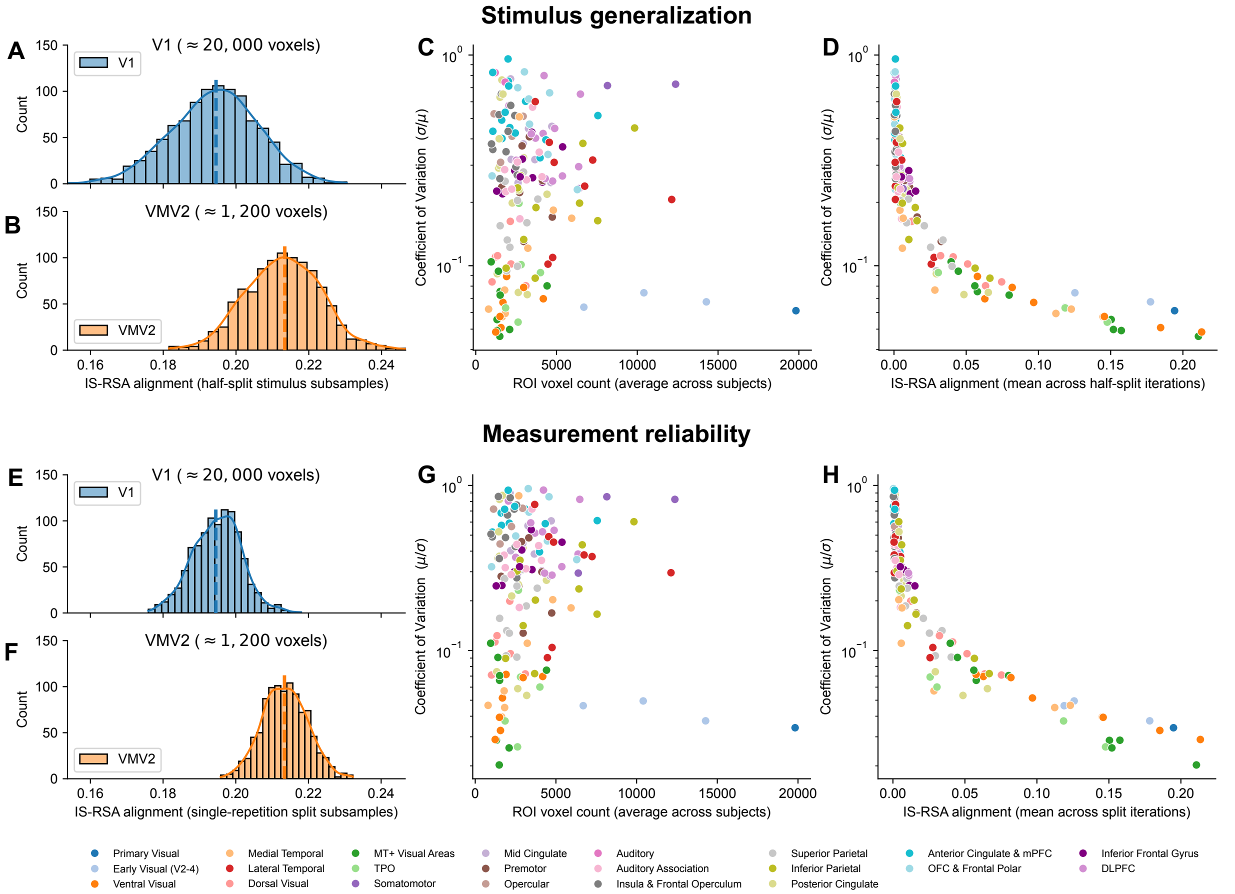

Supplementary Figure S11: Quantifying parcel reliability via resampling. **(A–D)** Stimulus generalization analysis. We computed IS-RSA on 1,000 random half-splits of the stimulus set (selecting 500 stimuli in each subsample). **(A,B)** Distribution of IS-RSA scores for a representative large parcel (V1) and small parcel (VMV2). Dashed vertical lines indicate the IS-RSA estimate from the full dataset. **(C)** IS-RSA coefficient of variation (CV; std/mean) plotted against parcel voxel count. **(D)** CV plotted against mean IS-RSA alignment. **(E–H)** Measurement reliability analysis based on randomly selecting one repetition per image across 1,000 iterations. **(E,F)** Corresponding distributions for V1 and VMV2. **(G)** CV versus voxel count. **(H)** CV versus mean IS-RSA alignment. In both analyses, the dispersion shows no dependence on voxel count but decreases as a function of the IS-RSA magnitude.

Supplementary Note 3: Hemispheric Asymmetry and the Validity of Symmetric Analyses

We quantified hemispheric asymmetry in representational geometry by computing alignment measures separately for the left and right hemispheres using the HCP-MMP1.0 atlas[1]. For each cortical parcel, we assessed inter-subject alignment, vision-model alignment, and language-model alignment using the same NSD responses as in the main analyses (Supplementary Fig. S12).

Across all modalities, a largely symmetric pattern was observed (Fig. S12a-c). The rank-order of parcels was highly consistent between hemispheres (Spearman's ρ: inter-subject $= 0.93$, vision $= 0.92$, language $= 0.82$). However, specific asymmetries emerged: early visual cortex (V1–V4) consistently showed stronger alignment in the left hemi-

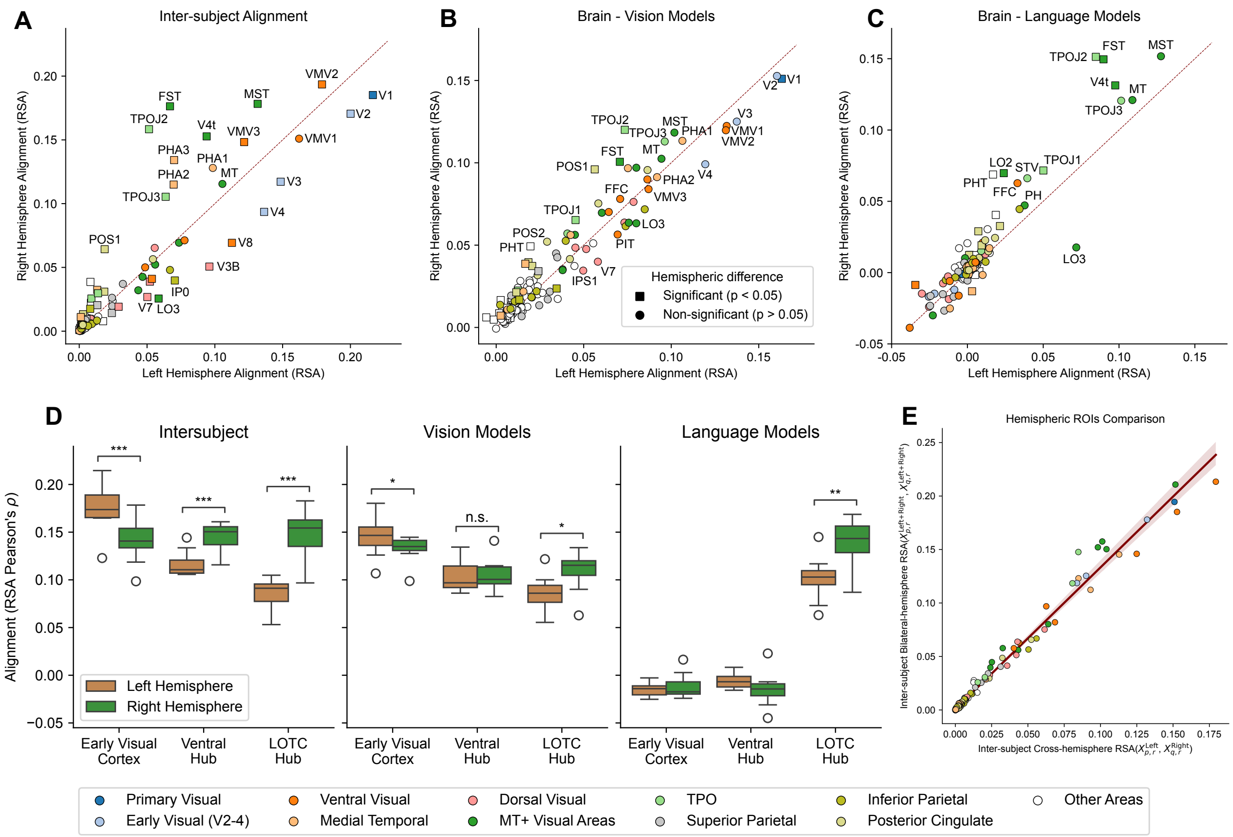

Supplementary Figure S12: **Hemispheric comparison of representational alignment.** **(A-C)** Comparison of representational alignment (RSA, Pearson's r; $N = 8$, NSD) computed independently for the left (x-axis) and right (y-axis) hemispheres: **(A)** inter-subject alignment, **(B)** vision-model alignment, and **(C)** language-model alignment. Each point is a cortical parcel, colored by macro-anatomical group. The diagonal line indicates equal alignment. Squares denote parcels with a statistically significant hemispheric difference (paired t-test, $p < 0.05$, FDR-corrected). **(D)** Boxplots of alignment within principal hubs. **(E)** Correlation between cross-hemispheric and bilateral inter-subject alignment across parcels. Significant hemisphere effects are marked.

sphere, while the ventral and LOTC hubs (e.g., hMT+, LO, TPOJ) showed stronger alignment in the right hemisphere (Fig. S12d). These lateralization trends were stable across inter-subject and model-based analyses.

To determine whether these asymmetries reflected fundamental differences in representational geometry or merely differences in signal strength, we compared cross-hemispheric RSA (e.g., subject p's left vs subject q's right) to bilateral RSA. These measures were near-perfectly correlated across parcels ($r = 0.99$, Fig. S12e), with no parcel deviating substantially from the regression line. This demonstrates that combining hemispheres increases overall alignment strength (likely via noise averaging) without distorting the underlying representational geometry; no region exhibited a lateralized geometry that was absent in the contralateral hemisphere.

Together, these results validate our strategy of pooling hemispheres for the main analyses: aggregation maximizes statistical power for multivariate comparisons without introducing representational artifacts, while still allowing for targeted lateralization analyses where relevant.

Supplementary Note 4: Power-Law Attenuation Model of RSA Metrics

In the main text, we compared several RSA measures–including within-subject (WS), inter-subject (IS-1: shift=1 and IS-0: unshifted), and vision-model-brain (VM)–that probed related aspects of common cortical geometry under different comparison regimes. While all metrics revealed a similar cortical alignment pattern, their absolute magnitudes varied considerably. Parcel-wise scatter plots (e.g., Fig. 2e; Supplementary Fig. S9) showed a systematic relationship between the metrics, which were tightly related by power-law curves. Although these curves differed in scale and curvature,

their consistent functional form suggested that each metric captured a common parcel-wise organization, expressed with distinct attenuation profiles and overall magnitudes.

We formalize this observation by modeling the correlation measured for parcel r with metric j as a power-law transform of a latent shared-geometry variable:

$$\rho_{rj}^{\text{obs}} = a_j \left(\rho_r^{\text{latent}}\right)^{b_j} + \epsilon_{rj}, \tag{1}$$

where:

- $\rho_r^{\text{latent}} \in [0, 1]$ is a latent parcel-wise variable common to all measures, capturing their shared ordering across cortex;
- $a_j \in [0, 1]$ is a metric-specific scale term that controls the overall magnitude of the observed alignment;
- $b_j \geq 1$ captures nonlinear attenuation. A value of $b_j = 1$ corresponds to simple linear scaling, whereas $b_j > 1$ creates a convex curve that suppresses weaker latent values more strongly than stronger ones. This parameter therefore describes how strongly each metric compresses low-alignment parcels;
- ϵ_{rj} is a residual term absorbing parcel-metric specific variability not captured by the deterministic power-law component.

We jointly fitted the parameters of Eq. 1 to four core metrics (WS, IS-0, IS-1, VM) using data from the symmetric-hemisphere ROIs. Parameter estimation was performed via the L-BFGS-B algorithm by minimizing the sum of squared residuals, with bounds: $a_j \in [0, 1]$, $b_j \in [1, \infty)$, and $\rho_r^{\text{latent}} \in [0, 1]$.

Supplementary Figure S13A demonstrates the model's high goodness-of-fit. In all cases, the model explained over 94% of the variance ($R^2 > 0.94$), confirming that the power-law formulation accurately captures the empirical relationships among the observed RSA measures. Suppl. Figure S13B visualizes the distinct curve for each RSA strategy, illustrating how the different metrics map the same latent parcel-wise ordering onto different observed scales and attenuation profiles.

Because all metrics are modeled as functions of the same latent variable, ρ_r^{latent}, the relationship between any two metrics, j and j', can be derived analytically by eliminating this common term:

$$y = \frac{a_{j'}}{a_j^{\,b_{j'}/b_j}}\, x^{\,b_{j'}/b_j}, \tag{2}$$

where $x = \rho_{rj}^{\text{obs}}$ and $y = \rho_{rj'}^{\text{obs}}$. Supplementary Figure S13c validates this prediction by overlaying the derived curve (Eq. 2) on the empirical parcel scatters. The tight alignment confirms that a common latent shared-geometry variable, together with metric-specific scale and attenuation, explains the pairwise relationships. For completeness, Figure S14 extends this validation to a set of 12 metrics, demonstrating the model's ability to unify all RSA strategies used in our analyses.

The resulting cortical map of the latent variable, $\hat{\rho}_r^{\text{latent}}$, is displayed in Supplementary Figure S13D. This map should be interpreted as an estimate of the common parcel-wise organization underlying the core RSA measures after accounting for metric-specific scale and attenuation. It recovers the three-hub pattern observed in the main text, with peak values ($\rho_r^{\text{latent}} \sim 0.50$) in the EVC, Ventral stream, and LOTC. Furthermore, the model suggests that frontal areas (e.g., FEF and PFC) become more prominent after accounting for the stronger attenuation present in some raw measurements. The fact that these hubs align with those highlighted by individual metrics indicates that Eq. 1 captures a common shared-geometry organization across metrics, rather than a property specific to any single comparison.

Note that because Eq. 1 is scale-free, the absolute values of $\hat{\rho}^{\text{latent}}$ and a_j are defined only up to a common scaling factor. We fixed no anchor during optimization, yet repeated fits converged to the same scale, and all qualitative findings (relative parcel ranking, cross-metric relations) are invariant under rescaling.

The power-law attenuation model offers a compact, quantitative account of the nonlinear relations among the RSA measures used in this study. Its success supports the view that all metrics probe a shared latent shared-geometry organization, while differing in their scale and attenuation profile, rather than constituting directly comparable estimates of a single quantity.

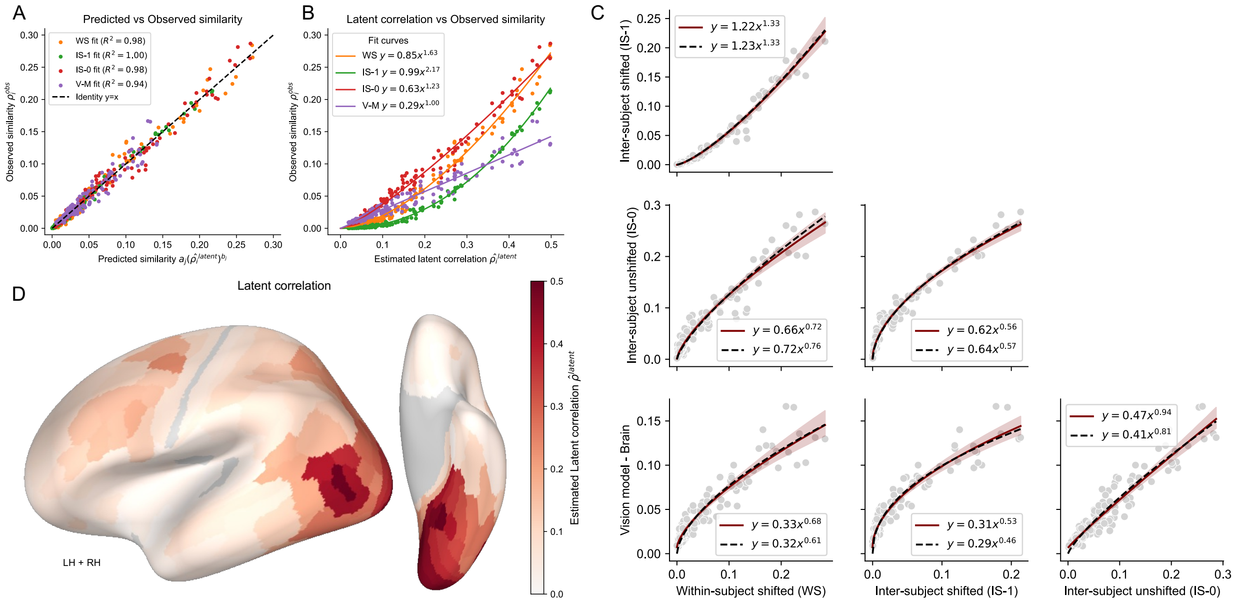

Supplementary Figure S13: **Power-law attenuation model. (A) Goodness-of-fit.** Parcel-wise observed RSA values (y-axis) plotted against predictions from the joint power-law model (Eq. 1; x-axis). Colors denote metrics (WS: within-subject; IS-1: inter-subject shifted; IS-0: inter-subject unshifted; VM: vision model). Legend indicates explained variance (R^2). **(B) Metric-specific curves.** Estimated latent shared-geometry values ($\hat{\rho}^{\text{latent}}$, x-axis) vs. observed correlation (y-axis). Colored lines show the fitted metric-specific curves ($y = a_j x^{b_j}$). **(C) Pairwise comparisons.** Scatter plots for six representative metric pairs (gray points). Each plot includes an independent power-law fit (solid red line, 95% CI) and the prediction from the joint model (dashed black line, Eq. 2), demonstrating high correspondence. **(D) Latent shared-geometry map.** Cortical surface map of the estimated latent values, $\hat{\rho}_r^{\text{latent}}$ (HCP-MMP1 atlas, symmetric parcels). The model recovers the three major hubs (EVC, Ventral, LOTC) and suggests additional structure in prefrontal regions after accounting for metric-specific attenuation.

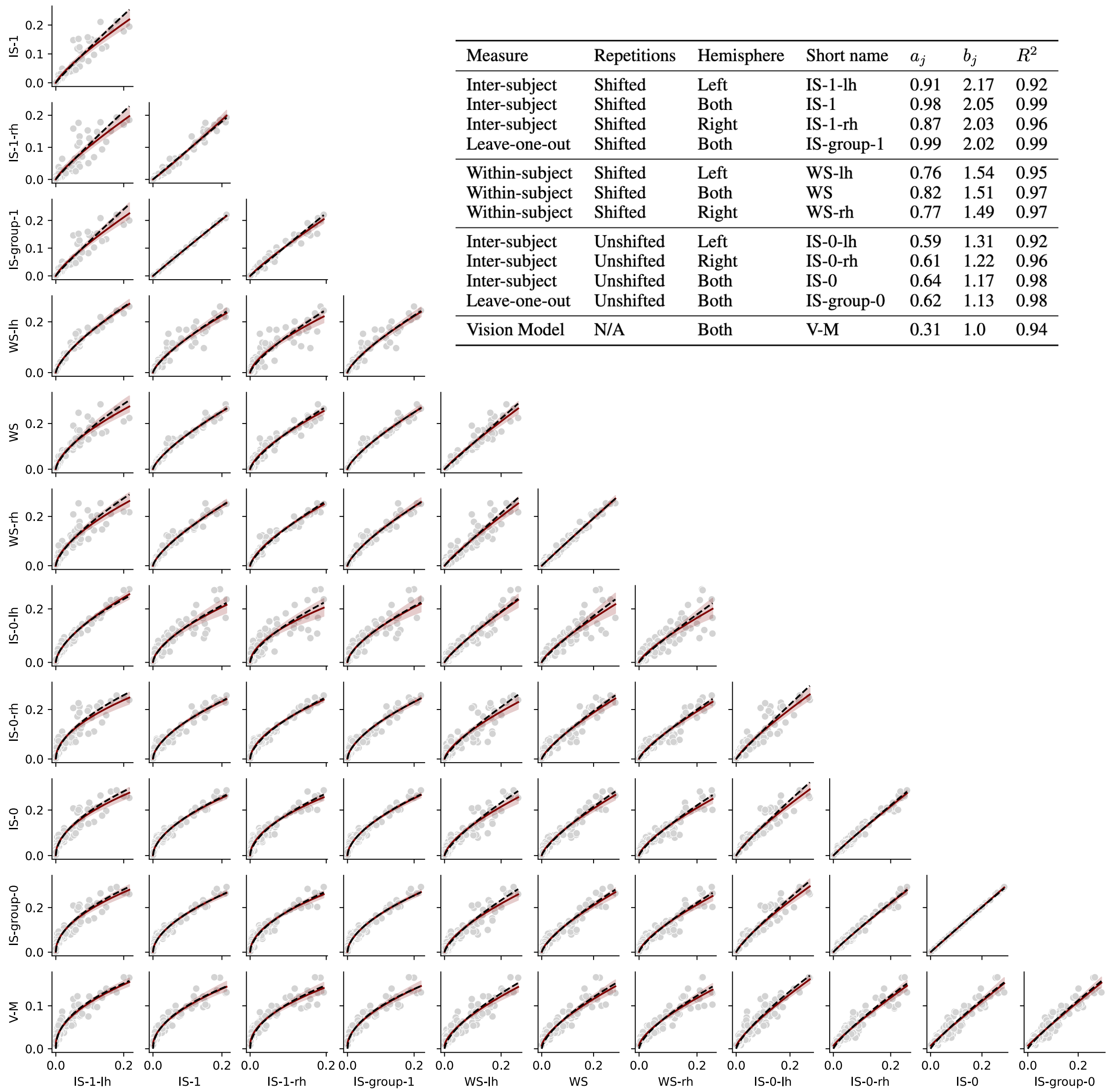

Measure	Repetitions	Hemisphere	Short name	a_j	b_j	R^2
Inter-subject	Shifted	Left	IS-1-lh	0.91	2.17	0.92
Inter-subject	Shifted	Both	IS-1	0.98	2.05	0.99
Inter-subject	Shifted	Right	IS-1-rh	0.87	2.03	0.96
Leave-one-out	Shifted	Both	IS-group-1	0.99	2.02	0.99
Within-subject	Shifted	Left	WS-lh	0.76	1.54	0.95
Within-subject	Shifted	Both	WS	0.82	1.51	0.97
Within-subject	Shifted	Right	WS-rh	0.77	1.49	0.97
Inter-subject	Unshifted	Left	IS-0-lh	0.59	1.31	0.92
Inter-subject	Unshifted	Right	IS-0-rh	0.61	1.22	0.96
Inter-subject	Unshifted	Both	IS-0	0.64	1.17	0.98
Leave-one-out	Unshifted	Both	IS-group-0	0.62	1.13	0.98
Vision Model	N/A	Both	V-M	0.31	1.0	0.94

Supplementary Figure S14: **Global power-law model captures pairwise relations among all RSA metrics.** Scatter plots (gray points) show parcel-wise observed RSA values for all metric combinations: inter-subject (shifted and unshifted), within-subject, subject-versus-group, and vision-model correlations, each additionally split by hemisphere sampling (left, right, both). Red curves depict an independent power-law fit for that specific scatter, whereas black dashed curves are the predictions from a single joint fit of Eq. 2 applied to all metrics simultaneously (no panel-specific re-fitting). The accompanying table reports the fitted scale factors a_j, exponents b_j, and explained variance R^2 for every metric (model-predicted vs observed RSA). All metric trends are explained with $R^2 > 0.92$, confirming that a common latent shared-geometry map, together with metric-specific scale and attenuation parameters, accounts for the full measurement set.

Supplementary Note 5: Token-occurrence control for language–brain RSA

To quantify how much of the language–brain alignment reflects initial tokenization, we constructed RDMs from token-occurrence vectors. Each caption was encoded as a count vector—each entry recording the number of times a given token appears—and pairwise Pearson dissimilarities among these vectors yielded a tokenizer-based RDM. We applied this procedure to six vocabularies (BLOOMZ, Gemma-2, LLaMA-1/2/3; see main text Table 1), covering all tokenizers used in our language models, and then computed parcel-wise RSA against NSD fMRI responses using the symmetric HCP atlas. Parcel-wise alignments were nearly identical across tokenizers (pairwise Spearman's $\rho \geq 0.978$), so we report their average.

The tokenizer–brain alignment map closely matches that of full language models: it peaks in the LOTC hub and shows minimal or negative alignment in early visual parcels (Suppl. Fig. S15A,D). Token-count geometry correlates strongly with each model's first-layer alignment (Pearson's $\rho = 0.78$; Suppl. Fig. S15B) and with maximal alignment across layers ($\rho = 0.68$; Suppl. Fig. S15C), indicating that token frequencies account for a substantial portion of the language–brain correspondence.

Repeating the analysis with a word tokenizer—splitting captions on whitespace rather than into subword units—again yielded pronounced LOTC alignment (Suppl. Fig. S15E). These findings indicate that a large fraction of LOTC alignment to language models in the present dataset is accounted for by lexical token content shared across descriptions, consistent with a "bag-of-words"-like account and with limited evidence that progressively composed syntactic representations are required to explain the observed correspondence. This view is also consistent with earlier observations that high-level visual cortex can align with semantic similarity spaces derived from relatively simple lexical-semantic models, including WordNet-based analyses in occipitotemporal cortex and semantic category maps spanning visual and non-visual cortex[4,5].

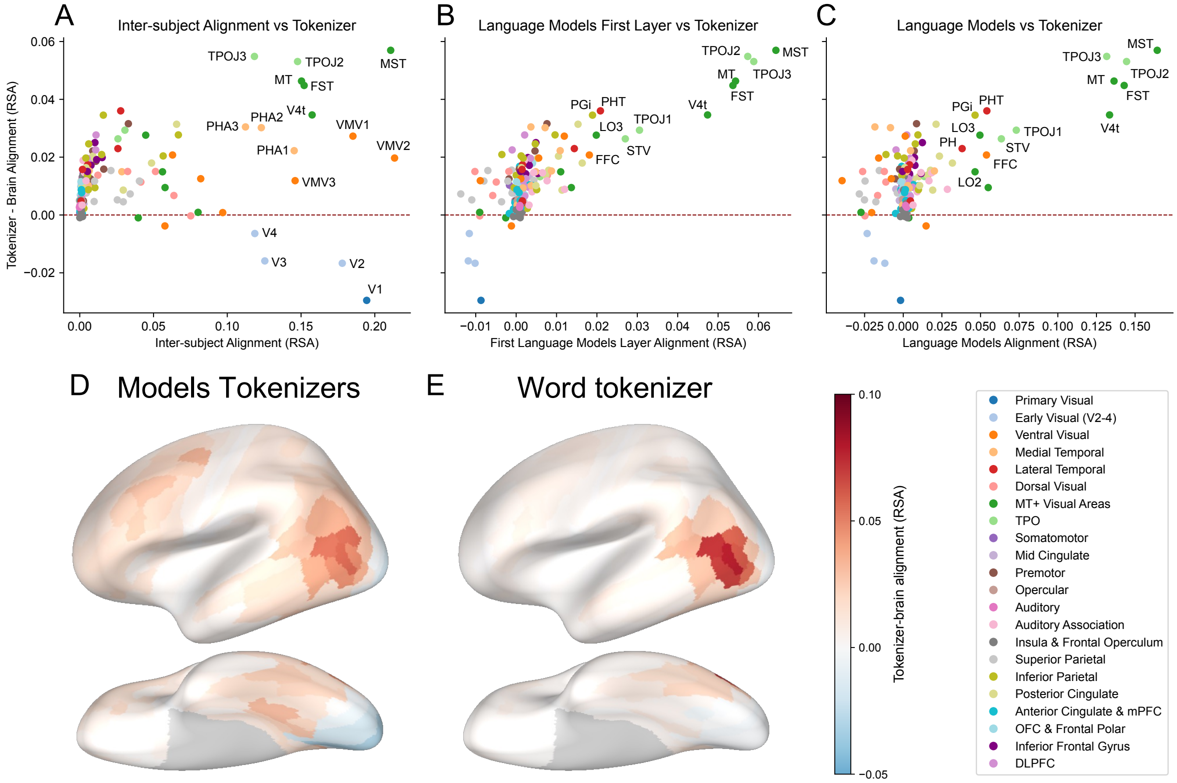

Supplementary Figure S15: **Tokenizer-level contributions to language–brain alignment.** We derived RDMs from token-occurrence count vectors over each model's vocabulary and computed parcel-wise RSA against NSD fMRI responses (averaged across the six tokenizers used in our language models). **(A)** Scatter of tokenizer-brain vs. inter-subject alignment, with peak values in the LOTC hub. **(B)** Token-count alignment against each model's first-layer alignment. **(C)** Token-count alignment against each model's maximal alignment across layers. **(D)** Cortical map of tokenizer-brain alignment, showing pronounced LOTC localization. **(E)** Alignment map for a whitespace-based word tokenizer.

Supplementary Note 6: Robustness to Similarity Metric (Spearman-RSA & CKA)

Recent work has cautioned that representational similarity findings can depend not only on stimulus design but also on the choice of similarity metric—some measures probe local, pairwise geometry while others emphasize global, space-wide dependence[6]. To test the robustness of our results to these methodological choices, we repeated key analyses using two alternatives to Pearson-RSA: (i) rank-based Spearman-RSA, which preserves only the ordering of pairwise dissimilarities, and (ii) Centered Kernel Alignment (CKA), which quantifies global statistical dependence via an unbiased Hilbert-Schmidt Independence Criterion (HSIC) estimator[7].

Replacing Pearson with Spearman correlation yields a purely rank-based, nonparametric measure agnostic to absolute distance magnitudes. As shown in Supplementary Fig. S16A–C, the inter-subject map, the parcel-by-parcel scatter against model alignment, and the representational connectivity matrix all mirror our original Pearson-RSA results (parcel-wise Spearman's $\rho = 0.99$).

CKA measures dependence across entire representational spaces, prioritizing alignment along principal axes rather than isolated pairwise distances. We computed linear-kernel CKA (using the unbiased HSIC estimator[7]) under the same shifted-repetition protocol. Supplementary Fig. S16D-F shows that CKA again recovers the three hubs but inflates LOTC alignment relative to Early Visual cortex in the parcel scatter. This reflects CKA's known sensitivity to low-dimensional, dominant components (often stronger in higher-level cortex) over the high-dimensional, finer-grained structure of early vision. Despite this spectral re-weighting, parcel-wise CKA and Pearson-RSA correlate strongly ($r = 0.91$), and the CKA connectivity matrix preserves the core two-stream topology, even though CKA is strictly non-negative and thus cannot index anticorrelated geometries.

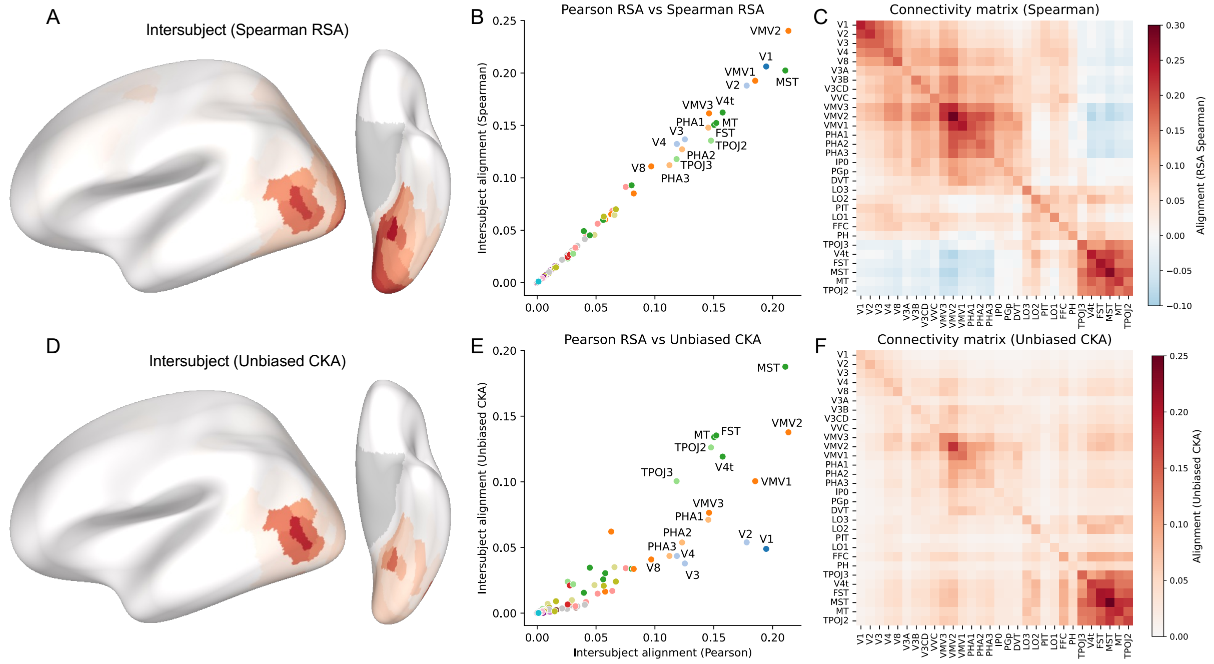

Supplementary Figure S16: **Comparison of alignment metrics. (A–C) Spearman-RSA.** Replacing Pearson's r with Spearman's rank correlation yields virtually identical maps: the inter-subject alignment (A), the parcel-wise scatter against vision- and language-model alignment (B), and the representational connectivity matrix (C) all match the Pearson-RSA findings, with parcel-wise strengths correlating at $r = 0.99$. **(D–F) Unbiased CKA.** Centered Kernel Alignment, which captures global dependence via an unbiased HSIC estimator, again highlights Early Visual, Ventral, and LOTC hubs (D). In the parcel-wise comparison (E), CKA inflates LOTC alignment and attenuates early visual alignment–reflecting its sensitivity to low-dimensional, dominant components–yet CKA and Pearson-RSA remain strongly correlated ($r = 0.91$). The CKA-based connectivity matrix (F) preserves the two-stream topology, though as a dependence measure it does not distinguish signed similarity.

Supplementary Note 7: Shared Component Decomposition and Partial RSA Controls

This supplementary note details our use of Kernel Multi-view Canonical Correlation Analysis (KMCCA)[8] to isolate the axes driving our RSA findings. While RSA provides a single global similarity score, KMCCA decomposes this correspondence into a ranked set of orthogonal "representational channels," revealing which latent axes contribute most strongly to the shared geometry.

We performed KMCCA using the Python package *mvlearn* (v0.5.0)[9] with a correlation kernel and a regularization parameter of $\lambda = 0.3$, utilizing the implementation's default parameters. For each cortical hub, we formed a data matrix for each participant from the GLM-denoised single-trial β-estimates. We included only those trials for which each (image, repetition) pair was available for all eight participants. This procedure yielded a set of eight matrices ($X_{p,r} \in \mathbb{R}^{m \times v_{p,r}}$), one for each participant, all representing an identical sequence of stimulus trials. The KMCCA algorithm was then applied to this set of matrices, returning projections onto a common shared subspace. For visualization (Supplementary Fig. S17A; Fig. 5A-C), we projected each subject's data onto the first two canonical axes and then averaged the projections for the repetitions of each unique image to produce a single, stable point per stimulus.

The parcel-level KMCCA projections (Supplementary Fig. S17A) confirmed that the organizational principles observed at the hub level were consistent within their constituent parcels. In Early Visual Cortex, the V1 parcel showed no clear semantic organization in its first two dimensions, whereas the V4 parcel already exhibited emergent clustering by image content. Within the Ventral hub, representative parcels such as VMV2 and PHA2 reproduced the hub-level scene-to-object gradient and showed clear organization by semantic category. Similarly, within the LOTC hub, parcels like MT and TPOJ2 showed the same primary separation between biological and non-biological stimuli. Within these main clusters, stimuli were further organized by fine-grained semantic categories, mirroring the main hub-level analysis (Fig. 5C).

To interpret these KMCCA axes semantically, we projected the image captions onto the shared subspace learned for each hub. We first created a vocabulary from the union of all words present in the captions ($s = 11,090$ unique words), encoding each caption as a binary word-occurrence vector (a "bag-of-words" representation). We then projected this caption matrix onto the first KMCCA component derived for each hub, yielding a loading weight for each word in the vocabulary. By ranking these weights, we identified the words that contributed most positively and negatively to

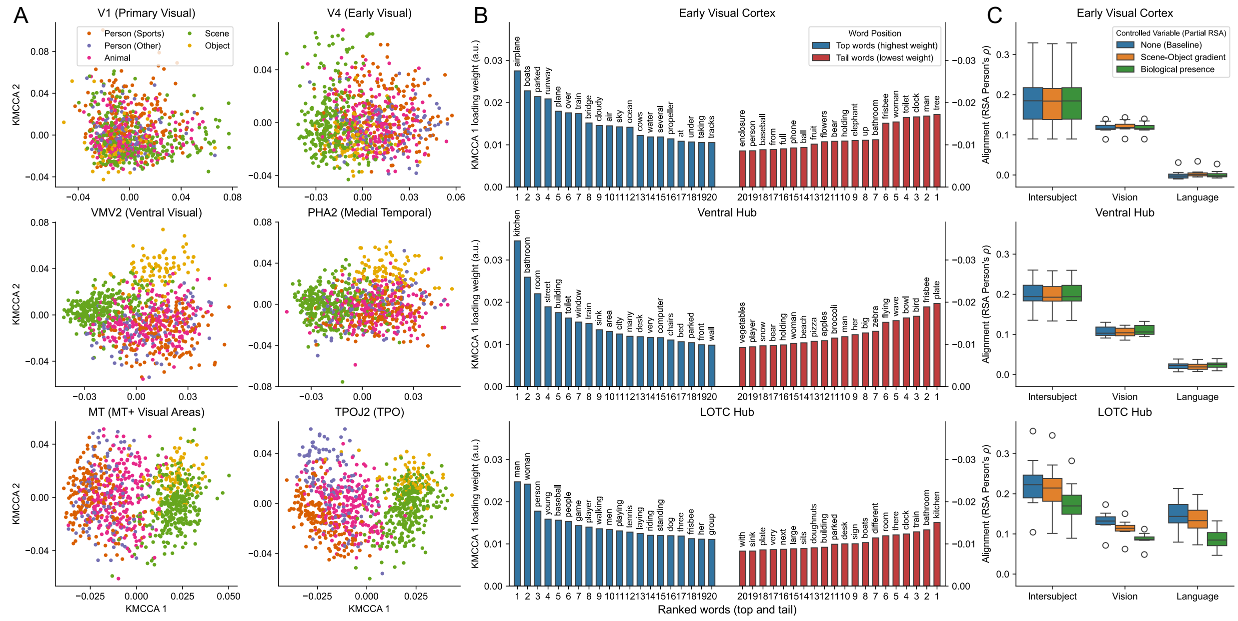

Supplementary Figure S17: **Extended analysis of representational dimensions. (A)** KMCCA projections onto the top two canonical axes for six representative parcels: V1, V4, VMV2, PHA2, MT, and TPOJ2. V1 shows no clear clustering; V4 begins to separate by content; Ventral and LOTC parcels replicate the hub-level semantic gradients. **(B)** Words ranked by loading on the first KMCCA component (positive loadings in blue, negative in red) for each hub. Early Visual words share low-level shape features; Ventral words span scene vs. object terms; LOTC words distinguish animate vs. inanimate labels. **(C)** Partial RSA boxplots for the three hubs, showing inter-subject alignment under no control (baseline), controlling for the scene-object gradient, or biological presence. LOTC alignment is markedly reduced by each control, whereas Early Visual and Ventral hubs are minimally affected.

each dimension (Suppl. Fig. S17B). This word-loading analysis confirmed our interpretation of each hub's organizing principle: in Early Visual Cortex, top-weighted words were not semantically coherent but appeared to share visual features; in the Ventral hub, positive tokens index scene contexts (e.g., 'kitchen', 'bathroom') while negative tokens name isolated objects (e.g., 'plate', 'frisbee'); and in the LOTC hub, tokens cleanly separated animate from inanimate labels.

Finally, we quantified the impact of the identified dimensions on the overall inter-subject alignment using partial RSA. Denoting $\rho(X, Y)$ as the standard Pearson correlation used to compute the alignment between two RDMs, and Z as a control RDM (e.g., one built from the first KMCCA component or from a binary "biological presence" vector), the partial RSA was computed as:

$$\mathrm{pRSA}(X, Y \mid Z) = \frac{\rho(X, Y) \; - \; \rho(X, Z)\, \rho(Y, Z)}{\sqrt{(1 - \rho(X, Z)^2)\,(1 - \rho(Y, Z)^2)}}. \tag{3}$$

As shown in the main text (Fig. 5d-f), controlling for the continuous, data-driven KMCCA axis produced only minimal drops in the Early Visual and Ventral hubs, but a substantial reduction in the LOTC hub. A complementary analysis confirmed this result: controlling for the discrete categorical variables yielded a drop in alignment of a similar magnitude to that produced by the continuous KMCCA components (Supplementary Fig. S17C).

Supplementary Note 8: THINGS-fMRI and BOLD5000 Processing

THINGS-fMRI dataset

We processed the THINGS-fMRI dataset[10] using a pipeline analogous to that for NSD. This dataset consists of 3T fMRI recordings from three participants performing an oddball detection task on 8,740 unique object images. A subset of 100 images was repeated once per session (12 presentations total); all other images appeared only once. For our primary computations, we used only the first presentation of each of the 8,740 images.

Pre-processed, ICA-regressed single-trial estimates were obtained from the authors' public repository. We extracted voxel responses into the HCP-MMP atlas parcels provided by the THINGS team.

To control for within-session variability, inter-subject alignment was computed on a session-wise basis. For any pair of participants (p, q) and parcels (r, r'), we computed IS-RSA by correlating the RDMs generated from the images within a given session s:

$$\mathcal{A}^{\mathrm{IS}}_{p,q,r,r',s} = \mathrm{RSA}\big(X_{p,r,s}, X_{q,r',s}\big). \tag{4}$$

Group-level maps were then obtained by averaging these scores across sessions and all participant pairs. Model-brain RSA followed the same session-wise procedure. As a control, we confirmed that computing IS-RSA over the full 8,740-trial set (using $8,740 \times 8,740$ RDMs) yielded parcel-wise results that were almost perfectly correlated with our session-wise computation (Pearson's $r = 0.995$). While the full-set computation produced systematically lower alignment values (linear slope ≈ 0.53; $R^2 = 0.99$), this correlation validates that our session-wise approach preserves the relative spatial pattern of the findings.

BOLD5000 dataset

We processed the BOLD5000 dataset[11] using the same session-wise pipeline as for THINGS-fMRI. This dataset consists of 3T recordings from four participants performing a valence rating task on 4,916 unique images drawn from the MS-COCO, ImageNet, and SUN databases. We used the pre-processed, single-trial β-estimates provided by the authors, selecting only the first presentation of each image. Inter-subject and model-brain RSA were computed following the same session-wise procedure described above.

For voxel extraction, we registered the HCP-MMP1.0 atlas from MNI152 standard space to each subject's native T1-weighted scan and then to their mean functional volume using FSL's FLIRT and FNIRT. Qualitative inspection confirmed consistent parcel definitions across participants. All other preprocessing and computational steps paralleled the main NSD pipeline.

Supplementary Note 9: Implementation details for large-scale RSA comparisons

To efficiently handle the computational demands of large-scale RSA analyses–driven by dataset size and extensive permutation testing–we developed a GPU-accelerated implementation using PyTorch. Specifically, we reformulated representational dissimilarity matrix (RDM) calculations as optimized batch matrix operations. For each participant pair $p, q \in \mathcal{P}$ and cortical region $r \in \mathcal{R}$, we constructed stacked matrices containing flattened RDMs for all regions simultaneously, significantly reducing memory overhead and runtime compared to iterative loop-based implementations.

Given data matrices for matched trials $X_{p,r}[\boldsymbol{i}_{pq}, :]$ and $X_{q,r'}[\boldsymbol{j}_{pq}, :]$ (where the number of matched trials is N_{trials}), we first computed the upper-triangular vectors of dissimilarities:

$$\mathbf{x}_{pq,r}^{(p)} = \text{vec}_u\, D\left(X_{p,r}[\boldsymbol{i}_{pq}, :]\right), \quad \mathbf{x}_{pq,r'}^{(q)} = \text{vec}_u\, D\left(X_{q,r'}[\boldsymbol{j}_{pq}, :]\right), \tag{5}$$

where $D(\cdot)$ denotes the dissimilarity function (e.g., $1 -$ Pearson r) and vec_u denotes vectorization of the upper triangle.

Each RDM vector was centered and l_2-normalized before being stacked into a single matrix containing all ROIs:

$$\mathbf{X}_{pq}^{(p)} = \begin{bmatrix} \tilde{\mathbf{x}}_{pq,1}^{(p)} \\ \tilde{\mathbf{x}}_{pq,2}^{(p)} \\ \vdots \\ \tilde{\mathbf{x}}_{pq,|\mathcal{R}|}^{(p)} \end{bmatrix} \in \mathbb{R}^{|\mathcal{R}| \times \frac{N_{\text{trials}}(N_{\text{trials}}-1)}{2}}, \tag{6}$$

and similarly for $\mathbf{X}_{pq}^{(q)}$.

The full inter-subject connectivity matrix was then obtained efficiently via a single matrix multiplication:

$$\mathcal{A}_{p,q}^{\text{IS}} = \mathbf{X}_{pq}^{(p)} \left(\mathbf{X}_{pq}^{(q)}\right)^{\top} \in \mathbb{R}^{|\mathcal{R}| \times |\mathcal{R}|}. \tag{7}$$

Analogous formulations were applied for Spearman-based RSA, with rank-transformation applied to RDM vectors prior to normalization and dot products.

For brain–model alignment, we constructed similar stacked tensors for brain and model RDMs across sessions. The brain tensor was organized as:

$$\mathbf{X}_{p}^{(\text{brain})} \in \mathbb{R}^{|\mathcal{R}| \times S \times \frac{N_{\text{trials}}(N_{\text{trials}}-1)}{2}}, \tag{8}$$

and the model tensor as:

$$\mathbf{X}_{m}^{(\text{model})} \in \mathbb{R}^{L \times S \times \frac{N_{\text{trials}}(N_{\text{trials}}-1)}{2}}, \tag{9}$$

where S is the number of sessions and L is the number of model layers.

Brain–model RSA alignments across all regions and layers were computed via a batched contraction of the normalized RDM vectors:

$$\mathcal{C}_{p,m} = \langle \mathbf{X}_{p}^{(\text{brain})},\ \mathbf{X}_{m}^{(\text{model})} \rangle. \tag{10}$$

This operation was implemented as an Einstein summation corresponding to the signature $r\,s\,d, l\,s\,d \to r\,l$ (summing over sessions s and dissimilarity pairs d).

For permutation testing, we permuted stimulus labels according to $\sigma \in S_{N_{\text{trials}}}$ prior to RSA computation. Rather than recomputing dissimilarity matrices from raw data after each shuffle (an $O(N_{\text{trials}}^2)$ operation), we pre-calculated the equivalent permutation σ' over the vectorized upper-triangular indices that matches the induced pairwise index shifts. This allowed us to permute the flattened RDMs directly via:

$$\mathbf{X}_{pq}^{(p)}[:, \sigma'], \tag{11}$$

preserving the correct stimulus-pair structure with minimal computational overhead.

Supplementary References

[1] Matthew F. Glasser, Timothy S. Coalson, Emma C. Robinson, Carl D. Hacker, John Harwell, Essa Yacoub, Kamil Ugurbil, Jesper Andersson, Christian F. Beckmann, Mark Jenkinson, Stephen M. Smith, and David C. Van Essen. A multi-modal parcellation of human cerebral cortex. *Nature*, 536(7615):171–178, 2016. doi:10.1038/nature18933.

[2] Chu-Chung Huang, Edmund T. Rolls, Jianfeng Feng, and Ching-Po Lin. An extended Human Connectome Project multimodal parcellation atlas of the human cortex and subcortical areas. *Brain Structure and Function*, 227(3): 763–778, 2022. doi:10.1007/s00429-021-02421-6.

[3] Emily J. Allen, Ghislain St-Yves, Yihan Wu, Jesse L. Breedlove, Jacob S. Prince, Logan T. Dowdle, Matthias Nau, Brad Caron, Franco Pestilli, Ian Charest, J. Benjamin Hutchinson, Thomas Naselaris, and Kendrick Kay. A massive 7t fMRI dataset to bridge cognitive neuroscience and artificial intelligence. *Nature Neuroscience*, 25(1): 116–126, 2022. doi:10.1038/s41593-021-00962-x.

[4] Thomas A. Carlson, Ryan A. Simmons, Nikolaus Kriegeskorte, and L. Robert Slevc. The emergence of semantic meaning in the ventral temporal pathway. *Journal of Cognitive Neuroscience*, 26(1):120–131, 2014. doi:10.1162/jocn_a_00458.

[5] Alexander G. Huth, Shinji Nishimoto, An T. Vu, and Jack L. Gallant. A continuous semantic space describes the representation of thousands of object and action categories across the human brain. *Neuron*, 76(6):1210–1224, 2012. doi:10.1016/j.neuron.2012.10.014.

[6] Marin Dujmovic, Jeffrey Bowers, Federico Adolfi, and Gaurav Malhotra. Inferring DNN-Brain alignment using representational similarity analyses can be problematic. In *ICLR Workshop on Re-Aligning Vision and Language Models with Human Values*, 2024.

[7] Le Song, Alex Smola, Arthur Gretton, Justin Bedo, and Karsten Borgwardt. Feature selection via dependence maximization. *Journal of Machine Learning Research*, 13(47):1393–1434, 2012.

[8] David R. Hardoon, Sandor Szedmak, and John Shawe-Taylor. Canonical correlation analysis: An overview with application to learning methods. *Neural Computation*, 16(12):2639–2664, 2004. doi:10.1162/0899766042321814.

[9] Ronan Perry, Gavin Mischler, Richard Guo, Theodore Lee, Alexander Chang, Arman Koul, Cameron Franz, Hugo Richard, Iain Carmichael, Pierre Ablin, Alexandre Gramfort, and Joshua T. Vogelstein. mvlearn: Multiview machine learning in python. *Journal of Machine Learning Research*, 22(109):1–7, 2021.

[10] Martin N. Hebart, Oliver Contier, Lina Teichmann, Adam H. Rockter, Charles Y. Zheng, Alexis Kidder, Anna Corriveau, Maryam Vaziri-Pashkam, and Chris I. Baker. THINGS-data, a multimodal collection of large-scale datasets for investigating object representations in human brain and behavior. *eLife*, 12:e82580, 2023. doi:10.7554/eLife.82580.

[11] Nadine Chang, John A. Pyles, Austin Marcus, Abhinav Gupta, Michael J. Tarr, and Elissa M. Aminoff. BOLD5000, a public fMRI dataset while viewing 5000 visual images. *Scientific Data*, 6(1):49, 2019. doi:10.1038/s41597-019-0052-3.
