# Supplementary material for: Shared representations in brains and models reveal a two-route cortical organization during scene perception
Source: Commun Biol. 2026 May 7;9:950. doi: 10.1038/s42003-026-10169-0 (PMC13365445; doi:10.1038/s42003-026-10169-0)
Supplement: Supplementary file 1 — Transparent Peer Review file [file 42003_2026_10169_MOESM1_ESM.pdf]

nature portfolio

Peer Review File

Shared representations in brains and models reveal a two-route cortical organization during scene perception

Corresponding Author: Mr Pablo Marcos-Manchón

This file contains all reviewer reports in order by version, followed by all author rebuttals in order by version.

Version 0:

Reviewer comments:

Reviewer #1

(Remarks to the Author)
In this work, Marcos-Manchon and Fuentemilla investigated the inter-subject convergence in stimulus representation and the nature of the information underlying this alignment, using vision and language models. Then, further analysis (hierarchal correspondence between models and brain, representational connectivity and content decomposition) provided more details on the functional organization of brain regions and the representation transition from sensory to high-level information.

In both the abstract and the introduction, it is mentioned that addressing the debate of external and internal constraints is the main goal but throughout the paper the ‘common representation’ seems to be a pre-assumption.
The Natural Scene Dataset, BOLD 5000 and THINGS are the datasets used which include fMRI responses for a large set of images recorded from 8, 4 and 3 participants, respectively. To study the representation convergence, wouldn’t it be more useful to use datasets with a large number of participants?
In Figure D1-B, the posterior cingulate shows larger correlation when compared with vision models, relative to inter-subject alignment. While visual, ventral and LOTC hubs have opposite trend. How is this explained/justified?
HCP parcels have different sizes when comparing regions, so it is important to keep parcel sizes in mind and some studies normalize results by surface area or voxel count.
The results in this research don’t show alignment between ventral regions and language models (e.g., Llama). However, Figure 1 in Doerig et al., (2025) shows the opposite, what could be the reasons for this difference?
“Doerig, A., Kietzmann, T. C., Allen, E., Wu, Y., Naselaris, T., Kay, K., & Charest, I. (2025). High-level visual representations in the human brain are aligned with large language models. Nature Machine Intelligence, 1-15.”
Comparing Figure 3 and 6, we can see clear differences in results for three datasets so generalization doesn’t seem available. For THINGS dataset alignments were confined to early visual cortex and for BOLD 5000, the early visual cortex show (significantly?) less alignment in comparison to the other datasets. It shows that based on experiments, we might not have all 3 hubs but when they are available, they show similar alignments, hierarchy, etc.
The caption of Fgure A1 refers to A1-E, but there is no panel E.
- On line 856, there is a typo: ‘KMCCCA’.

Reviewer #2

(Remarks to the Author)
Summary

This manuscript aims to characterize networks underlying visual perception in the human brain by comparing open datasets of image-fMRI response pairs with pre-trained deep neural networks (trained on either vision or language). The authors propose that sequentially applying existing methodologies (A: inter-subject similarity, B: model-brain similarity, C: inter-ROI similarity, D: interpretable dimensionality/component analysis) can yield a more systematic and complete evaluation of functional and hierarchical processes in the brain, as well as different (potentially more robust) results compared to prior findings.

Employing the Natural Scenes Dataset (NSD) for their main analysis, they outline that inter-subject similarity (A) can guide ROI filtering and selection of functionally-important regions (rather than for noise ceiling estimation, which is the common

practice in the field). Then those ROIs can be characterized in terms of their place in a low-to-high-level feature hierarchy using DNN model similarity (B). Jointly looking at inter-ROI similarity (C) along with (A) and (B), one can infer a potential connectivity structure by using (C) for the edge strength, (B) for the edge direction, and (A) for the node characterization in terms of functional region. On top of that, one can interpret the principal components/dimensions that give rise to this inter-subject functional specialization, using methods such as KMCCA (D).

Using (A), the authors identify three main hubs of processing: EVC, Ventral hub, and LOTC hub, and show using (B) that all three of them are captured by vision models but only the last one captured by language models, with even negative correlations of language models to the first two. Additionally, they show that inter-subject alignment exhibits a strong power-law relationship with vision-model-brain alignment, and that language models are even significantly better than vision models at capturing representations in the LOTC hub. Leveraging layer-wise alignment in (B), authors characterize the hierarchical signature of each hub, as low-level in EVC (<0.3), mixed mid-level (0.3-0.7) in Ventral, and high-level (>0.7) in LOTC. Combining the information of an ROI-similarity matrix from (C) into a connectivity network with weights from (C) and directions from (B), authors identify two distinct streams of visual processing; one medial-ventral, and one lateral-dorsal, both originating from EVC. Finally, dimension interpretability analysis in (D) reveals that the Ventral hub is more characterized by an Object-to-Scene gradient, while the LOTC hub by an Inanimate-Animate gradient, and that the latter is of such importance to LOTC that inter-subject alignment completely vanishes in the absence of animate content. Last, authors also cross-validate their findings in other datasets, namely BOLD5000 and THINGS-fMRI.

Overall impression

The methodology proposed is systematic and holistic, albeit not particularly novel in its parts. There is substantial merit, however, in this particular formulation of the methodology as a series of sequential steps (A-D, see above) to be applied not in isolation, but together. As for the novelty of the results, (A) yields a different functional specialization than what is commonly found in the literature, with no clear dorsal hub but separate hubs for ventral and lateral regions. The specific formulation as power-law connecting inter-subject to model-brain alignment is a fresh view on a known relationship (saturated relation of one following the other across ROIs, but model-brain alignment overall being lower). The negative correlation of language models to EVC and Ventral areas as well as the better alignment in LOTC has been partly reported in recent works contrasting models trained on vision-only, language-only, or models trained jointly on text and images, but effects here seem more distinct and larger - which is in a way surprising since many prior works also used the same fMRI dataset (NSD). The connectivity network uncovered compared to prior work reveals two streams partly different than the ones commonly suggested in the literature; these are a ventral and a lateral stream, instead of ventral, dorsal, and lateral; here the dorsal regions are more grouped together with ventral stream. The results in the dimensional analysis compared to prior work are mostly confirmatory.

Overall, we believe the work is convincing, with ample evidence to support the claims and sound methodology, as well as thorough statistical analyses, and code provided to reproduce the results, and detailed supplementary information. However, we believe the framing of the paper can be improved and some of the analyses be further fleshed out.

Specific comments and suggestions

Major comments:

1. Although there is novelty in the method and results, authors fail to make this novelty sufficiently clear. First, regarding the novelty of the method, the contrast with methods used so far in the literature and which gap the proposed method addresses is not motivated enough in the Introduction - the paper would greatly benefit from this in its ability to convince the reader. Second, regarding the novelty of the results, the paper lacks sufficient explicit contrasts to prior findings on the hubs, connectivity, and streams. Currently, statements are not consistent with the evidence presented - an example is the confirmatory statement “our representational connectivity analysis identifies a stimulus-driven network [..] consistent with the dual-network topology previously reported for scene perception [54]” while the presented evidence shows that the network identified is diverging from the standard dual-network topology. Also in the intro: “The other traces a lateral-dorsal route, selectively tuned to biological and social content, offering empirical support and a data-driven refinement for recent proposals of a ‘third visual pathway’ for social perception [29,30]”: the current results are only showing up lateral, not dorsal. The authors should more clearly delineate when results diverge from prior literature rather than stressing only commonalities. It is also important to comment on to what extent their language vs. vision model mappings diverge from recent literature by Wang et al., (2023) and Popham et al., (2021), who argue for more language-aligned representation in the higher ventral stream (in NSD) and on the ‘border’ of visual cortex, respectively.

2. The way the research question sets the scene is a bit unnecessary in the sense that it stresses a debate that the paper does not necessarily answer - the question of whether the architecture of the brain or the statistics of the outside world are more important is not addressed, as here the only focus is on the statistics of the outside. Currently, the Introduction sets up for an ‘ideosyncratic vs universal’ debate, but the evidence here is only on the universal, leaving the reader to expect evidence for the idiosyncratic side as well. Several somewhat abstract theoretical views/frameworks are invoked (Plato, Gibson), and questions are raised on the relation between brain anatomy and function, individual differences - these do not all seem particularly relevant to the current research question: it would be fine to motivate the paper just from the question as to how best identify networks involved in visual perception.

3. The description of the cross-validation findings in BOLD5000 do not exactly reflect the plots, and there are quite a few differences in the plots that are not commented on. Specifically, in 6a the LOTC hub has a “hole” in the middle and in 6b it

has a different shape as well, while EVC seems to be missing completely. In 6c, the layer progression of the Ventral hub is much less flat, showing a clearer peak than in NSD, while the layer progression of LOTC is conversely more flat than in NSD. In 6d, again EVC is missing and LOTC is less constrained to the ROIs that were found in NSD. Although these differences do not “break” the main claims, and also NSD is more reliable in scale and task, these should be commented on and not just overlooked with a mismatching description that “findings were replicated”.

4. The way connectivity is modeled (with direction going from low-level to high-level) does not allow for feedback, and so might produce an incorrect picture of information flow. Also, it is often the case that an ROI has less similarity with itself than with other ROIs; does that put into question the validity of that ROI / reproducibility of it across subjects? These aspects should be commented on more, as well as that similarity is only a proxy for connectivity (and should not be confused with anatomical connectivity, for example).

5. L218: “In Early Visual Cortex (Figs. 5a and D8), stimuli formed a category-free cloud organized primarily by low-level visual features“ this is not apparent in the figures. Also the figures show 2 components, while the text says “We focused our analysis on the first KMCCA component”.

6. L237: “These results showed that shared representational geometry is a robust framework for revealing the specific cortical networks where stimulus information is processed across both individuals and models”; this statement seems a bit circular. Can the authors comment on how much of the shared representational geometry could be driven or influenced by the quality of the measurement, i.e. the presence of a good fMRI signal in a given area, which is a prerequisite in order to capture brain activity with a model? (which is the logic behind noise ceiling computations).

7. The last two paragraphs of the Discussion (from L307 onwards) use a lot of impressive language, but lack concreteness. Exactly what is the envisioned ‘path for ‘hypothesis generation across sensory, cognitive, and cross-modal domains’? What ‘mechanistic understanding’ and ‘mechanistically testable hypotheses about information processing in the brain’ does this work yield? One issue here is that it’s not quite clear what the authors refer to with ‘our explainability analysis’ (see also comment below).

Minor comments

1. The use of past tense does not work well throughout the paper, e.g. “The directed graph, which highlights the network’s main connections, made the two-stream topology of information flow explicit “

2. It is stated that the model-brain alignment metrics are based on multiple models, but it’s not clear from figures 2E-G how the model dimension features in these analyses- are only the best models reported, or the average? Are the dots in E models, parcels, or subjects? Same for Fig 3.

3. Figure 5 “(E, F) Cortical maps of inter-subject RSA recomputed separately for those scenes without (E) versus with (F) biological agents (people or animals)” here E and F are flipped. Also in 5d, it is not clear that orange is with the 1st dimension removed in how it is phrased in the legend.

4. Figure 2b, a suggestion to keep orange as the color of Ventral hub for consistency

5. L 88: There are many different ways to define ‘scene statistics’; what is meant with the term here, exactly?

6. L272: ‘THINGS only engaged EVC’ - This seems too strong of a statement, and should be qualified with comparisons to previous papers that used THINGS - did they only find early visual cortex involved in object perception also?

7. It would be interesting to also see the directed graphs for the validation dataset (Fig. 6).

8. L277 mentions ‘explainability techniques’, but it’s not really clear what is meant here - the dimensionality analysis?

Textual comments:

- Line 30: “experiental” spelling error
- L 63 : ‘this cortical map’ - not clear what ‘this’ refers to
- L 66: not clear what ‘this prediction’ refers to
- L 69: the acronym HCP-MMP needs to be introduced
- L 71: ‘quantify the representational alignment across….’ [subjects]
- L 83: ‘to…’ categorization?
- Line 83: the sentence ending in “to40,41” is left unfinished
- Line 205 “Standard RSA”; specify “Standard IS-RSA”
- Line 242 “NSD picture dataset” weird phrasing
- L238 ‘was significantly reduced’ - what was reduced?

References:

Popham, S.F., Huth, A.G., Bilenko, N.Y. et al. Visual and linguistic semantic representations are aligned at the border of

human visual cortex. Nat Neurosci 24, 1628–1636 (2021). https://doi.org/10.1038/s41593-021-00921-6

Wang, A.Y., Kay, K., Naselaris, T. et al. Better models of human high-level visual cortex emerge from natural language supervision with a large and diverse dataset. Nat Mach Intell 5, 1415–1426 (2023). https://doi.org/10.1038/s42256-023-00753-y

Reviewer #3

(Remarks to the Author)
I co-reviewed this manuscript with one of the reviewers who provided the listed reports. This is part of the Communications Biology initiative to facilitate training in peer review and to provide appropriate recognition for Early Career Researchers who co-review manuscripts.

Version 1:

Reviewer comments:

Reviewer #1

(Remarks to the Author)
Dear,
Thank you for the opportunity to review the revised manuscript, Manuscript ID: COMMSBIO-25-7053-T.

I have read the response and the revised version. The authors have addressed the main concerns raised in the first round and I have no further questions or comments.

Thank you again for the invitation to review.
Best regards,
Elahe' Yargholi

Reviewer #2

(Remarks to the Author)
The revised manuscript has improved significantly. The structural reframing of the approach is helpful, and the additional comparisons with the other open datasets (BOLD5000 and THINGS) are now more comprehensive and convincing. It is also clear that this paper is a technical tour-the-force, packing a lot of interesting and detailed analysis into a single manuscript.

Nevertheless, a few concerns remain. Also, due to the quite significant overhaul of the main text (which was entirely rewritten, thereby inviting fresh review), some new questions emerged, which we detail below.

1.
One concern is that the novelty of the outcomes is (still) not sufficiently clear. Results sections 2.1 and 2.2. seem entirely confirmatory, except for line 115 where the negative correlation of pure-language models is shown. We believe there are novel insights in the paper, but they are not coming through clearly enough - we feel this is especially important to pay attention to since it is using the open dataset NSD, which has already been analysed by many researchers.

https://naturalscenesdataset.org/ maintains a list of papers that use NSD. Can the authors check the following:
- Are they the first NSD paper to directly contrast vision with pure language models, also in terms of layer depth alignment?
- Are they the first to derive a connectivity graph based on IS-RSA and populate it with VM/LM-RSA to derive hierarchies?

If so, clearly state this as a contribution of the paper. Here are some other specific insights that seem novel to us:

L119: "Unlike the next-token…" this seems to be providing a proposition for why the results deviate from what the literature has claimed so far, but it is phrased as if it is a result, rather than an explanation/novel insight. This should be more clear (e.g "we propose that…").

Same for L150 "under this input mismatch…' this seems to convey a really interesting, potentially novel insight on NSD model alignment, but the way it's formulated it's again a bit ambiguous if this is an empirical finding that is supported here, or a proposed explanation. And do we understand correctly that Supplementary Note 5 suggests that the language alignment is purely driven by token frequency? Isn't this a profound insight, arguing against a high-level abstract 'linguistic' representation in LOTC?

Also, among the vision models group here there are CLIP models, so how does it compare to the other, 'purely' vision models (i.e. do the authors reproduce the claim of Wang et al. (2023)?). In the rebuttal, the authors provide an explanation for why contrastive models like CLIP align more with the ventral stream, but it's not clear whether they find any distinction between the vision(-y) models themselves and how this relates to the poor language alignment in ventral but strong alignment in LOTC - we think this can be seen in Supplementary Figure 5 but it would be worth commenting on explicitly in the main text.

2.
The signal quality question, raised in our original review question #2.Q6, is not entirely resolved, in our opinion. Supplementary Note 2 now includes helpful comparisons of how IS-RSA scores of different brain parcels are affected by various resampling schemes of stimuli and repeats, whereby the authors conclude that ‘parcels with stronger shared geometry yield more stable IS-RSA scores regardless of their size’. However, the question we raised was not whether parcel size (n voxels) affected the IS-RSA scores, but rather whether the quality of the fMRI signal in a parcel predicts the degree of inter-subject alignment - for example via a direct assessment of the relation between voxel-wise SNR and IS-RSA score. Can the authors report a simple correlation between the average SNR in a parcel and its IS-RSA score? Hereby the metric of SNR should be independent of any model alignment (elsewhere, vision model alignment seems to be treated as a proxy for ‘signal strength’, e.g. response to 1Q3 - which may not be a valid assumption to make), e.g. something like temporal SNR (how strong is the response to an stimulus image relative to baseline/noise).

3.
The Discussion Section starting on line 262 could benefit from a more explicit discussion of why the classic ‘dorsal’ route is absent in the current findings. The authors note that their found organization ‘does not match classical ventral/dorsal’, but then say that this is because the results show ‘fractioning into additional routes (l269)’ without clearly pointing out that the classic dorsal route is in fact, not found in the current results. If one looks at activation-based intersubject synchrony as reported in classic papers by Uri Hasson, the dorsal pathway (e.g. TOS, POS, IPS) does show high alignment scores (e.g. Figure 1 in Hasson, et al., 2004, Science). So why do these dorsal ROIs not show up in the current analyses? Could this be due to the use of movies instead of images? Or to their results being based on simple correlations of fMRI activation time-courses, not representational geometries? Or does the shifted analysis introduced here make dorsal synchrony disappear - i.e. are dorsal regions more idiosyncratic across participants, or more driven by task structure than stimulus content? This also ties into our question about signal reliability (2) - if there is high tSNR in dorsal, but no shared representational geometry, that would provide strong evidence the latter is not directly coupled to the signal, and provide stronger support for the claims made on lines 326 (‘reliability alone cannot explain’) and 328 (‘rather than merely tracking’).

Hasson, U., Nir, Y., Levy, I., Fuhrmann, G., & Malach, R. (2004). Intersubject synchronization of cortical activity during natural vision. Science, 303(5664), 1634-1640.

Minor comments:

Line 89: underlay -> underlie
Line 109 ‘and more weakly in anterior regions of visual cortex’ - which visual hub is referred to here?
Line 148: ‘this contrast’ ambiguous what it refers to (vision vs. language model alignment?)
Line 295: the qualifier ‘coarse’ is repeated several times in the interpretation of the model-brain alignment, but why are the current results evidence for the alignment being based on 'coarse' dimensions? Could it not be a fine-grained mapping?
Line 298: ‘MT/LOTC showed alignment to specific stimulus categories’ > what is this inference based on? The drop in alignment when removing KMCCA 1 in Fig 5D?
Line 310: The interpretation here about ‘prediction and compression’ seems a bit far-fetched, none of the results presented here show any evidence for such processes happening in the brain. The paper provides evidence for emergence of common representational axes - yes. But the reason why is not clear, there could be many reasons for this not limited to prediction or compression.

Supplementary Note 4: The provided power-law attenuation is very interesting, but it was not clear to us how it is consistent with the idea that the VM alignment is less noise susceptible (response to R1Q3): why does a high latent rho result in a lower observed similarity compared to the other metrics (Supp Fig 16b)? It is indeed less non-linear power-law like, but also overall much lower than the WS and IS scores.

Rebuttal to #2Q4.2 It is good to observe that with the normalized version of this analysis, the same dual stream network is recovered - however there are some slight differences in connectivity between specific ROIs, so it might be more reliable for readers to view the normalized version (rather than reporting the normalized version only in the Supplement, as the authors decided to do now).

Version 2:

Reviewer comments:

Reviewer #2

(Remarks to the Author)
I thank the authors for their thoughtful engagement with the second round of reviews, after which I have no further comments. This study reports a very interesting and rich set of results, whilst also promoting an innovative, comprehensive RSA-based framework. Congratulations to the authors for their elaborate piece of work, I hope it will receive substantial attention upon publication.

Open Access This Peer Review File is licensed under a Creative Commons Attribution 4.0 International License, which permits use, sharing, adaptation, distribution and reproduction in any medium or format, as long as you give appropriate credit to the original author(s) and the source, provide a link to the Creative Commons license, and indicate if changes were made.
In cases where reviewers are anonymous, credit should be given to 'Anonymous Referee' and the source.
The images or other third party material in this Peer Review File are included in the article's Creative Commons license, unless indicated otherwise in a credit line to the material. If material is not included in the article's Creative Commons license and your intended use is not permitted by statutory regulation or exceeds the permitted use, you will need to obtain permission directly from the copyright holder.
To view a copy of this license, visit https://creativecommons.org/licenses/by/4.0/

Response to Reviewers

Manuscript ID: COMMSBIO-25-7053-T

Journal: *Communications Biology*

Original title: *Convergent transformations of visual representation in brains and models*

Revised title: *Shared representations in brains and models reveal a two-route cortical organization during scene perception*

Dear Editor and Reviewers,

We sincerely thank you for the thorough evaluation of our manuscript and for the constructive comments and suggestions.

In response to this feedback, we have performed a major revision of the manuscript centered on a structural reframing of our approach. Specifically, we have shifted the paper's narrative from a broad debate regarding the universality of representations to a concrete, results-oriented framework focused on cortical network identification. As part of this revision, we have substantially revised the Introduction, Results, and Discussion to align with this new focus. To reflect this sharper scope, we have updated the title of the manuscript to *Shared representations in brains and models reveal a two-route cortical organization during scene perception.*

Additionally, we have uploaded the derivative data for generating all figures to a public repository (10.6084/m9.figshare.30753239) and have updated our public code repository with the analysis scripts used for this revision.

In the attached revised manuscript, we have highlighted in blue those sections updated as part of the revision. Below we provide a point-by-point response, indicating how we have addressed each comment. In this document, we reproduce each reviewer comment (in blue) and provide our response immediately below.

Reviewer #1

Reviewer 1.Q1 In both the abstract and the introduction, it is mentioned that addressing the debate of external and internal constraints is the main goal but throughout the paper the 'common representation' seems to be a pre-assumption.

We appreciate this comment, which is closely related to Reviewer #2.Q2 comment. In the original manuscript, we attempted to situate the study within the broader debate on universal versus idiosyncratic constraints in neural representation. We agree that framing the paper around this theoretical dichotomy created an expectation of a comparative test that our methodology, which specifically focuses on shared representation, was not designed to resolve.

To address this, we have substantially revised the Abstract, Introduction, and Discussion to focus directly on the methodological and empirical contributions of the work: specifically, how shared representational geometry can be used to identify functional networks involved in visual processing. We now motivate the study from this perspective, as suggested by Reviewer #2, avoiding the implication that the paper aims to contribute to the universal/idiosyncratic debate.

In the revised manuscript, we treat inter-subject convergence as a methodological premise rather than a hypothesis. We now motivate this approach in the Introduction (L19-25) by citing evidence that stimulus-locked responses exhibit reliable synchrony, positioning inter-subject alignment as a tool to localize consistent functional regions and define the shared representational backbone.

Reviewer 1.Q2 The Natural Scene Dataset, BOLD 5000 and THINGS are the datasets used which include fMRI responses for a large set of images recorded from 8, 4 and 3 participants, respectively. To study the representation convergence, wouldn't it be more useful to use datasets with a large number of participants?

We appreciate the reviewer raising this point regarding sample size. We agree that a larger number of participants is generally desirable to estimate population-level convergence. However, our methodology, specifically IS-RSA and KMCCA, requires a large number of shared stimuli viewed by all participants to compute reliable pairwise alignments.

When selecting datasets, we faced a trade-off between participant count (N) and shared stimulus count (M). Most large-N datasets prioritize stimulus diversity over overlap (e.g., the Natural Object Dataset has $N = 30$, but overlaps on only 200 images for a subset of the participants). For representational geometry analyses, such limited overlap is insufficient to robustly populate the representational space.

Therefore, we prioritized datasets that maximize shared sampling: NSD (1,000 shared images $\times$ 3 repetitions), THINGS (8,740 shared), and BOLD5000 (4,916 shared). These datasets offer the necessary density to construct stable RDMs and perform component decomposition, which would be unreliable in sparsely sampled large-N cohorts.

To address this, we have added a clarification in the Methods section (L369–371) stating that dataset selection was guided by the requirement for extensive shared stimulus sampling.

Reviewer 1.Q3 In Figure D1-B, the posterior cingulate shows larger correlation when compared with vision models, relative to inter-subject alignment. While visual, ventral and LOTC hubs have opposite trend. How is this explained/justified?

We thank the reviewer for highlighting this feature of the data. In parcels such as the posterior cingulate cortex (PCC), vision model–brain RSA can indeed exceed inter-subject RSA (or even within-subject RSA; Rebuttal Fig. 1A). This apparent inversion is not a qualitative difference in PCC coding, but a consequence of the fact that these metrics operate under different effective noise regimes and therefore are not directly comparable in magnitude.

Different noise regimes (One-sided vs. Two-sided noise). Model–Brain RSA correlates a noisy fMRI signal with a noise-free model embedding (one-sided noise). In contrast, Inter-Subject RSA correlates two noisy biological signals (two-sided noise). As a result, measurement noise attenuates IS-RSA roughly quadratically compared to the linear attenuation of Model-Brain RSA (Rebuttal Fig. 1B). In high-signal regions (like LOTC), the strong shared signal overcomes this penalty. However, in low-signal regions (like PCC), the double-penalty of noise drives IS-RSA toward zero faster than model–brain RSA, causing the observed inversion.

Consistency with the cortex-wide scaling relationship. Across parcels, IS-RSA and vision-model alignment follow an approximate power-law scaling (Fig. 2e; Supplementary Note 4). PCC parcels lie on this same relationship but occupy its lower-magnitude range (Rebuttal Fig. 1B), consistent with the interpretation that the apparent inversion reflects differential attenuation rather than a qualitatively different phenomenon specific to PCC.

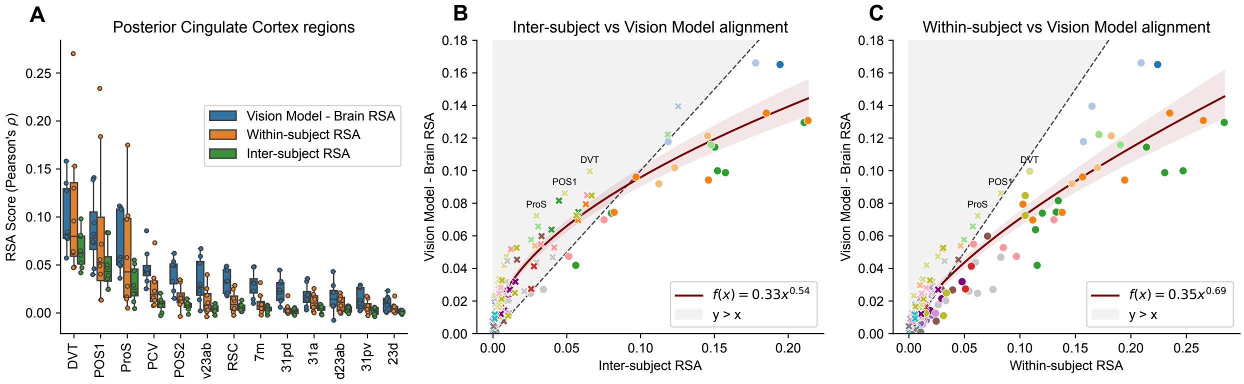

Rebuttal Figure 1: Signal scaling in the Posterior Cingulate Cortex (PCC). (A) RSA magnitudes for the vision model, within-subject RSA, and inter-subject RSA across PCC parcels. **(B–C)** Scatter plots showing PCC parcels (labeled) overlaid on the cortex-wide power-law fits (red lines) relating Inter-Subject RSA (**B**, $R^2 = 0.94$) and Within-Subject RSA (**C**, $R^2 = 0.89$) to vision model alignment. Regions where vision model alignment exceeds inter-subject/within-subject alignment are shaded in gray; parcels falling into this regime are marked with a cross.

Modeling the Power-Law Attenuation. As detailed in Supplementary Note 4, if we assume all metrics capture the same underlying representational content subject to metric-specific attenuation, the relationship between them follows a power law:

$$RSA_{\text{observed}} \approx a \cdot (RSA_{\text{latent}})^b \tag{1}$$

This model explains $R^2 = 0.94$ of the cortex-wide relationship between IS-RSA and vision model alignment. The observed sublinear relationship ($y \propto x^{0.54}$; Rebuttal Fig. 1B) confirms that IS-RSA is attenuated more steeply ($b_{\text{IS}} > b_{\text{model}}$) as signal strength decreases.

Conclusion for PCC. PCC parcels naturally exhibit weaker shared visual representational structure than early visual areas. When plotted against this cortex-wide scaling law (Rebuttal Fig. 1B-C), PCC parcels fall directly on the expected curve–they simply occupy its lower range. Thus, the observation that vision models outperform inter-subject alignment in the PCC is fully consistent with the global signal attenuation model.

We have clarified this in the Discussion (L313–329) and added explicit pointers to the formal derivation in Supplementary Note 4.

Reviewer 1.Q4 HCP parcels have different sizes when comparing regions, so it is important to keep parcel sizes in mind and some studies normalize results by surface area or voxel count.

We agree that parcel size differences could, in principle, affect the stability of RSA estimates. HCP parcels vary substantially in surface area and voxel count (e.g., under the NSD 1mm preprocessing, V1 ≈ 20,000 voxels vs. VMV2 ≈ 1,200 voxels), and smaller parcels could exhibit lower SNR, potentially leading to less stable IS-RSA estimates.

To evaluate whether parcel size materially impacts our results, we performed two complementary analyses targeting (1) estimation stability and (2) mean magnitude.

1. Reliability quantification via resampling. We examined whether parcel size influences RSA stability by bootstrapping two independent sources of variance:

(i) *Stimulus generalization* (Rebuttal Fig. 2A–D): We recomputed IS-RSA on 1,000 random half-splits of the stimulus set (500 images per split). This tests whether parcels of different sizes are differentially sensitive to variation in visual content.

(ii) *Measurement reliability* (Rebuttal Fig. 2E–H): We fixed the stimulus set and randomly sampled one repetition per image (from the available 2–3) across 1,000 iterations, testing sensitivity to trial-level noise.

Across both resampling analyses, the dispersion of IS-RSA estimates showed no systematic dependence on parcel voxel count (Rebuttal Fig. 2C,G). Instead, stability was governed primarily by the *strength* of the shared signal: relative dispersion (coefficient of variation) decreased as IS-RSA magnitude increased (Rebuttal Fig. 2D,H), regardless of parcel size.

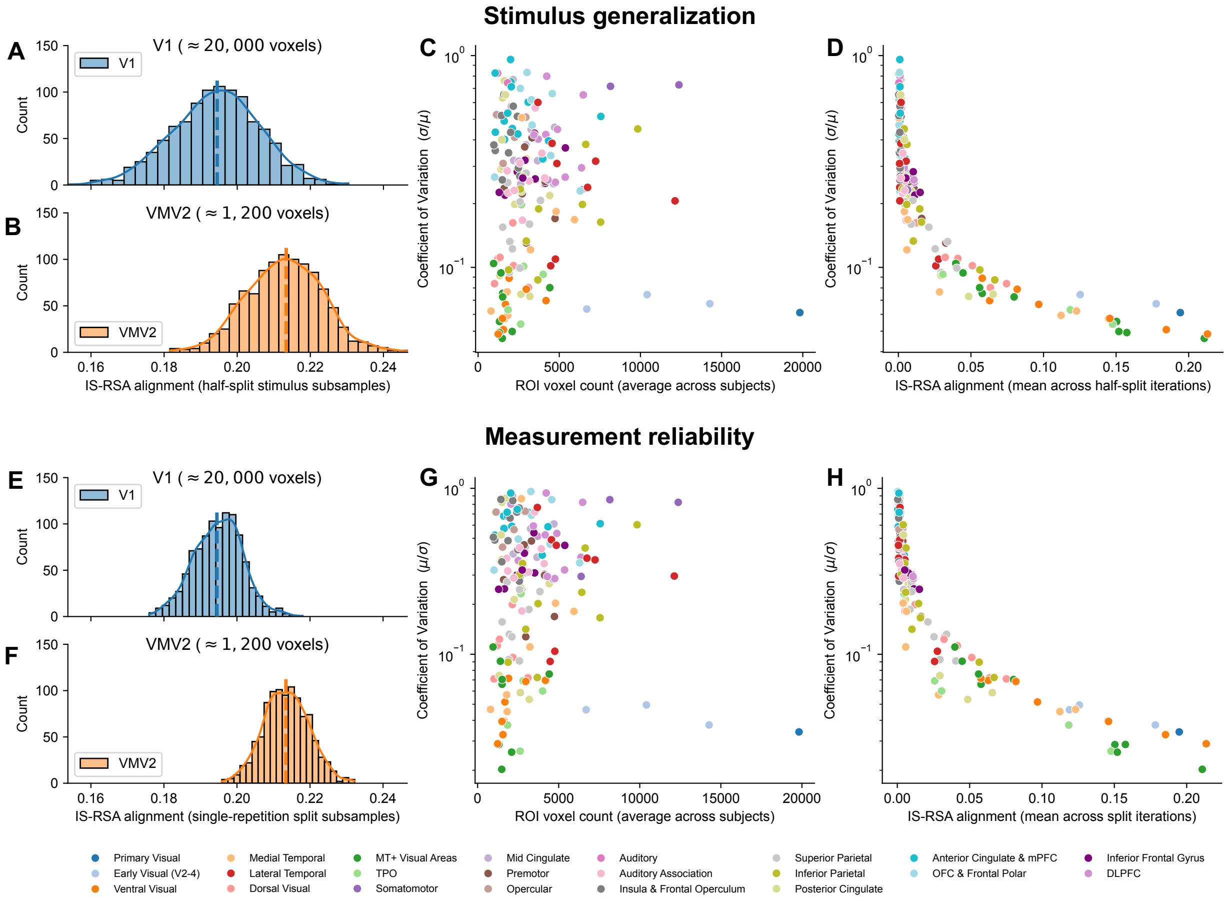

Rebuttal Figure 2: Quantifying parcel reliability via resampling. **(A–D)** Stimulus generalization analysis. We computed IS-RSA on 1,000 random half-splits of the stimulus set (selecting 500 stimuli in each subsample). **(A,B)** Distribution of IS-RSA scores for a representative large parcel (V1) and small parcel (VMV2). Dashed vertical lines indicate the IS-RSA estimate from the full dataset. **(C)** IS-RSA coefficient of variation (CV; std/mean) plotted against parcel voxel count. **(D)** CV plotted against mean IS-RSA alignment. **(E–H)** Measurement reliability analysis based on randomly selecting one repetition per image across 1,000 iterations. **(E,F)** Corresponding distributions for V1 and VMV2. **(G)** CV versus voxel count. **(H)** CV versus mean IS-RSA alignment. In both analyses, the dispersion shows no systematic dependence on voxel count but decreases as a function of the measured IS-RSA magnitude.

2. Statistical control using regression. To verify that parcel size does not drive the magnitude of the observed alignment, we fitted a linear mixed-effects model to regress out voxel count:

$$\text{IS-RSA} \sim \text{ROI} + \log(\text{voxel\_count}) + (1 \mid \text{subject}) \tag{2}$$

The effect of voxel count was not significant ($\beta = 0.007, p = 0.206$). Furthermore, the voxel-count–adjusted IS-RSA values were qualitatively identical to the uncorrected estimates (Rebuttal Fig. 3).

These results indicate that neither the magnitude nor the stability of IS-RSA is driven by parcel size in this dataset. We have added the resampling analysis as a new Supplementary Note 2 and cite it in the main text (L80-81).

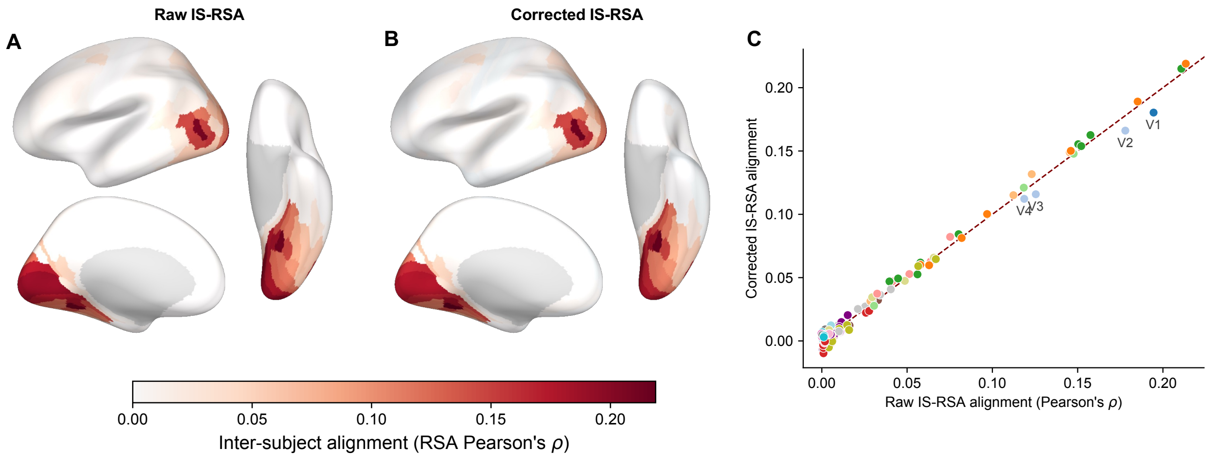

Rebuttal Figure 3: IS-RSA estimates after controlling for parcel size. (**A**) Uncorrected inter-subject representational similarity (IS-RSA) alignment across HCP parcels (identical to the results reported in the main text Fig. 2B). (**B**) Voxel count-corrected IS-RSA map. We fitted a linear mixed-effects model (IS-RSA $c(\text{ROI}) + log(\text{voxel count}) + (1|\text{subject})$) to quantify the contribution of parcel size to IS-RSA alignment. The map displays the model-adjusted values (residuals + global mean) after regressing out the effect of voxel count. (**C**) Scatterplot comparing raw vs. corrected IS-RSA values.

Reviewer 1.Q5 The results in this research don't show alignment between ventral regions and language models. However, Figure 1 in Doerig et al., (2025) shows the opposite, what could be the reasons for this difference?

We thank the reviewer for raising this important question. We examined this discrepancy in detail and found that it likely reflects three factors: (i) the specific architecture and objective of the language model evaluated in Doerig et al. (2025), (ii) methodological differences between searchlight-based and ROI-based RSA, and (iii) the focus on the final embedding layer versus layer-wise trajectories.

To address this, we evaluated the specific model used by Doerig et al. using our pipeline. Below we summarize the results.

Replication of MPNet ventral alignment. We evaluated the exact model used in Doerig et al. (*all-mpnet-base-v2*[1]) using our HCP-parcel RSA pipeline. Following their approach, we extracted final-layer sentence embeddings using the MS-COCO captions associated with each NSD image. The resulting alignment map (Rebuttal Fig. 4A) successfully reproduces the qualitative ventral alignment reported by Doerig et al., particularly in ventral/scene-related regions. Note that the absolute magnitude is lower in our ROI-based analysis ($r \approx 0.10$) than in their searchlight maps, likely reflecting the different spatial smoothing and signal averaging inherent to ROI vs. searchlight approaches.

Layer-wise profiles are consistent with our broader findings. Crucially, when we analyzed MPNet across all transformer blocks (Rebuttal Fig. 4B,C), we found that *intermediate* layers show strong correspondence in LOTC-adjacent regions, consistent with the LLMs in our main text. The distinct ventral signal primarily emerges in the final layers. When summarizing alignment by taking

[1]https://huggingface.co/sentence-transformers/all-mpnet-base-v2

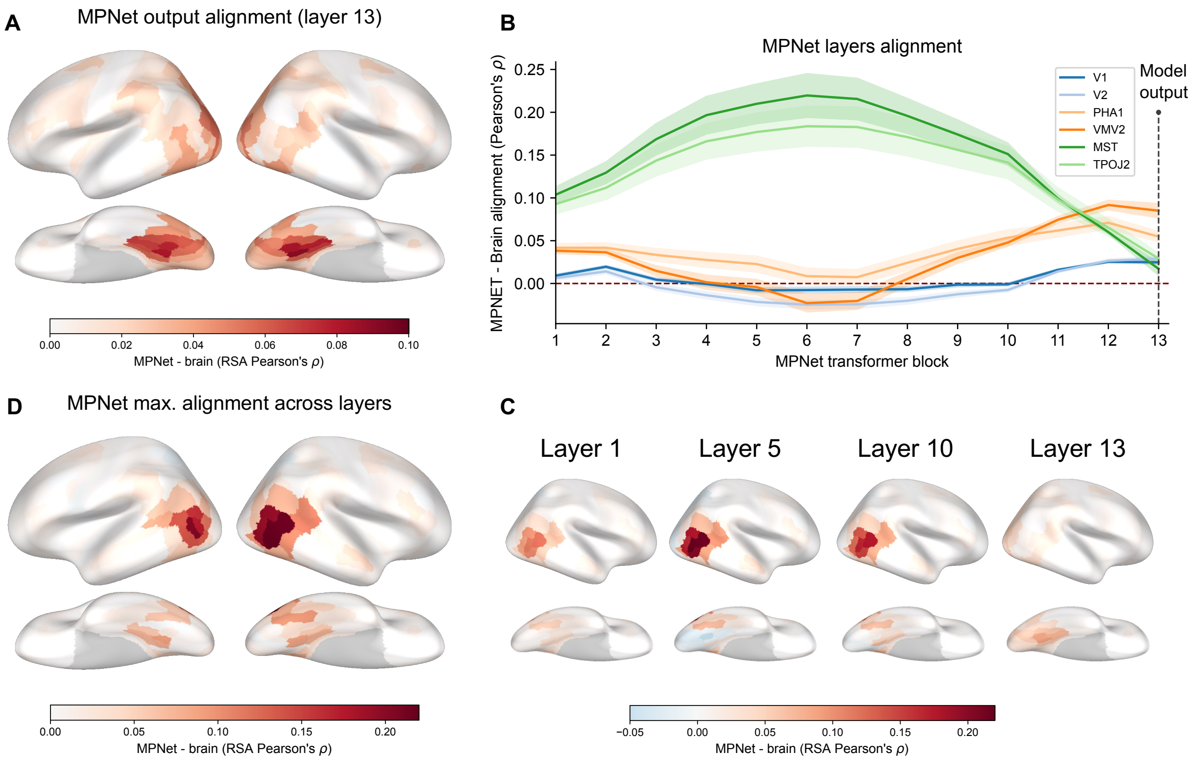

Rebuttal Figure 4: Alignment of MPNet model. (**A**) Alignment for the final output layer (Layer 13) of the MPNet model (*all-mpnet-base-v2*), computed using the ROI-based RSA pipeline. (**B**) Layer-wise alignment profiles for representative HCP parcels. The curves display the RSA score at the output of each transformer block (1–13) for early visual (V1, V2), ventral (PHA1, VMV2), and lateral-dorsal (MST, TPOJ2) regions (shaded areas denote SEM across 8 subjects). (**C**) Cortical surface maps of the right hemisphere showing model-brain alignment at layers 1, 5, 10, and the output layer (13). (**D**) Cortical map of the maximum alignment score achieved across all model layers for each region. All results display group-level alignment (average of 8 NSD subjects).

the maximum across layers (Rebuttal Fig. 4D), LOTC remains the region with the highest peak alignment, consistent with the spatial distribution observed for the autoregressive LLMs evaluated in the manuscript.

Why MPNet differs from Causal LLMs. The key driver of the difference in the final layer is likely the training objective. The models in our manuscript (Llama, Gemma, Bloomz) are causal LLMs trained on *next-token prediction*. This objective does not enforce that the final hidden state must form a compact semantic space where similar concepts cluster together. By contrast, MP-Net is a sentence transformer explicitly fine-tuned with a *contrastive objective* to maximize the similarity between semantically related sentences. Since NSD captions describe high-level scene semantics (which are known to structure ventral-stream representations), MPNet's output embedding naturally aligns with ventral areas.

In short, MPNet asks: *"Are these two captions about the same thing?"* (Semantic Similarity → Ventral alignment), whereas Causal LLMs ask: *"What word comes next?"*, generating a representational geometry that does not mirror this ventral organization.

We have added a clarification of this objective-dependent difference in the Results section when introducing the language models findings (L115-121).

Reviewer 1.Q6 Comparing Figure 3 and 6, we can see clear differences in results for three datasets so generalization doesn't seem available. For THINGS dataset alignments were confined to early visual cortex and for BOLD 5000, the early visual cortex show (significantly?) less alignment in comparison to the other datasets. It shows that based on experiments, we might not have all 3 hubs but when they are available, they show similar alignments, hierarchy, etc.

We appreciate this comment, which is closely related to the Reviewer #2.Q3 comment. We agree that the comparison section in the original manuscript did not adequately discuss the similarities and differences across NSD, BOLD5000, and THINGS. Our initial description was too compressed and did not explicitly address the dataset-specific deviations noted by the reviewer.

To improve this, we have substantially revised the cross-dataset comparison section (Section 2.6) to provide a rigorous evaluation of which aspects of the organization generalize and which are dataset-dependent.

In the revised text, we clarify that differences in stimulus sets and task regimes are key determinants of which cortical networks show detectable alignment:

- **BOLD5000:** Alignment preserves the ventral (VMV/PHA) and LOTC hubs, consistent with the presence of rich scenes and social interactions. However, early visual cortex alignment is comparatively weak, likely reflecting the shorter stimulus duration and lower trial-level reliability relative to NSD.
- **THINGS-fMRI:** Early visual alignment forms a clear cluster, but the ventromedial/parahippocampal hub and the separable LOTC-centered route are not detected. This is consistent with THINGS

being dominated by object-centric images on white backgrounds, with limited scene layout and minimal social interaction content.

Overall, we agree with the reviewer's interpretation: the full three-hub/two-route organization is not always expressed across every dataset. However, when specific hubs are detectable, their alignment profiles and hierarchical trends are qualitatively consistent. We interpret the reduced or missing hubs in certain datasets as a consequence of stimulus content and task demands.

We have revised the manuscript accordingly, including a rewritten Comparison section (Section 2.6; L220–256) that explicitly discusses these convergences and differences.

Reviewer 1.Q7 The caption of Figure A1 refers to A1-E, but there is no panel E.

We thank the reviewer for spotting this error. The supplementary figure included in the original manuscript was an outdated version that inadvertently omitted panel E. We have updated the figure to the correct version, which is now included as Supplementary Fig. 15 within Supplementary Note 3 (page 37 of the revised manuscript). For reference, the updated figure is included at the end of this document (Rebuttal Fig. 6).

Reviewer 1.Q8 On line 856, there is a typo: 'KMCCCA'.

We thank the reviewer for catching this typo. We have corrected it in the revised manuscript.

Reviewer #2

Major comments

Reviewer #2.Q1 **(1)** Although there is novelty in the method and results, authors fail to make this novelty sufficiently clear. First, regarding the novelty of the method, the contrast with methods used so far in the literature and which gap the proposed method addresses is not motivated enough in the Introduction - the paper would greatly benefit from this in its ability to convince the reader. **(2)** Second, regarding the novelty of the results, the paper lacks sufficient explicit contrasts to prior findings on the hubs, connectivity, and streams. Currently, statements are not consistent with the evidence presented - an example is the confirmatory statement "our representational connectivity analysis identifies a stimulus-driven network [..] consistent with the dual-network topology previously reported for scene perception [54]" while the presented evidence shows that the network identified is diverging from the standard dual-network topology. Also in the intro: "The other traces a lateral-dorsal route, selectively tuned to biological and social content, offering empirical support and a data-driven refinement for recent proposals of a 'third visual pathway' for social perception [29,30]": **(3)** the current results are only showing up lateral, not dorsal. The authors should more clearly delineate when results diverge from prior literature rather than stressing only commonalities. **(4)** It is also important to comment on to what extent their language vs. vision model mappings

diverge from recent literature by Wang et al., 2023 and Popham et al., 2021, who argue for more language-aligned representation in the higher ventral stream (in NSD) and on the 'border' of visual cortex, respectively.

We thank the reviewer for this insightful critique. We agree that the original manuscript did not make the methodological novelty sufficiently explicit, and that some statements overemphasized consistency with prior literature rather than clearly delineating where our results diverge.

In response, we have substantially revised the Introduction, Results, and Discussion. We address the four specific points raised below:

1. Clarifying the Methodological Gap. We have revised the Introduction (L39–41) and Discussion (L331–335) to clearly motivate why we propose this specific framework. We clarify that while existing methods effectively localize shared representations (e.g., IS-RSA) or describe static connectivity (e.g., resting-state), they are rarely integrated to define functional networks driven by specific content. Our contribution is the integration of these complementary analyses to move from region-level correspondences to a network-level organization, identifying not just where representations are shared, but how they route information through the cortex.

2. Explicit Contrasts to Prior Findings. We acknowledge the critique that we previously overemphasized commonalities with standard dual-stream models. In the revised **Discussion** (L263–288), we now explicitly contrast our findings with prior literature. We clarify that the network topology we identify is not merely a confirmation of the standard dorsal/ventral split, but a refinement that highlights a stimulus-driven functional dissociation between a ventromedial scene-integration hub and a distinct lateral-temporal route. Furthermore, we have updated the **Results** to reference recent literature identifying functional pathways consistent with those observed in our work.

3. Lateral vs. Dorsal Terminology. We thank the reviewer for this important correction regarding the "dorsal" terminology. We have removed references to a "dorsal" route where our data support a lateral pathway, and we now consistently describe this as a Lateral Route centered on LOTC. While the terminology in the original manuscript was intended to reference specific dorso-lateral routes (e.g., Lima et al., 2023) compatible with our findings, we agree that this created ambiguity. In the revised manuscript, we explicitly delineate that while the LOTC-centered network shares features with the lateral "third visual pathway" proposed for social perception and related dorso-lateral proposals, it is distinct from the classical parietal dorsal stream associated with spatial action. We highlight this distinction as a specific divergence from standard dual-stream accounts in the **Discussion** (L263–271).

4. Divergence from Language vs. Vision Model Literature. We thank the reviewer for this observation, which allows us to better position our model alignment results within the context of recent literature.

Regarding Popham et al., 2021, which identifies multimodal representations at the border of vi-

sual cortex, we now explicitly reference this work when presenting our language alignment results (L108–114). While our study relies solely on fMRI data acquired during a visual task (unlike the cross-modal intersection performed by Popham et al.), we observe weak but significant alignment for both language and vision models in anterior regions (e.g., anterior temporal lobe; Fig. 2C-D). This overlap is compatible with the multimodal integration zones outlined by Popham et al.

Regarding Wang et al., 2023, we extensively investigated why our language models did not show the ventral stream alignment reported in other works testing language representations on NSD (e.g., Doerig et al., 2025; Wang et al., 2023). As detailed in our response to **Reviewer 1.Q5**, this divergence is driven by the training objective: *contrastive* models (like CLIP or MPNet used in those studies) explicitly structure embedding spaces by semantic similarity, naturally yielding ventral alignment. We have added a clarification of this objective-dependent difference in the Results (L115–121).

Reviewer #2.Q2 The way the research question sets the scene is a bit unnecessary in the sense that it stresses a debate that the paper does not necessarily answer - the question of whether the architecture of the brain or the statistics of the outside world are more important is not addressed, as here the only focus is on the statistics of the outside. Currently, the Introduction sets up for an 'ideosyncratic vs universal' debate, but the evidence here is only on the universal, leaving the reader to expect evidence for the idiosyncratic side as well. Several somewhat abstract theoretical views/frameworks are invoked (Plato, Gibson), and questions are raised on the relation between brain anatomy and function, individual differences - these do not all seem particularly relevant to the current research question: it would be fine to motivate the paper just from the question as to how best identify networks involved in visual perception.

We thank the reviewer for this constructive critique. We agree that the original framing overemphasized broader theoretical debates (external vs. internal constraints; universal vs. idiosyncratic coding) that are not directly addressed by the analyses in this study. We acknowledge that invoking these perspectives created an expectation for tests (e.g., regarding idiosyncratic coding) that were outside the scope of the paper.

To address this, we have substantially revised the Abstract, Introduction, and Discussion to motivate the study more directly from its concrete methodological goal: identifying and characterizing the cortical networks engaged during visual scene perception using shared representational geometry. In the revised Introduction, we have removed the philosophical framing and the universal vs. idiosyncratic dichotomy. Instead, we now explicitly treat inter-subject convergence as a methodological premise, using it to localize and compare stimulus-locked representational structure across regions and models. This reframing aligns the scope of the manuscript with the evidence presented and clarifies the primary contribution: a network-level characterization of shared visual representations.

Reviewer #2.Q3 The description of the cross-validation findings in BOLD5000 do not exactly reflect the plots, and there are quite a few differences in the plots that are not commented on. Specifically, in 6a the LOTC hub has a "hole" in the middle and in 6b it has a different shape

as well, while EVC seems to be missing completely. In 6c, the layer progression of the Ventral hub is much less flat, showing a clearer peak than in NSD, while the layer progression of LOTC is conversely more flat than in NSD. In 6d, again EVC is missing and LOTC is less constrained to the ROIs that were found in NSD. Although these differences do not "break" the main claims, and also NSD is more reliable in scale and task, these should be commented on and not just overlooked with a mismatching description that "findings were replicated".

We thank the reviewer for this detailed assessment, which is closely related to the Reviewer #1.Q6 comment. We agree that the original description of the BOLD5000 findings did not sufficiently acknowledge or interpret the dataset-specific deviations visible in the plots (including reduced EVC prominence, a less contiguous LOTC pattern, and differences in hub-wise layer profiles).

In the revision, we have rewritten the **Cross-Dataset Comparison** section (Section 2.6) to more carefully describe these differences and to clarify which aspects of the organization are stable across datasets versus which are expected to be regime-dependent. We now explicitly discuss the BOLD5000 deviations noted by the reviewer–such as the weaker alignment in EVC and the irregular morphology of the LOTC hub (the observed "hole")–and attribute these differences largely to variations in signal reliability and stimulus duration relative to NSD. We have updated the text to avoid phrasing that implies exact replication, instead framing these results as showing qualitative consistency of the major functional hubs subject to dataset-specific signal constraints.

Reviewer #2.Q4 (1) The way connectivity is modeled (with direction going from low-level to high-level) does not allow for feedback, and so might produce an incorrect picture of information flow. **(2** Also, it is often the case that an ROI has less similarity with itself than with other ROIs; does that put into question the validity of that ROI / reproducibility of it across subjects? These aspects should be commented on more, as well as that similarity is only a proxy for connectivity (and should not be confused with anatomical connectivity, for example).

We thank the reviewer for raising these important points regarding the interpretation of the connectivity results.

1. Directionality as a hierarchical proxy. Regarding directionality in the connectivity graph, we agree that our approach does not model feedback loops or causal information flow. As noted in the manuscript, edge direction is derived solely from hierarchical correspondence with vision models (peak alignment depth) and is used as a descriptive proxy for representational level, taking the low- to high-level feature hierarchy of feedforward vision models as a reference. In the revised manuscript, we further emphasize this point in Section 2.4 (L155–156) to avoid interpreting the arrows as anatomical or causal connectivity.

2. Off-diagonal similarity and reliability. We agree that cases where parcels show higher IS-RSA with other parcels than with themselves deserve explicit discussion. We do not interpret this as evidence against ROI validity or reproducibility, but rather as a consequence of IS-RSA reflecting both (i) similarity in the underlying (noise-free) representational geometry between two

regions and (ii) region-specific measurement reliability.

Under a standard signal attenuation model, the observed inter-subject representational similarity between regions r_i and r_j can be approximated as:

$$IS\text{-}RSA(r_i, r_j) \approx \rho_{ij} \cdot \sqrt{rel(r_i) \cdot rel(r_j)} \quad (3)$$

where ρ_{ij} is the latent noise-free correlation and $rel(\cdot)$ represents reliability. While the maximum latent correlation ρ_{ii} is 1, the *observed* magnitude $IS\text{-}RSA(r_i, r_i)$ can be lower than the cross-correlation with a neighbor r_j if that neighbor has significantly higher reliability ($rel(r_j) \gg rel(r_i)$). This pattern is not specific to IS-RSA; a similar effect is observed in within-subject RSA connectivity (Supplementary Fig. 3).

Empirical validation. In the bootstrap analysis reported in response to Reviewer #1.Q4 (Rebuttal Fig. 2), we confirmed that reliability increases monotonically with IS-RSA magnitude, supporting that high IS-RSA reflects the conjunction of reliable fMRI signal and strong shared representational structure. Consistent with the attenuation model, for every parcel r_i whose maximum IS-RSA is with another region r_j rather than itself, we confirmed that $IS\text{-}RSA(r_j, r_j) > IS\text{-}RSA(r_i, r_i)$. This relationship held for all 180 regions of the HCP-MMP atlas in the NSD data. This supports the interpretation that off-diagonal maxima are driven by reliability gradients.

Normalization control. To further assess this, we computed a normalized connectivity matrix in which each entry was divided by the geometric mean of the corresponding diagonal terms (using

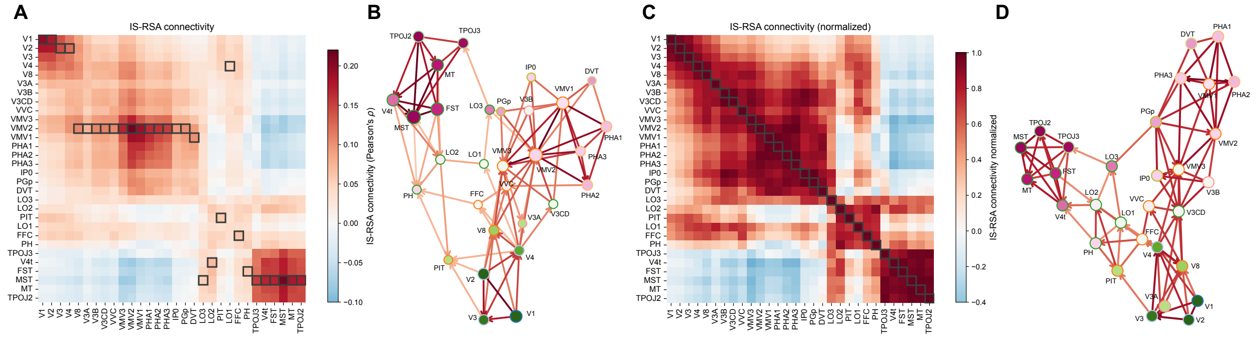

Rebuttal Figure 5: Effect of reliability on IS-RSA connectivity. (**A**) IS-RSA connectivity matrix (Pearson's ρ) for the 30 parcels with highest inter-subject alignment (same set as in the main text). Black squares mark, for each ROI (column), the ROI with which it reaches maximal IS-RSA. (**B**) Directed connectivity graph derived from (**A**) by extracting the backbone with three iterations of a minimum-spanning-tree procedure, reproducing the dual-stream topology reported in the main manuscript. (**C**) Normalized connectivity matrix where each value is divided by the geometric mean of the diagonal self IS-RSA (noise ceiling), effectively setting the diagonal to 1. (**D**) Connectivity graph extracted from the normalized matrix in (**C**), showing that the core dual-stream architecture is preserved after normalization, indicating that the inferred network is robust to reliability differences across parcels.

self-similarity as the noise ceiling):

$$\widehat{IS\text{-}RSA}(r_i, r_j) = \frac{IS\text{-}RSA(r_i, r_j)}{\sqrt{IS\text{-}RSA(r_i, r_i) \cdot IS\text{-}RSA(r_j, r_j)}} \tag{4}$$

This normalization effectively sets the diagonal to 1. While this sharpens the pattern in lower-reliability areas, it preserves the overall topology of the network (Rebuttal Fig. 5C). The graph extracted from this normalized matrix recovers the same dual-stream network observed in the main analysis (Rebuttal Fig. 5B,D).

These results indicate that while raw IS-RSA scales shared content by reliability, the discovered network structure is robust to this weighting. We have included a discussion of these results in Section 4.2 (L165–167) to explicitly address how reliability influences off-diagonal connectivity. We maintain the unnormalized IS-RSA connectivity in the main text for direct interpretability but have added the normalized connectivity analysis to the Supplementary Material (Supplementary Fig. 6) and reference it in Section 2.4.

Reviewer #2.Q5 L218: "In Early Visual Cortex (Figs. 5a and D8), stimuli formed a category-free cloud organized primarily by low-level visual features" this is not apparent in the figures. Also the figures show 2 components, while the text says "We focused our analysis on the first KMCCA component".

We thank the reviewer for these observations.

Regarding the organization of the KMCCA components in Early Visual Cortex (EVC), we acknowledge that the low-level structure is not visually explicit in the scatter plots. Our interpretation was based on the absence of semantic clustering (derived from the categories in Fig. 5A) and on the atlas-level visualization in Supplementary Fig. 9. We did not elaborate on this result because the organization of V1–V4 by retinotopic and low-level similarity in the NSD dataset has already been reported in the literature (e.g., Gifford et al., 2025). We have now clarified this point and added this reference (L197–198).

We also acknowledge the ambiguity regarding the number of components. The scatter plots in Fig. 5A–C display the first two components for visualization purposes, whereas the quantitative analysis (Partial RSA; Fig. 5D) relies specifically on the first component. We have revised the text to make this distinction between the 2D visualization and the 1D statistical analysis explicit.

Reviewer #2.Q6 L237: "These results showed that shared representational geometry is a robust framework for revealing the specific cortical networks where stimulus information is processed across both individuals and models"; this statement seems a bit circular. Can the authors comment on how much of the shared representational geometry could be driven or influenced by the quality of the measurement, i.e. the presence of a good fMRI signal in a given area, which is a prerequisite in order to capture brain activity with a model? (which is the logic behind noise ceiling computations).

We thank the reviewer for pointing out that the original sentence was vague and risked sounding

circular. We have revised the Discussion to make the claims more precise and grounded in the empirical results. Following the reviewer's suggestion, we now explicitly acknowledge that shared representational geometry depends jointly on the quality of the fMRI signal and the presence of shared representational content across individuals (Discussion, L313–329).

We also link the impact of signal quality to the empirical power-law relationship we observed between RSA measures. As detailed in Supplementary Note 4 and in our response to Reviewer #1.Q3, this relationship formalizes how measurement noise introduces a systematic, non-linear attenuation of RSA scores. We believe this helps clarify how signal reliability shapes the magnitude of IS-RSA and how this interacts with the underlying representational content, emphasizing that reliability must be considered when interpreting RSA measures in fMRI data.

Reviewer #2.Q7 The last two paragraphs of the Discussion (from L307 onwards) use a lot of impressive language, but lack concreteness. Exactly what is the envisioned 'path for 'hypothesis generation across sensory, cognitive, and cross-modal domains'? What 'mechanistic understanding' and 'mechanistically testable hypotheses about information processing in the brain' does this work yield? One issue here is that it's not quite clear what the authors refer to with 'our explainability analysis' (see also comment below).

We thank the reviewer for this constructive critique. We agree that the original text used overly abstract language. We have substantially revised the Discussion to remove these vague claims and to ensure concreteness. Our intended point was that large, high-quality fMRI datasets, combined with representational methods such as IS-RSA and dimensionality reduction, provide a quantifiable way to identify *which* stimulus features are routed through specific cortical pathways. In the revised Conclusion (L331–349), we now provide more direct and clear directions, avoiding generic claims of "mechanistic understanding."

We also agree that the term "explainability analysis" was ambiguous. We have replaced this term throughout the manuscript with specific descriptions of the method used, referring to it now as KMCCA-based decomposition or feature-space decomposition. This clarifies that we are referring specifically to the projection of model features onto the shared neural components to identify dominant drivers (e.g., scene integration vs. social features).

Minor comments

Reviewer #2.q1 The use of past tense does not work well throughout the paper, e.g. "The directed graph, which highlights the network's main connections, made the two-stream topology of information flow explicit".

We thank the reviewer for this comment. We have revised the manuscript to correct inconsistent uses of past tense.

Reviewer #2.q2 It is stated that the model-brain alignment metrics are based on multiple models,

but it's not clear from figures 2E-G how the model dimension features in these analyses- are only the best models reported, or the average? Are the dots in E models, parcels, or subjects? Same for Fig 3.

We thank the reviewer for requesting this clarification. The figures report values averaged across models. Specifically, in Figures 2E–G and 3, each data point represents a single HCP parcel. The values plotted reflect the group-level average alignment, computed by first averaging across models within the respective modality (vision or language) and then averaging across all subjects.

While this procedure was described in the Methods, we agree it should be immediately clear to the reader. We have revised the captions for Figure 2 and Figure 3, as well as the methodological descriptions in Sections 2.1 and 2.2, to explicitly state how this aggregation is performed.

Reviewer #2.q3 Figure 5 "(E, F) Cortical maps of inter-subject RSA recomputed separately for those scenes without (E) versus with (F) biological agents (people or animals)" here E and F are flipped. Also in 5d, it is not clear that orange is with the 1st dimension removed in how it is phrased in the legend.

We thank the reviewer for identifying these inconsistencies. We have corrected the caption in Figure 5 to properly match panels E and F.

We have also revised the legend in Figure 5D to explicitly distinguish the conditions, using the labels "Baseline IS-RSA (uncontrolled)" and "Partial IS-RSA (controlling for KMCCA 1)" to ensure clarity.

Furthermore, while reviewing this figure to address your comment, we identified a plotting error in the original manuscript: the data displayed in Figure 5D inadvertently showed within-subject RSA values rather than the intended inter-subject RSA values due to a versioning mismatch. We have corrected this in the revised manuscript to show the correct IS-RSA values. We confirmed that the qualitative pattern remains unchanged and the same conclusions hold. We are grateful to the reviewer for prompting the scrutiny that allowed us to rectify this.

For reference, the updated figure is included at the end of this document (Rebuttal Fig. 7).

Reviewer #2.q4 Figure 2b, a suggestion to keep orange as the color of Ventral hub for consistency

We thank the reviewer for this suggestion. We have updated Figure 2B to use orange for the Ventral hub, ensuring consistency with the color scheme used throughout the manuscript.

For reference, the updated figure is included at the end of this document (Rebuttal Fig. 8).

Reviewer #2.q5 L 88: There are many diferent ways to define 'scene statistics'; what is meant with the term here, exactly?

We thank the reviewer for requesting this clarification. We agree that the term "scene statistics" was vague. In this context, we use it to refer to the statistical structure of natural images at different levels, ranging from low-level visual regularities (e.g., spatial frequency) to higher-level

semantic patterns (e.g., layout, object co-occurrence, animacy). Our intent was to describe the empirical regularities consistently exploited by both biological and artificial visual systems, rather than a specific formal model. We have revised the manuscript to replace this term with more precise terminology as part of the language revision.

Reviewer #2.q6 L272: 'THINGS only engaged EVC' - This seems too strong of a statement, and should be qualified with comparisons to previous papers that used THINGS - did they only find early visual cortex involved in object perception also?

We thank the reviewer for this important correction. We agree that the original phrasing "THINGS only engaged EVC" was imprecise and overly strong. Our intention was to describe the relative distribution of peak alignment in our specific analysis pipeline, which appeared primarily confined to early visual cortex compared to the broader coverage observed in NSD and BOLD5000.

However, as the reviewer notes, prior work using THINGS-fMRI (e.g., Hebart et al., 2023) has robustly demonstrated that object identity is decodable from higher-level ventral and temporal cortices. In the revised Comparison section (Section 2.6), we clarify that while our IS-RSA pipeline detects the strongest shared signal in EVC for this dataset, this reflects a specific sensitivity profile of the method and dataset rather than an absence of object representations in higher-level regions.

Reviewer #2.q7 It would be interesting to also see the directed graphs for the validation dataset (Fig. 6).

We thank the reviewer for this suggestion. We have updated Figure 6 in the comparison section to include the corresponding backbone connectivity graphs for the validation datasets (BOLD5000 and THINGS), and we now reference these results in Section 2.6.

For reference, the revised Figure 6 is included at the end of this document (Rebuttal Fig. 9).

Reviewer #2.q8 L277 mentions 'explainability techniques', but it's not really clear what is meant here - the dimensionality analysis?

We thank the reviewer for this comment. We confirm that the term "explainability techniques" referred specifically to the dimensionality decomposition analysis (KMCCA). We agree that the original phrasing was ambiguous. As part of our general revision of the Discussion, we have replaced this term with more precise descriptions (e.g., "KMCCA-based decomposition" or "feature-space decomposition") to ensure clarity.

Reviewer #2 - Textual Comments

- Line 30: "experiental" spelling error
- L 63 : 'this cortical map' - not clear what 'this' refers to
- L 66: not clear what 'this prediction' refers to
- L 69: the acronym HCP-MMP needs to be introduced

- L 71: ‘quantify the representational alignment across. . . .’ [subjects]
- L 83: ‘to. . . ’ categorization?
- Line 83: the sentence ending in “to40,41” is left unfinished
- Line 205 “Standard RSA”; specify “Standard IS-RSA”
- Line 242 “NSD picture dataset” weird phrasing
- L238 ‘was significantly reduced’ - what was reduced?

We thank the reviewer for these detailed textual comments. Many of the specific line-referenced issues have been resolved as part of the broader rewrite of the manuscript (and some of the referenced sentences no longer appear in the revised version). We have nonetheless checked each item and ensured that all corresponding typos, unclear references, missing definitions (e.g., HCP-MMP), and incomplete phrases have been corrected in the revised manuscript.

References

Doerig, A., Kietzmann, T. C., Allen, E., Wu, Y., Naselaris, T., Kay, K., & Charest, I. (2025). High-level visual representations in the human brain are aligned with large language models. *Nature Machine Intelligence*, 1220–1234. https://doi.org/10.1038/s42256-025-01072-0

Gifford, A. T., Jastrzębowska, M. A., Singer, J. J. D., & Cichy, R. M. (2025). In silico discovery of representational relationships across visual cortex. *Nature Human Behaviour*, *9*(10), 2079–2098. https://doi.org/10.1038/s41562-025-02252-z

Hebart, M. N., Contier, O., Teichmann, L., Rockter, A. H., Zheng, C. Y., Kidder, A., Corriveau, A., Vaziri-Pashkam, M., & Baker, C. I. (2023). THINGS-data, a multimodal collection of large-scale datasets for investigating object representations in human brain and behavior. *eLife*, *12*, e82580. https://doi.org/10.7554/eLife.82580

Lima, B., Florentino, M. M., Fiorani, M., Soares, J. G., Schmidt, K. E., Neuenschwander, S., Baron, J., & Gattass, R. (2023). Cortical maps as a fundamental neural substrate for visual representation. *Progress in Neurobiology*, *224*, 102424. https://doi.org/https://doi.org/10.1016/j.pneurobio.2023.102424

Popham, S. F., Huth, A. G., Bilenko, N. Y., Deniz, F., Gao, J. S., Nunez-Elizalde, A. O., & Gallant, J. L. (2021). Visual and linguistic semantic representations are aligned at the border of human visual cortex. *Nature Neuroscience*, *24*(11), 1628–1636. https://doi.org/10.1038/s41593-021-00921-6

Wang, A. Y., Kay, K., Naselaris, T., Tarr, M. J., & Wehbe, L. (2023). Better models of human high-level visual cortex emerge from natural language supervision with a large and diverse dataset. *Nature Machine Intelligence*, *5*(12), 1415–1426. https://doi.org/10.1038/s42256-023-00753-y

Additional Revised Figures

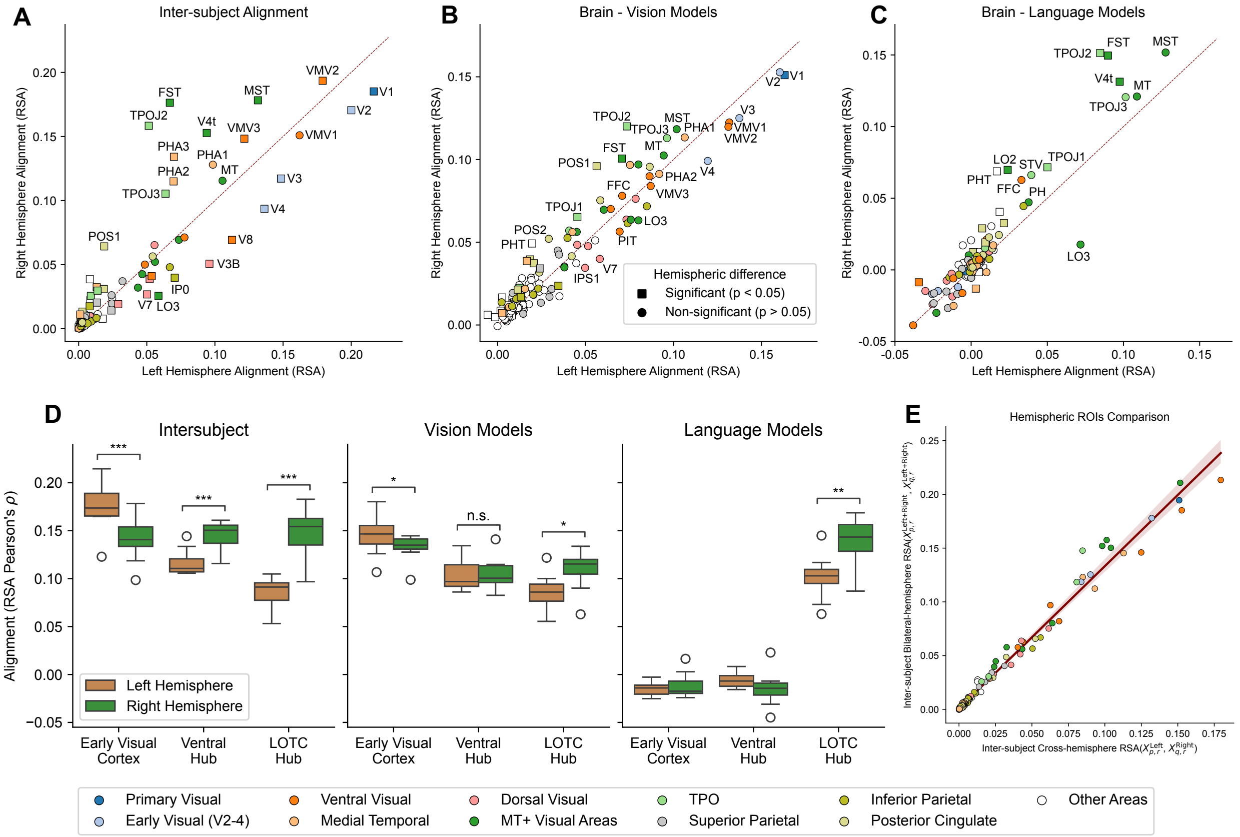

Rebuttal Figure 6: Hemispheric comparison of representational alignment. Revised version of Supplementary Figure 15. **(E)** This panel has been restored to the figure, displaying the correlation between cross-hemispheric and bilateral inter-subject alignment, which was inadvertently omitted in the previous version. Panels **(A–D)** remain unchanged.

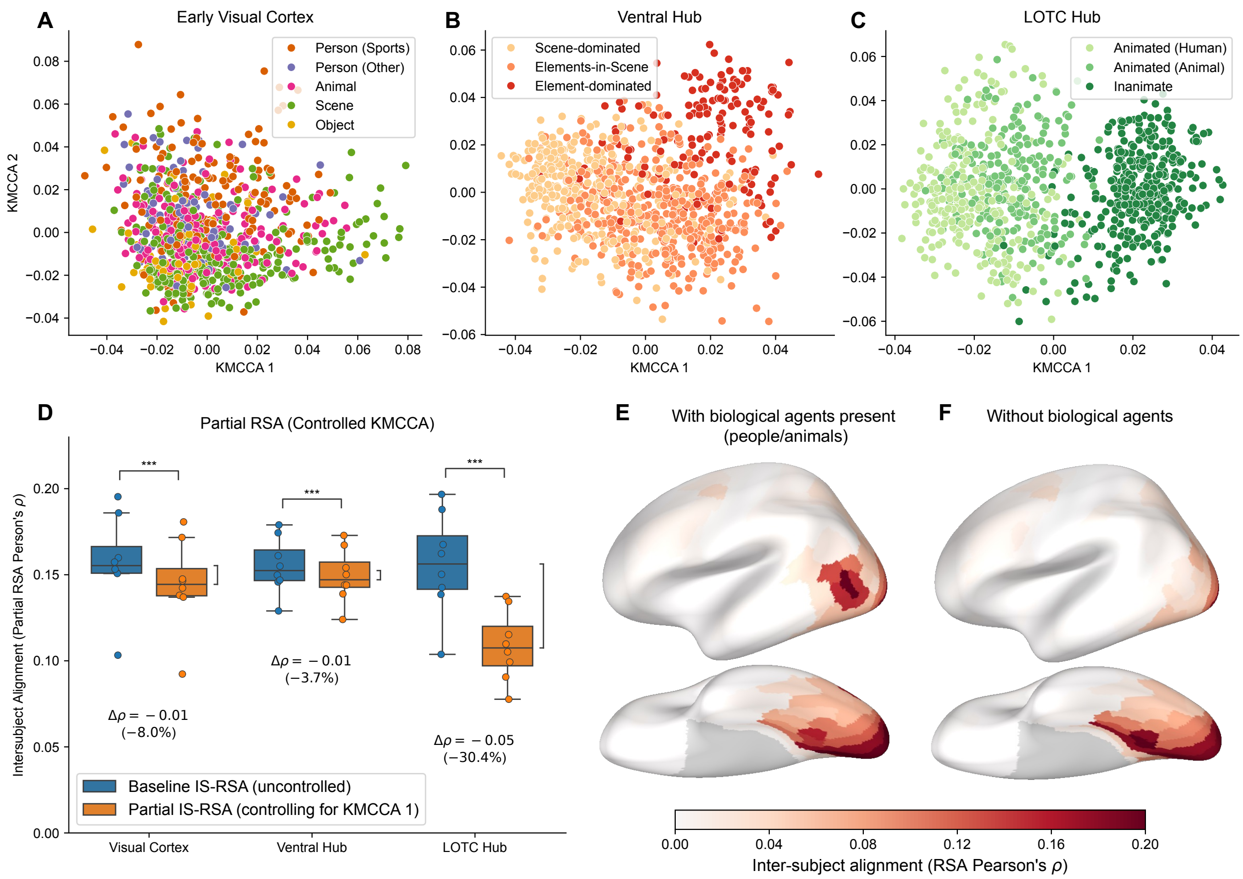

Rebuttal Figure 7: KMCCA components and biological-content dependence of LOTC shared geometry. Revised version of Figure 5. **(D)** The data has been corrected to display inter-subject RSA values (previously inadvertent within-subject values), as noted in the response to Reviewer #2. **(E–F)** The caption labels have been swapped to correctly match the panels: **(E)** now correctly refers to scenes *with* biological agents, and **(F)** to scenes *without* biological agents. Remaining panels are unchanged.

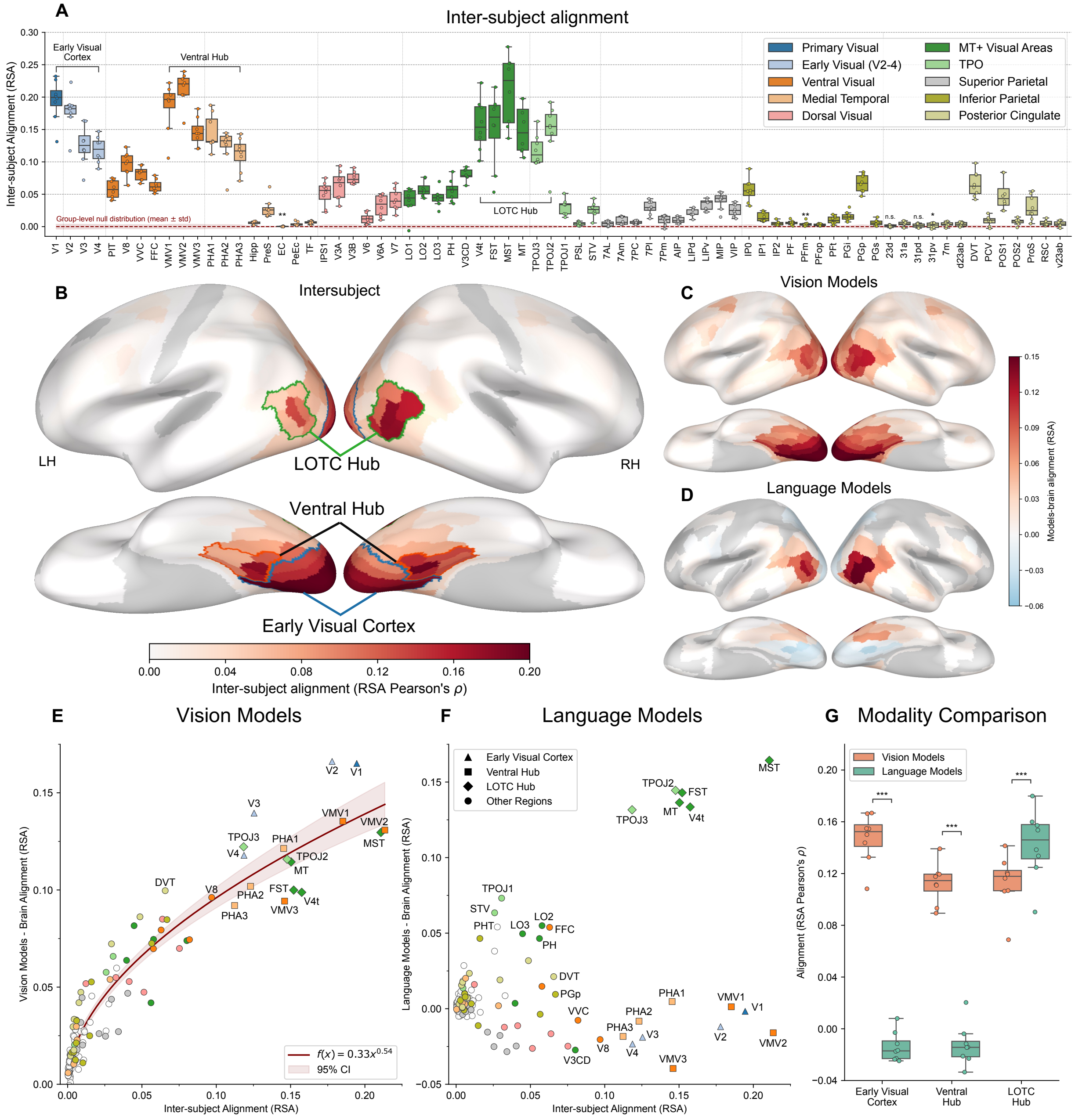

Rebuttal Figure 8: Inter-subject and model–brain alignment across cortex. Revised version of Figure 2. Panel **(B)** has been updated to use an orange color scheme for the Ventral hub to ensure consistency with the rest of the manuscript, as requested. The data and remaining panels are unchanged.

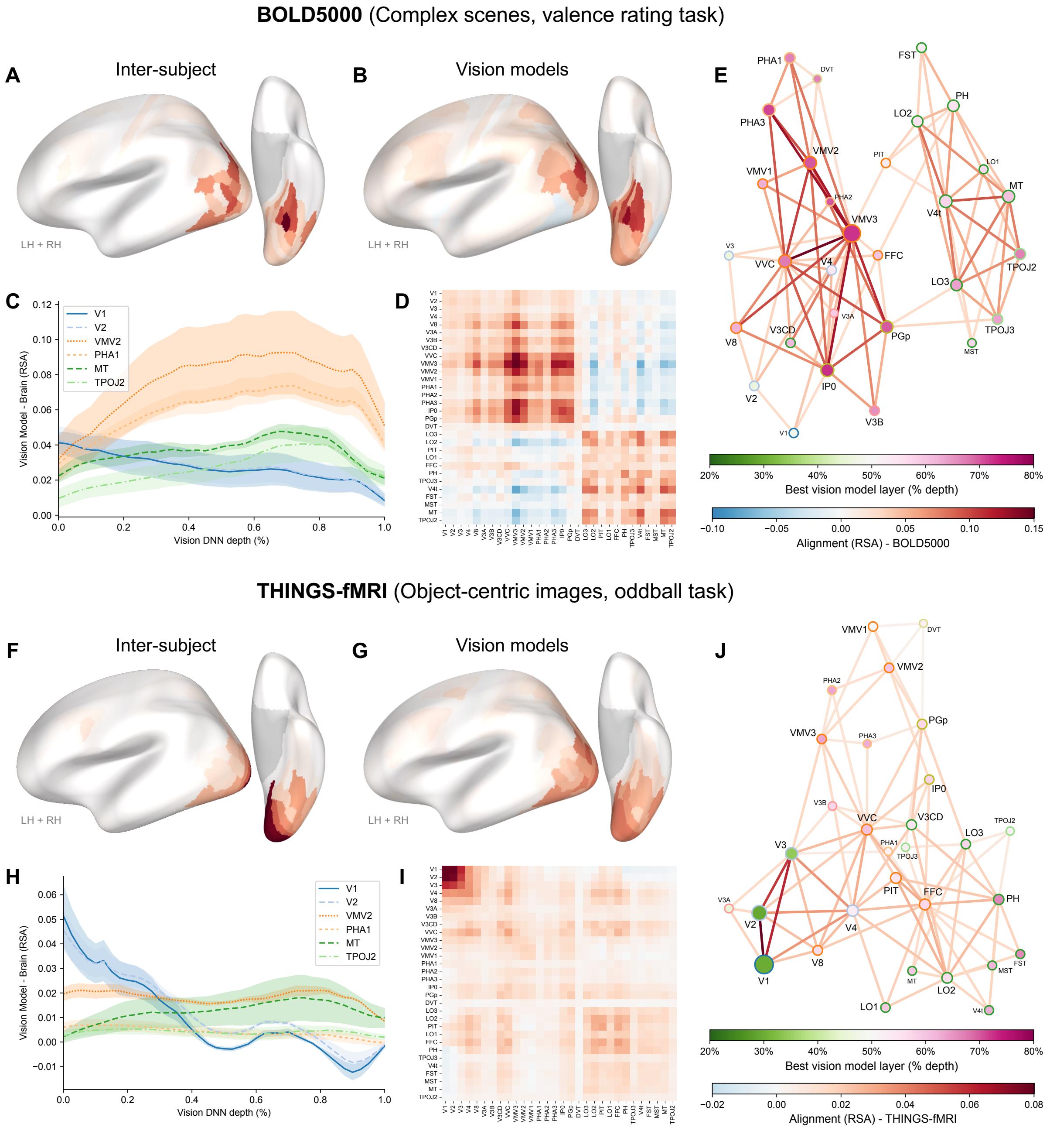

Rebuttal Figure 9: Comparison across independent datasets. Revised version of Figure 6. As requested, backbone connectivity graphs have been added for both validation datasets. **(E)** Backbone graph for BOLD5000, recovering the dual-stream topology. **(J)** Backbone graph for THINGS-fMRI, showing a contracted network structure without clear stream segregation. The remaining panels (alignment maps and connectivity matrices) remain unchanged.

Response to Reviewers

Manuscript ID: COMMSBIO-25-7053-T

Journal: *Communications Biology*

Title: *Shared representations in brains and models reveal a two-route cortical organization during scene perception*

Dear Editor and Reviewers,

We sincerely thank you for this second careful evaluation of our manuscript, following the substantial reframing introduced in the previous revision, and for the constructive comments and suggestions.

In response to the remaining concerns raised by Reviewer #2, we have carried out a further revision of the manuscript focused on three main points: clarifying the novelty of the study relative to prior work, directly evaluating the potential influence of signal-to-noise differences, and refining the Discussion to more clearly separate empirical findings from interpretation, particularly with respect to the proposed role of the dorsal pathway.

We have also updated the public data repository to include the new derivatives generated for this revision, including the additional signal-to-noise analyses (10.6084/m9.figshare.30753239), and we have updated our public code repository with the scripts used for the revised analyses (github.com/memory-formation/shared-representations).

In the attached revised manuscript, the sections modified during this revision are highlighted in blue. Below, we provide a point-by-point response to each comment. In this document, each reviewer comment is reproduced in blue, followed immediately by our response.

Reviewer #2

Major comments

Comment 1 (Q1)

(i) One concern is that the novelty of the outcomes is (still) not sufficiently clear. Results sections 2.1 and 2.2. seem entirely confirmatory, except for line 115 where the negative correlation of pure-language models is shown. We believe there are novel insights in the paper, but they are not coming through clearly enough - we feel this is especially important to pay attention to since it is using the open dataset NSD, which has already been analysed by many researchers.

We thank the reviewer for this constructive suggestion and agree that the novel contributions of the manuscript should be stated more clearly, particularly given the extensive prior use of NSD. In the original version, the language-model results were not sufficiently emphasized because they were initially introduced mainly as a modality control. We agree that the negative RSA of pure language models in early visual and ventral regions is a novel result and should be highlighted explicitly. We have therefore revised the Abstract (L11), Introduction (L51-52), and Results Section 2.2 (L125-131) to state this contribution more clearly.

https://naturalscenesdataset.org/ maintains a list of papers that use NSD. Can the authors check the following:

(ii) Are they the first NSD paper to directly contrast vision with pure language models, also in terms of layer depth alignment?

To the best of our knowledge, yes. No previous NSD study has directly contrasted *pure* vision and *pure* language models within a unified *layer-wise RSA* analysis.

Prior NSD studies have compared vision and language-related models mainly using voxel-wise encoding approaches, often focusing on final-layer representations, or have used RSA for vision and vision–language models without placing pure language and pure vision models into the same layer-wise comparison. For example, Wang et al., 2023 compared ResNet, CLIP, and BERT using voxel-wise encoding rather than RSA; Conwell et al., 2024 performed extensive RSA analyses across vision and vision–language model families, but did not include pure language models; and Doerig et al., 2025 reported RSA alignment for a pure language model (MPNet), but did not directly contrast *autoregressive* language models and pure vision models within a common layer-wise RSA framework.

We have emphasized this point explicitly in the revised Abstract (L6), Introduction (L42-44) and Results (L92), where we now state that our approach provides a direct layer-wise RSA contrast between pure vision and pure language model families across cortex.

(iii) Are they the first to derive a connectivity graph based on IS-RSA and populate it with VM/LM-RSA to derive hierarchies?

To the best of our knowledge, yes. Based on the prior NSD studies listed and our broader literature review, we are not aware of previous work that derives a cortical connectivity graph from inter-subject representational similarity and then interprets that graph using model hierarchies. Although the use of model hierarchies as a proxy to infer ordering in cortical processing has clear precedents in the literature (e.g. Cichy et al., 2016), and representational similarity has also been used as a measure of connectivity (e.g. Pillet et al., 2020), we are not aware of previous work that combines both approaches within the NSD literature.

Prior NSD studies have examined related components separately. For example, Gifford et al., 2025 derived brain–brain representational networks, but did not populate or interpret these networks using model–brain alignment hierarchies. Other NSD studies performed model–brain alignment analyses, but without integrating these with an IS-RSA-derived cortical graph. To our knowledge, therefore, no prior NSD study has derived a connectivity graph from inter-subject representational similarity and interpreted that graph using model alignment. We now state this integration of representational connectivity and model hierarchy explicitly in the Abstract (L6), Introduction (43-44), and Results (197-200).

If so, clearly state this as a contribution of the paper. Here are some other specific insights that seem novel to us:

(iv) L119: "Unlike the next-token…" this seems to be providing a proposition for why the results deviate from what the literature has claimed so far, but it is phrased as if it is a result, rather than an explanation/novel insight. This should be more clear (e.g "we propose that…").

We agree that this sentence was phrased too strongly as an empirical conclusion. We have revised the text to make clear that this is a proposed interpretation of the observed dissociation, rather than a direct result established by the present analyses (L127-131).

(v) Same for L150 "under this input mismatch…' this seems to convey a really interesting, potentially novel insight on NSD model alignment, but the way it's formulated it's again a bit ambiguous if this is an empirical finding that is supported here, or a proposed explanation. And do we understand correctly that Supplementary Note 5 suggests that the language alignment is purely driven by token frequency? Isn't this a profound insight, arguing against a high-level abstract 'linguistic' representation in LOTC?

We thank the reviewer for highlighting this distinction. We agree that the original wording could be read too strongly, as if the "input mismatch" account were itself a direct empirical result, whereas it is more appropriately framed as a proposed interpretation of the observed layer-wise dissociation between vision and language models.

Regarding Supplementary Note 5, our intention was not to claim that LOTC language alignment is *purely* reducible to token frequency. Rather, the tokenizer-based control analyses show that token-occurrence structure explains a substantial portion of the observed language–brain correspondence. In particular, tokenizer-based RDMs closely reproduce the spatial localization of language-model

alignment, correlate strongly with first-layer alignment, and also correlate with the maximum alignment observed across layers. We therefore interpret these results as evidence that a large fraction of the alignment can be explained by relatively shallow lexical statistics or bag-of-words-like structure, rather than requiring progressively composed syntactic representations. This interpretation is also consistent with earlier observations that high-level visual cortex can align with semantic similarity spaces derived from relatively simple lexical-semantic models, including WordNet-based analyses in occipitotemporal cortex and semantic category maps spanning visual and non-visual cortex (Carlson et al., 2014; Huth et al., 2012).

We agree that this is an informative result, and we have revised both the main text (L165–168) and Supplementary Note 5 (L965-985) to make this interpretation clearer while keeping it appropriately qualified.

(vi) Also, among the vision models group here there are CLIP models, so how does it compare to the other, 'purely' vision models (i.e. do the authors reproduce the claim of Wang et al. (2023)?). In the rebuttal, the authors provide an explanation for why contrastive models like CLIP align more with the ventral stream, but it's not clear whether they find any distinction between the vision(-y) models themselves and how this relates to the poor language alignment in ventral but strong alignment in LOTC - we think this can be seen in Supplementary Figure 5 but it would be worth commenting on explicitly in the main text.

We thank the reviewer for this important point. Following this suggestion, we have added a brief comment in the main text (Revised manuscript, lines 145–151) explicitly describing the differences across vision-model families and directing the reader to Supplementary Fig. 5, where these profiles are shown in detail.

Across vision-model families, the overall layer-wise alignment patterns were qualitatively similar. In most cases, early visual cortex aligned more strongly with earlier layers and then declined with depth, LOTC alignment increased with depth, and ventral alignment remained relatively stable or peaked at intermediate-to-late layers. The clearest family-level deviation was the MAE family, which retained relatively strong alignment with early visual cortex even in later layers, plausibly reflecting its image-reconstruction objective. By contrast, CLIP-family vision encoders did not show a qualitatively distinct profile from the other vision-model families. Rather, they remained much closer to the common vision-model pattern, with substantial ventral alignment and increasing LOTC alignment in later layers, than to the profile observed for pure autoregressive language models. This indicates that the ventral–LOTC dissociation in our results is more closely linked to the training objective and representational format of pure next-token language models than to the mere presence of language supervision.

Regarding Wang et al., 2023, we do not view the two analyses as directly equivalent, since Wang et al. used voxel-wise noise-ceiled encoding models whereas our study uses RSA to compare representational geometry across stimuli, regions, and model layers. These approaches probe related but distinct properties. That said, our results are broadly compatible with their conclusions. Wang

et al. reported an advantage of language-supervised contrastive models in higher-level visual regions, especially in lateral occipitotemporal cortex. In our framework, CLIP-family models likewise showed strong later-layer alignment in LOTC. However, we did not observe a markedly stronger overall RSA profile for CLIP-family models relative to the other vision-model families. Instead, most vision-model families showed broadly similar geometric alignment profiles, with the clearest family-level deviation arising for MAE. We therefore interpret our results as complementary to Wang et al.: contrastive vision–language supervision is compatible with strong alignment in higher-level visual cortex, but in the present RSA framework it does not produce a strong separation from other large-scale vision models trained on diverse visual datasets.

Comment 2 (Q2)

The signal quality question, raised in our original review question #2.Q6, is not entirely resolved, in our opinion. Supplementary Note 2 now includes helpful comparisons of how IS-RSA scores of different brain parcels are affected by various resampling schemes of stimuli and repeats, whereby the authors conclude that 'parcels with stronger shared geometry yield more stable IS-RSA scores regardless of their size'. However, the question we raised was not whether parcel size (n voxels) affected the IS-RSA scores, but rather whether the quality of the fMRI signal in a parcel predicts the degree of inter-subject alignment - for example via a direct assessment of the relation between voxel-wise SNR and IS-RSA score. Can the authors report a simple correlation between the average SNR in a parcel and its IS-RSA score? Hereby the metric of SNR should be independent of any model alignment (elsewhere, vision model alignment seems to be treated as a proxy for 'signal strength', e.g. response to 1Q3 - which may not be a valid assumption to make), e.g. something like temporal SNR (how strong is the response to an stimulus image relative to baseline/noise).

We agree that this question required a more direct analysis. To address it, we compared parcel-wise IS-RSA against two complementary signal-to-noise measures that are independent of model alignment: temporal signal-to-noise ratio (tSNR) and the NSD noise-ceiling SNR (NCSNR).

First, following the reviewer's suggestion, we computed tSNR from the preprocessed BOLD time series of the first session for each participant. For each voxel, tSNR was defined within each run as the mean BOLD signal divided by its standard deviation across time, and then averaged across runs, parcels, and subjects to obtain a mean tSNR per ROI (Rebuttal Fig. 1A–B). Homologous parcels were averaged across hemispheres because the resulting maps were highly symmetric. Across parcels, IS-RSA and tSNR were not significantly correlated ($r = 0.048$, $p = 0.51$), indicating that inter-subject representational alignment is not explained by this basic measure of temporal signal quality alone.

We also agree with the reviewer that vision-model alignment should not be treated as a proxy for signal quality. In our previous response, our wording was imprecise: rather than referring to regions of high or low signal quality, we should have referred to regions showing stronger or weaker shared

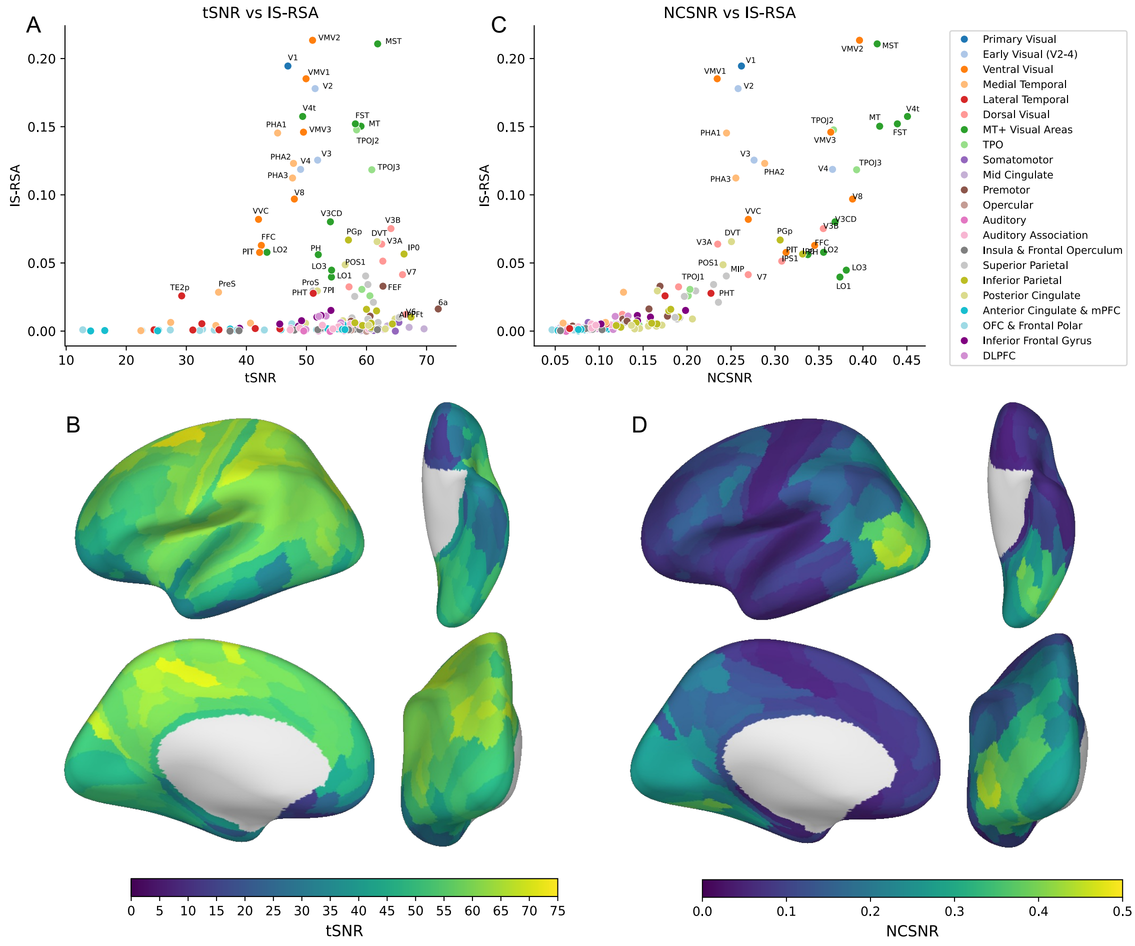

Rebuttal Figure 1: Signal-to-noise metrics. **(A,C)** Scatter plots showing the relationship between parcel-wise signal-to-noise measures and inter-subject representational similarity (IS-RSA). **(A)** Mean temporal signal-to-noise ratio (tSNR) per ROI versus IS-RSA. **(C)** Mean noise-ceiling SNR (NCSNR) per ROI versus IS-RSA. Each point corresponds to one symmetric ROI, obtained by averaging homologous parcels across hemispheres; colors indicate large-scale cortical systems. **(B,D)** Cortical surface maps showing the spatial distribution of the same parcel-wise measures. **(B)** Mean tSNR per ROI. **(D)** Mean NCSNR per ROI. Maps are shown on symmetric cortical parcels, averaging left and right hemispheres, in lateral, medial, dorsal, and ventral views.

representational alignment across metrics.

Second, we compared IS-RSA with the NSD noise-ceiling SNR (NCSNR), the voxel-wise reliability metric provided with the NSD dataset and derived from trial-wise beta responses across repeated presentations of the same image Allen et al., 2022. We averaged voxel-level NCSNR within each ROI and then across participants. As shown in Rebuttal Fig. 1C–D, NCSNR was positively associated with IS-RSA across all 180 ROIs ($r = 0.81$, $p < 0.001$), although this relationship was strongly influenced by parcels with low NCSNR and near-zero IS-RSA. Restricting the analysis to the top 30 ROIs with the highest IS-RSA values, which are the parcels used in the graph analysis, the association was weaker and not significant ($r = 0.24$, $p = 0.19$).

These results indicate that response reliability and inter-subject representational alignment are related but not equivalent. Reliable stimulus-evoked responses are necessary for shared representational geometry to be detectable, but they are not sufficient to explain its spatial distribution across cortex. We have now added Rebuttal Fig. 1 to the Supplementary Information of the revised manuscript (Supplementary Figure 3), and referenced in the Results (L85-88) and Disscusion (L352-372).

Comment 3 (Q3)

The Discussion Section starting on line 262 could benefit from a more explicit discussion of why the classic 'dorsal' route is absent in the current findings. The authors note that their found organization 'does not match classical ventral/dorsal', but then say that this is because the results show 'fractioning into additional routes (l269)' without clearly pointing out that the classic dorsal route is in fact, not found in the current results. If one looks at activation-based intersubject synchrony as reported in classic papers by Uri Hasson, the dorsal pathway (e.g. TOS, POS, IPS) does show high alignment scores (e.g. Figure 1 in Hasson et al., 2004, Science). So why do these dorsal ROIs not show up in the current analyses? **(i)** Could this be due to the use of movies instead of images? **(ii)** Or to their results being based on simple correlations of fMRI activation time-courses, not representational geometries? **(iii)** Or does the shifted analysis introduced here make dorsal synchrony disappear - i.e. are dorsal regions more idiosyncratic across participants, or more driven by task structure than stimulus content? **(iv)** This also ties into our question about signal reliability (2) - if there is high tSNR in dorsal, but no shared representational geometry, that would provide strong evidence the latter is not directly coupled to the signal, and provide stronger support for the claims made on lines 326 ('reliability alone cannot explain') and 328 ('rather than merely tracking').

We thank the reviewer for this helpful clarification. We agree that the original Discussion did not state explicitly enough that the canonical occipito-parietal dorsal route is not recovered as a dominant branch in our representational network, nor did it sufficiently discuss possible reasons for this difference.

In our data, dorsal parcels do show significant alignment, but generally at more moderate levels than the principal hubs identified in the main text (e.g. IPS, POS; Supplementary Fig. 1A). Moreover, in the full-cortex connectivity network (Supplementary Fig. 7), these regions do not emerge as a dominant segregated branch comparable to the ventromedial and lateral pathways. Our analyses do not directly identify the cause of this difference, so we cannot make a strong causal claim. However, the explanations raised by the reviewer are plausible, and we now discuss them more explicitly.

(i) Static images versus movies. We agree that stimulus format is likely a contributing factor. Classical ISC studies such as Hasson et al., 2004 used continuous naturalistic movies, which strongly engage dorsal systems involved in motion, visuospatial updating, action-related computations, and

temporally extended event structure. By contrast, our analyses are based on static scene images, which provide much weaker sampling of these processes. We therefore consider the difference in stimulus regime a plausible contributor to the weaker emergence of a canonical dorsal route in the present data.

(ii) Time-course synchrony versus representational geometry. We also agree that this distinction is important. Classical ISC quantifies similarity in BOLD time courses, whereas our analyses quantify shared representational geometry using denoised single-trial beta estimates. These measures are related, but they probe different properties of the data. It is therefore plausible that dorsal regions show reliable time-course synchrony without exhibiting equally strong shared representational geometry across subjects. In other words, dorsal synchrony may be detectable at the level of temporally correlated responses, yet not translate into a stable cross-subject representational geometry when responses are summarized as pairwise stimulus relationships.

(iii) Effect of the shifted analysis. Our results do not suggest that the shifted analysis is the primary reason for the reduced prominence of dorsal regions. Supplementary Fig. 12, which compares shifted and unshifted IS-RSA, does not show a selective reduction in classical dorsal parcels. Similarly, the comparison between WS-RSA and IS-RSA (Supplementary Fig. 4) does not indicate a disproportionately larger loss of alignment in dorsal regions than elsewhere. We therefore do not think that the absence of a dominant dorsal route is mainly explained by the shifted procedure.

(iv) Relation to signal reliability. We agree that this point is informative. As requested in Q2, we computed parcel-wise tSNR and found that dorsal regions can show relatively high tSNR despite not exhibiting strong IS-RSA (Rebuttal Fig. 1A–B). Together with the NCSNR analysis, this indicates that representational alignment is not directly reducible to basic signal quality alone. Reliable signal appears necessary, but not sufficient, for strong inter-subject representational geometry. This supports our interpretation that the recovered network is not explained solely by regional differences in measurement reliability, but reflects shared stimulus organization.

We have revised the Discussion to state more explicitly that the classical dorsal route is not recovered as a dominant pathway in the present framework, and to briefly acknowledge these possible contributing factors (L296–304).

Minor comments

q1 Line 89: underlay -> underlie

We thank the reviewer for noting this typo. It has been corrected in the revised manuscript (L97).

q2 Line 109 ‘and more weakly in anterior regions of visual cortex’ - which visual hub is referred to here?

We thank the reviewer for pointing out this ambiguity. The phrase was intended to refer to parcels

anterior to the lateral occipitotemporal cortex (LOTC) hub. More specifically, the weaker alignment extends from LOTC into adjacent posterior temporal regions, including areas around the superior temporal sulcus and temporo-parieto-occipital junction (e.g. PGi, STV, and TPOJ1), as well as into ventral occipitotemporal cortex (e.g. LO2, FFC, PH, and VVC), as shown in Fig. 2D. We have revised the text to clarify this anatomical description and avoid confusion (L117–119).

q3 Line 148: ‘this contrast’ ambiguous what it refers to (vision vs. language model alignment?)

We thank the reviewer for pointing out this ambiguity. Here, “this contrast” referred to the difference in alignment patterns between vision and language models. We have revised the sentence to make this explicit (L159).

q4 Line 295: the qualifier ‘coarse’ is repeated several times in the interpretation of the model-brain alignment, but why are the current results evidence for the alignment being based on ’coarse’ dimensions? Could it not be a fine-grained mapping?

We thank the reviewer for this important clarification. Our use of the term *coarse* was intended to describe the level of correspondence that RSA can establish. RSA compares the global similarity structure of two representational spaces across stimuli, so strong alignment can arise from a reduced set of shared organizing dimensions without implying a fine-grained one-to-one mapping between the full spaces. In that sense, RSA is sensitive to dominant shared axes of organization, but does not by itself demonstrate fine-grained or near-isometric convergence between brain and model representations. Recent work is also consistent with the idea that brain–model alignment can often be captured by a reduced set of shared dimensions (Chen and Bonner, 2025), although we do not test this explicitly here.

In addition, part of our use of *coarse* referred specifically to the language-model results. There, early-layer and tokenizer-based controls suggest that a substantial part of the observed alignment can be explained by relatively shallow lexical-semantic structure. In this context, the term was intended to indicate correspondence driven by dominant lexical-semantic dimensions, rather than evidence for progressively composed syntactic or fine-grained representational matching.

We therefore revised the manuscript by removing the term *coarse* throughout, as we agreed that in the present context it could be ambiguous or fail to add interpretive precision. We also revised the relevant passages to distinguish more clearly between empirical results and interpretation (L28, L37, Figure 1 caption, L159, L325, L338).

q5 Line 298: ‘MT/LOTC showed alignment to specific stimulus categories’ > what is this inference based on? The drop in alignment when removing KMCCA 1 in Fig 5D?

We thank the reviewer for pointing this out. Yes, this inference was based on the selective reduction in alignment observed after removing the first KMCCA component in Fig. 5D, indicating that alignment in the MT+/LOTC hub was driven more strongly by stimuli containing animate content. This interpretation is further supported by the analysis restricting the stimulus set to images with

versus without biological agents, where LOTC alignment is strongly reduced in the absence of animacy (Fig. 5E–F; Supplementary Fig. 8). We have revised the sentence to make this interpretation explicit in the manuscript (L330).

q6 Line 310: The interpretation here about 'prediction and compression' seems a bit far-fetched, none of the results presented here show any evidence for such processes happening in the brain. The paper provides evidence for emergence of common representational axes - yes. But the reason why is not clear, there could be many reasons for this not limited to prediction or compression.

We thank the reviewer for this important point. We agree that our study does not provide direct evidence about the mechanisms underlying the emergence of these common representational axes, and that the original sentence was too speculative in invoking prediction and compression. We have revised it to be more cautious, emphasizing the evidence for common representational axes without making claims about their underlying causes (L346–348).

q7 Supplementary Note 4: The provided power-law attenuation is very interesting, but it was not clear to us how it is consistent with the idea that the VM alignment is less noise susceptible (response to R1Q3): why does a high latent rho result in a lower observed similarity compared to the other metrics (Supp Fig 16b)? It is indeed less non-linear power-law like, but also overall much lower than the WS and IS scores.

We thank the reviewer for raising this important point. We agree that our previous wording was not sufficiently precise, because it suggested that the differences between VM-, WS-, and IS-RSA were being attributed to a single notion of "noise susceptibility." In Supplementary Note 4, we model the relation between metrics as

$$\rho_{ij}^{\mathrm{obs}} = a_i \left(\rho_j^{\mathrm{latent}}\right)^{b_i},$$

which explains a large fraction of the relationships between metrics ($R^2 > 0.92$ for all pairs). In this formulation, however, the two fitted parameters capture different aspects of the curves: the exponent b_i reflects the degree of nonlinear attenuation, especially in low-alignment parcels, whereas the scale term a_i controls the overall magnitude of the observed similarity.

Under this interpretation, the fact that VM-RSA shows weaker nonlinear attenuation while remaining overall lower than WS- or IS-RSA is not contradictory. A smaller nonlinear attenuation suggests that VM-RSA is less strongly compressed in low-alignment parcels, whereas the lower overall scale indicates that vision-model alignment captures only part of the shared cortical structure measured by brain–brain comparisons. In other words, VM-RSA can show a shallower attenuation profile while still having a lower absolute magnitude because the models do not capture all of the latent structure expressed in WS- and IS-RSA.

At the same time, this remains an interpretation of the fitted model rather than a directly observed quantity. We therefore agree that ρ_{latent} is better understood not as a direct "true similarity" value from which the observed metrics differ only by noise, but as a latent shared-geometry variable capturing the common parcel-wise ordering across metrics. Each observed metric then reflects

that common latent organization with a different attenuation profile and scale. We have revised Supplementary Note 4 and the Discussion (L350-370) to clarify this distinction and to make the interpretation of the model more cautious.

q8 Rebuttal to #2Q4.2 It is good to observe that with the normalized version of this analysis, the same dual stream network is recovered - however there are some slight differences in connectivity between specific ROIs, so it might be more reliable for readers to view the normalized version (rather than reporting the normalized version only in the Supplement, as the authors decided to do now).

We thank the reviewer for this helpful suggestion. We have now promoted the normalized version of the inter-subject connectivity analysis to the main text by including it in Fig. 5 alongside the unnormalized version, so that readers can directly compare both analyses. During this revision, we also identified a labeling inconsistency in the previous revised version of the manuscript: the normalized graph shown in the Supplementary Information was labeled as IS-RSA, although it had in fact been computed using WS-RSA. We have corrected this, and the normalized version now shown in the main text is the correct IS-RSA-based analysis.

In addition, in Supplementary Fig. 4, which presents the within-subject (WS-RSA) alignment and connectivity analyses, we now also include the normalized connectivity matrix and graph, so that the corresponding robustness check is available for the within-subject measure as well. Copies of the updated figures are included in the Appendix of this response (Rebuttal Figures 2 and 3).

References

Allen, E. J., St-Yves, G., Wu, Y., Breedlove, J. L., Prince, J. S., Dowdle, L. T., Nau, M., Caron, B., Pestilli, F., Charest, I., Hutchinson, J. B., Naselaris, T., & Kay, K. (2022). A massive 7t fMRI dataset to bridge cognitive neuroscience and artificial intelligence. *Nature Neuroscience*, *25*(1), 116–126. https://doi.org/10.1038/s41593-021-00962-x

Carlson, T. A., Simmons, R. A., Kriegeskorte, N., & Slevc, L. R. (2014). The emergence of semantic meaning in the ventral temporal pathway. *Journal of Cognitive Neuroscience*, *26*(1), 120–131. https://doi.org/10.1162/jocn_a_00458

Chen, Z., & Bonner, M. F. (2025). Universal dimensions of visual representation. *Science Advances*, *11*(27), eadw7697. https://doi.org/10.1126/sciadv.adw7697

Cichy, R. M., Khosla, A., Pantazis, D., Torralba, A., & Oliva, A. (2016). Comparison of deep neural networks to spatio-temporal cortical dynamics of human visual object recognition reveals hierarchical correspondence. *Scientific Reports*, *6*(1), 27755. https://doi.org/10.1038/srep27755

Conwell, C., Prince, J. S., Kay, K. N., Alvarez, G. A., & Konkle, T. (2024). A large-scale examination of inductive biases shaping high-level visual representation in brains and machines. *Nature Communications*, *15*(1), 9383. https://doi.org/10.1038/s41467-024-53147-y

Doerig, A., Kietzmann, T. C., Allen, E., Wu, Y., Naselaris, T., Kay, K., & Charest, I. (2025). High-level visual representations in the human brain are aligned with large language models. *Nature Machine Intelligence*, 1220–1234. https://doi.org/10.1038/s42256-025-01072-0

Gifford, A. T., Jastrzębowska, M. A., Singer, J. J. D., & Cichy, R. M. (2025). In silico discovery of representational relationships across visual cortex. *Nature Human Behaviour*, *9*(10), 2079–2098. https://doi.org/10.1038/s41562-025-02252-z

Hasson, U., Nir, Y., Levy, I., Fuhrmann, G., & Malach, R. (2004). Intersubject synchronization of cortical activity during natural vision. *Science*, *303*(5664), 1634–1640. https://doi.org/10.1126/science.1089506

Huth, A. G., Nishimoto, S., Vu, A. T., & Gallant, J. L. (2012). A continuous semantic space describes the representation of thousands of object and action categories across the human brain. *Neuron*, *76*(6), 1210–1224. https://doi.org/10.1016/j.neuron.2012.10.014

Pillet, I., Op de Beeck, H., & Lee Masson, H. (2020). A comparison of functional networks derived from representational similarity, functional connectivity, and univariate analyses. *Frontiers in Neuroscience*, *Volume 13 - 2019*. https://doi.org/10.3389/fnins.2019.01348

Wang, A. Y., Kay, K., Naselaris, T., Tarr, M. J., & Wehbe, L. (2023). Better models of human high-level visual cortex emerge from natural language supervision with a large and diverse dataset. *Nature Machine Intelligence*, *5*(12), 1415–1426. https://doi.org/10.1038/s42256-023-00753-y

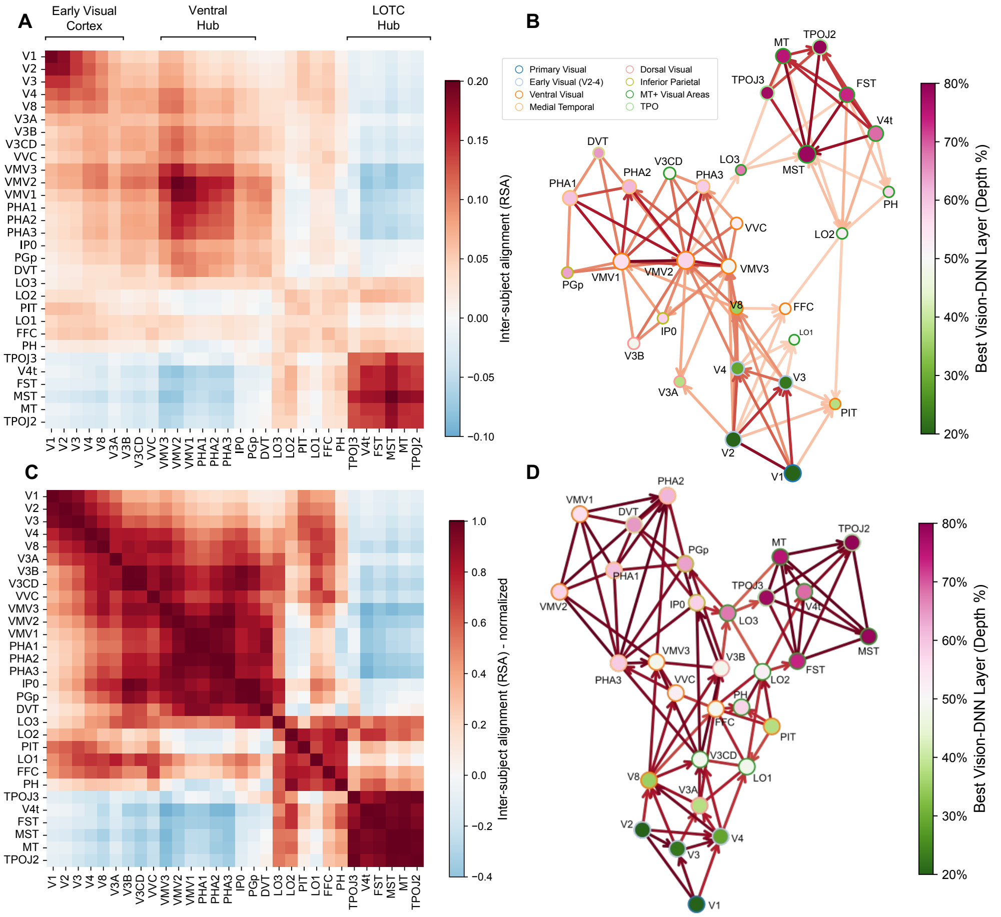

Rebuttal Figure 2: Inter-subject representational connectivity network. (A) Inter-subject connectivity matrix. Parcel-wise representational connectivity (RSA; Pearson's r) for the 30 parcels with the highest mean IS-RSA ($N = 8$). Each cell shows the mean RSA between the RDMs of two parcels, computed across all cross-subject pairs. The matrix shows three clusters corresponding to early visual cortex (V1–V4), the Ventral hub (VMV1–3, PHA1–3), and the LOTC hub (V4t, MT, MST, FST, TPOJ2–3). Warm colors indicate higher representational similarity; cool colors indicate dissimilarity. **(B) Directed connectivity graph.** The same parcels are shown as nodes. Node color encodes each parcel's peak alignment depth with vision models (heuristic proxy for hierarchical position), node size indicates inter-subject alignment strength, and node border color indicates macro-anatomical group membership. Edges correspond to the union of three minimum spanning trees; edge color reflects the RSA value in panel **A**, and edge width indicates the spanning-tree iteration (first to third). Arrow direction is assigned from shallower to deeper peak-depth parcels when the depth difference is significant across subjects (paired t-test, $t(7)$, FDR-corrected $p < 0.05$). The graph highlights two dominant routes from early visual cortex: a medial-ventral route toward the Ventral hub and a lateral route toward the LOTC hub. **(C) Within-ROI-normalized inter-subject connectivity matrix.** Pairwise inter-parcel connectivity values were normalized by the corresponding within-ROI alignment terms to control for differences in overall alignment magnitude across parcels. The normalized matrix preserves the same broad three-cluster organization observed in the unnormalized analysis. **(D) Directed connectivity graph derived from the normalized inter-subject connectivity matrix.** Using the same graph-construction procedure, the normalized analysis recovers a dual-stream topology, with only minor differences in specific ROI-to-ROI connections.

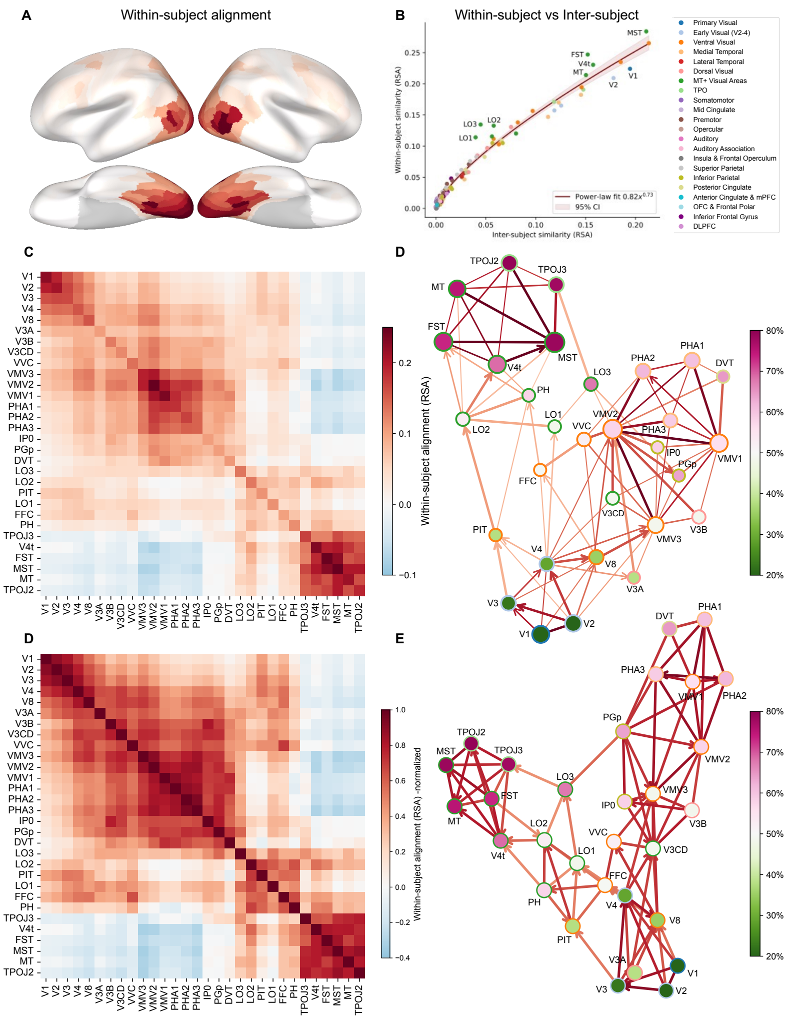

Rebuttal Figure 3: Comparison of within-subject and inter-subject representational analyses. (A) Cortical map of within-subject alignment (RSA, Pearson's r). This was computed analogously to the inter-subject analysis by comparing RDMs from different trial repetitions within each participant, and then averaging across participants ($N = 8$). The map reveals a similar spatial distribution to the inter-subject version, with a posterior-to-anterior gradient and three distinct hubs. **(B) Parcel-wise comparison of within-subject and inter-subject alignment**. The two measures are tightly correlated across the cortex, following a power-law relationship (fit shown in red; $R^2 = 0.98$). This indicates that brain regions showing stronger within-subject consistency also tend to show stronger consistency across individuals. **(C) Within-subject representational connectivity matrix**. Computed by correlating RDMs between all pairs of parcels within each participant, this analysis reveals the same three-hub block structure discovered using the inter-subject measurements. **(D) Directed connectivity graph of the within-subject data**, pruned using a 3-minimum spanning tree to highlight the network backbone. Edge color and width encode the inter-region alignment (RSA value), while node color represents each parcel's peak alignment depth with vision models. Directionality is inferred from this depth measure based on a low-to-high sequential hierarchy, with arrows pointing from shallower- to deeper-aligning regions. The graph confirms the same information flow as the inter-subject analysis, with two streams emerging from early visual cortex: a medial-ventral stream to the Ventral hub and a lateral-dorsal stream to the LOTC hub. **(E) Within-subject representational connectivity matrix normalized by within-ROI alignment.** The normalized matrix preserves the same broad three-hub organization as the unnormalized analysis. **(F) Directed connectivity graph derived from the normalized within-subject connectivity matrix.** The normalized analysis recovers the same dual-stream topology, with only minor differences in specific ROI-to-ROI connections.
